# Adaptive yet suboptimal integration of advice in decision-making

**DOI:** 10.1038/s44271-026-00456-1

**Published:** 2026-05-16

**Authors:** Joshua Zonca, Alice Giampino, Paolo Cherubini, Carlo Reverberi

**Affiliations:** 1 Department of Psychology, Università degli Studi di Milano-Bicocca, Milan, Italy; 2 Milan Center for Neuroscience, Università degli Studi di Milano-Bicocca, Milan, Italy; 3 Department of Economics, Management and Statistics, Università degli Studi di Milano-Bicocca, Milan, Italy; 4 Department of Brain and Behavioral Sciences, Università Statale di Pavia, Pavia, Italy

**Keywords:** Human behaviour, Human behaviour, Social behaviour

## Abstract

Effective use of advice is critical for sound decision-making, yet individuals often fail to fully benefit from external input. Across two experiments, 89 participants performed a perceptual decision-making task while interacting with seven artificial agents characterized by distinct cognitive and metacognitive profiles. By combining a Bayesian modeling approach with experimental data, we establish a normative benchmark to quantify the optimality of advice integration. We show that individuals adaptively adjust their reliance on advice based on the cognitive and metacognitive profiles of the different advisers. While this adaptation generally improves performance, the integration process remains significantly suboptimal, leading to a substantial loss in potential accuracy. This suboptimality is primarily driven by two integration errors. First, an egocentric bias leads decision-makers to overweight their own judgments and confidence signals relative to those of the adviser. Second, a global-to-local deficit prevents them from consistently translating knowledge of each partner’s global traits into trial-level adjustments. These errors persist even when participants are explicitly informed of both their own and the adviser’s attributes, indicating that the bottleneck is not ignorance but flawed information processing. Suboptimality is particularly severe when interacting with higher-quality advisers and varies widely across individuals, tied specifically to their own metacognitive sensitivity and calibration. Our findings reveal fundamental constraints in information integration with implications for both human–human and human–AI-assisted decision-making.

## Abstract (web summary)

Advice improves decisions, yet its use is suboptimal. Combining experiments and Bayesian modeling, this study identifies egocentric discounting and a failure to apply global traits to trial level choices as key causes. Suboptimality peaks with high-quality advisers and varies widely across individuals.

## Introduction

Leveraging others’ advice is a fundamental aspect of human behavior and decision-making^1^. Advice can reduce the risks inherent in individual trial-and-error learning, lower the costs of exploring alternative strategies, and ultimately improve decision accuracy^2,3^. Traditionally, decision-makers across domains have faced a relative scarcity of expert advice due to limited availability of experts, time constraints, or high costs. Recent advances in digital communication and artificial intelligence have dramatically increased both the accessibility and volume of available advice, shifting the primary challenge from access to effective utilization^4,5^. Indeed, greater access does not guarantee that advice will translate into improved decision-making. In contrast, optimal advice integration can substantially enhance outcomes, even exceeding the performance of any individual agent involved in the decision process^6–9^.

Effective advice utilization relies on two core abilities: (1) accurately assessing both one’s own competencies and those of the adviser, and (2) integrating advice optimally, considering this knowledge. Behavioral research indicates that humans partially weigh an adviser’s opinion according to its reliability^5,10,11^, past accuracy^12^, confidence^5,13–15^, expertise^16–18^, and access to relevant information^19^. Nevertheless, humans often disregard or underweight valuable advice. For instance, they tend to underweight socially acquired information^2,7,20–23^, a phenomenon often referred to as Egocentric Advice Discounting^1,24,25^. People assign excessive weight to opinions confirming their original beliefs^26^. Moreover, in joint decision-making, they do not optimally integrate information about others’ confidence or past accuracy^27,28^. This waste of valuable advice also extends to interactions with intelligent artificial agents^29^. A parallel line of research highlights limitations in metacognitive abilities, which influence attributions of competence to oneself and others, thereby constraining advice integration. Experimental evidence points to various forms of metacognitive inefficiency, including low metacognitive sensitivity^30,31^ and overconfidence^32,33^.

Together, previous research revealed several biases and metacognitive limitations that affect advice use. However, the extent and causes of suboptimal integration remain poorly understood. Quantifying the degree of optimality in this integration process is critical for uncovering the mechanisms driving suboptimal advice use because, even when all relevant information is transparently available, it must be integrated optimally to maximize decision accuracy. Our study focuses on purely cognitive mechanisms underlying suboptimal integration of advice-related information, independently of the social factors that typically shape human–human interactions. Specifically, our paradigm abstracts away from adviser intentionality^34,35^, trust formation^36^, normative influence^37–40^, social learning processes^12,41^, and social framing effects^42^.

The present study addresses these gaps in two ways. First, we systematically manipulate the relative differences between the cognitive and metacognitive skills of the participant (decision-maker) and those of a set of heterogeneous artificial advisers. Second, we employ Bayesian modeling to derive the best possible decision on a trial-by-trial basis, given all the information available to participants, i.e., global attributes of the self and the adviser, as well as local signals such as judgments and confidence from both. This approach establishes a principled benchmark for evaluating advice integration and provides a foundation for isolating its limitations. We consider several candidate sources of suboptimality:

- **Information insufficiency**: Decision-makers are capable of optimal integration in principle but lack access to critical information needed to maximize gains.
- **Integration error – selection**: All relevant information is available, but individuals neglect certain dimensions when making decisions.
- **Integration error – combination**: All relevant information is available and considered, but it is not combined optimally.
- **Integration error – adaptation**: Decision-makers do not adjust their advice use to specific adviser characteristics; instead, they apply generalized heuristics that ignore differences among advisers.
- **Individual differences**: Suboptimality is not uniform across the population but concentrated in a subset of decision-makers who consistently underperform across contexts.

Beyond clarifying the origins of imperfect advice integration, our work also aims to advance the applied study of assisted decision-making. Most existing work evaluates the benefits of advice through two empirical benchmarks: (1) whether accuracy improves relative to an individual’s initial judgment, and (2) whether final accuracy exceeds that of any single input (self or adviser). By contrast, our method introduces a third benchmark: (3) whether observed decisions approximate the best achievable outcome given all available information. This additional benchmark enables not only evaluating observed outcomes but also quantifying the gap between actual and achievable performance. Such insights are essential for assessing the potential gains of interventions such as training programs or ad hoc decision-support systems.

We designed two preregistered experiments employing a judge–adviser paradigm, in which participants engaged with seven artificial advisers, each differing in specific behavioral characteristics. Participants received information about advisers’ judgments and confidence, as well as unambiguous trial-by-trial feedback on outcomes (Experiment 1). This feedback allowed participants to learn the advisers’ global *cognitive* (i.e., accuracy) and *metacognitive* skills. The latter includes *confidence calibration*, defined as the mean deviation from actual and estimated accuracy, and *metacognitive sensitivity*, defined in this study as the trial-by-trial point-biserial correlation between confidence and accuracy. In the metacognitive literature, metacognitive sensitivity is often operationalized using various model-based and correlation-based measures^43–47^ and is also referred to as resolution^48^. In Experiment 2, participants could also rely on explicit descriptions of each adviser’s characteristics and of the behavioral attributes they themselves displayed in previous stages of the experiment. We employed Bayesian modeling to estimate the optimal use of both local informational signals (judgment, confidence) and global attributes of participants and advisers for each decision. It serves as a normative benchmark for optimal information integration and belief updating, rather than as a model intended to fit participants’ behavior. Unlike previous Bayesian approaches used to define optimal performance^13,28,36,49^, our model computes optimal Bayesian revision on a trial-by-trial basis, conditioning optimality strictly on the information available at the time of decision in a task involving continuous judgments. By comparing observed behavior to this normative benchmark, we aim to clarify the degree to which human advice integration deviates from optimality and isolate the cognitive and behavioral mechanisms responsible for these deviations.

## Methods

### Ethics statement

The methods of both Experiment 1 and Experiment 2 were performed in accordance with relevant guidelines and regulations and approved by the Ethics Committee of the University of Milano-Bicocca, Psychology Department (protocol N. RM-2023-731). Participants provided written informed consent to take part in the study.

### Preregistration

The main research questions, hypotheses, methods, and analysis plans for Experiment 1 and Experiment 2 have been preregistered in two separate OSF preregistrations. In the Supplementary Information (“Deviations from preregistered methods and analyses”), we report any deviations from preregistered methods and analyses. The links to the two preregistrations are available at https://osf.io/5ekdb/.

### Experiment 1

#### Experimental tasks

Experiment 1 consists of three different experimental tasks:

#### 1. Individual decision task (IDT)

**Fig. 1:**
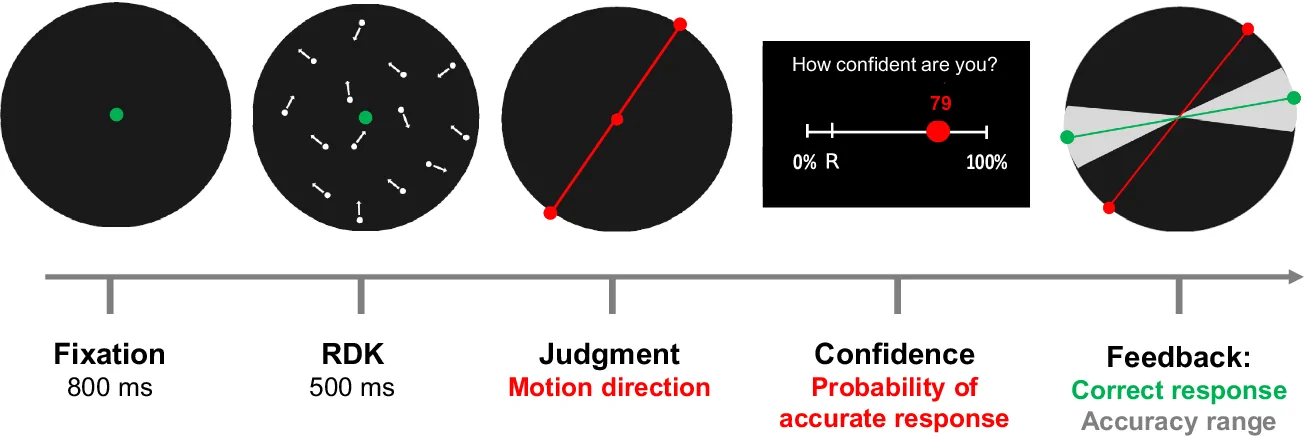
Individual Decision Task (IDT) procedure. In a typical IDT trial, the participant initially saw a green fixation point (Fixation). Then, the visual stimulus (RDK) appeared for 500 ms. After the stimulus had disappeared, the green point turned red, and the participant was asked to estimate the motion direction of the coherent dots, which was expressed by a red line (Judgment). Right after, the participant was asked to estimate the probability of having provided an accurate response (Confidence). The confidence scale ranged from 0% to 100% and highlighted, with a white tick and the letter R, the confidence level expected for a random response. Eventually, the participant was provided with feedback with the correct answer (green line) and the area representing the accuracy range (gray area).

The IDT served both as initial training and as a baseline task to estimate relevant parameters of participants’ performance. Participants estimated the direction of motion of stimuli (Random Dot Kinematograms, RDKs) displayed on the screen for 500 ms, then reported their confidence in their judgment (Fig. 1). Initially, the visual stimulus consisted of a black circular area on the screen, with a green fixation point at the center. After 800 ms, the RDK appeared and stayed on the screen for 500 ms. The RDK displayed ~55 dots per frame, every 4 pixels in diameter, moving at an angular velocity of 6°/s. In the RDK, a variable percentage of dots (the coherent dots) moved in the same direction within the circular area, whereas the remaining dots disappeared and appeared randomly on the screen. The % of points with coherent movement (motion coherence) varied across trials, leading to variability in participants’ sensory uncertainty. Right after the stimulus disappeared, the fixation point turned red. At this moment, the participant made her decision by clicking the left mouse button at a point within the black circular area to indicate the perceived direction of motion of the coherent dots. Her response was represented by a red straight line inside the black circular area. After the perceptual judgment, the participant was asked to indicate her confidence. In particular, she was asked to estimate her probability of having provided an accurate answer. Participants were instructed that an accurate answer consisted of a response that fell within an angle of 20° (10° clockwise and 10° counterclockwise) with respect to the actual direction of the dots. This accuracy range was determined through pilot studies to achieve an optimal balance between perceptual difficulty and the distribution of accuracy both within and between participants. The confidence assessment was provided on a scale from 0% to 100%, where 0% indicated a definitely inaccurate response and 100% indicated a definitely accurate response. Participants were also informed that a random response (i.e., the participant had not perceived any coherent motion direction) corresponded to a 11% probability of providing an accurate response. This value was visually ticked on the confidence scale as a visual reference for the participants (Fig. 1).

After the confidence assessment, the screen with the black circular area reappeared, and participants could view both the answer they provided (red line) and the correct stimulus direction (green line). Around the green line indicating the correct answer, participants were provided with a gray area representing the area where the judgment was considered accurate (Fig. 1). Therefore, in each trial, the participant received explicit feedback about the accuracy of her judgment.

Participants’ judgments and confidence assessments were monetarily incentivized (see section “Monetary incentives”).

#### Performance assessment in the IDT

Participants’ data in the IDT were used in two different ways: (1) as input for the creation of artificial agents in the following Advised Decision Task (ADT, see the next section); (2) to adjust the difficulty of the task (i.e., the dots’ coherence levels) so that all participants would reach a minimal performance in the ADT. Regarding the latter point, low perceptual sensitivity and a fixed dot coherence level would have resulted in null discriminability of motion direction, yielding random responses throughout the experiment.

Therefore, in the IDT, participants performed three blocks of 24 trials. The first block started with a fixed set of coherence levels (30%, 40%, 50%). At the end of the first block, we computed participants’ accuracy (i.e., the proportion of accurate judgments) and increased coherence levels by 5% if participants’ average accuracy was below 1/3, resulting in coherence levels of 35%, 45%, and 55%. The same procedure was applied at the end of the second block, leading to maximum coherence levels of 40%, 50%, 60% in the third block. The set of coherence levels chosen for the third block was used for the following task (ADT).

To estimate the participant’s performance for creating artificial agents in the ADT, we used the second and third blocks of the IDT. The first block was not considered to avoid potential learning effects. If the dot coherence level was changed at the end of the second block (i.e., if the coherence levels of the third block were different from those of the previous blocks), only the third block was considered for the performance assessment. In this way, artificial agents’ behaviors were generated solely from trials with the same coherence levels as those encountered by participants in the IDT.

#### 2. Advised decision task (ADT)

**Fig. 2:**
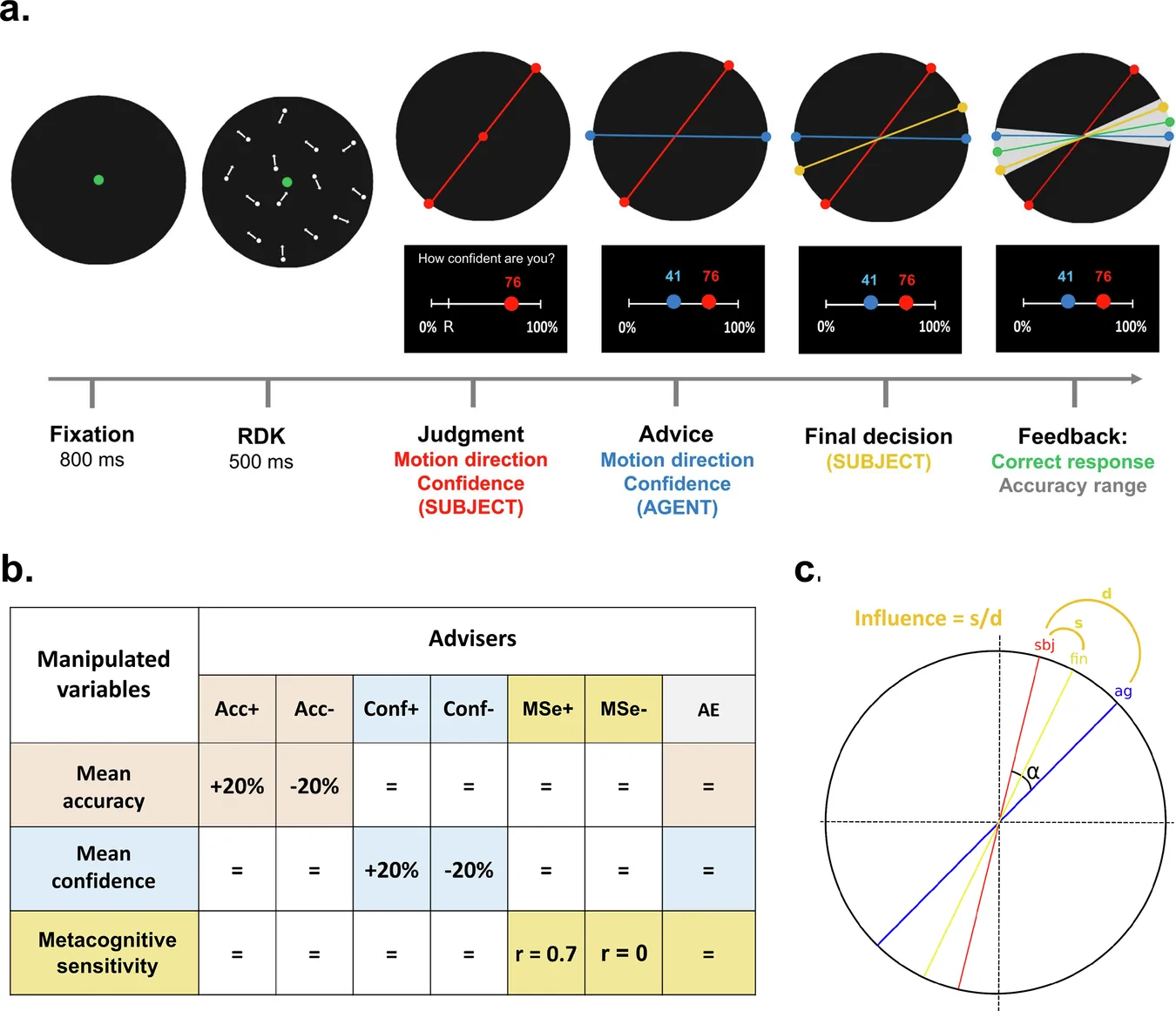
Experimental task and design. **a** Advised Decision Task (ADT). Initially, the participant saw a green fixation point (Fixation). Then, the visual stimulus (RDK) appeared for 500 ms (Perceptual stimulus). After the stimulus had disappeared, the participant was asked to estimate the motion direction of the coherent dots, which was expressed by a red line (Judgment). Right after, the participant was asked to estimate the probability of having provided an accurate response (Confidence). The confidence scale ranged from 0% to 100% and highlighted, with a white tick and the letter R (random), the confidence level expected for a random response. After the participant’s judgment and confidence assessment, we provided the agent’s advice (Advice): the judgment (blue line within the black circular area) and its confidence (blue dot at the bottom of the confidence scale). The agent’s judgment and confidence were shown alongside the participant’s (red line and red dot) to facilitate relative comparisons. Subsequently, participants had to make a final decision, represented by a yellow line (Final decision). Eventually, we provided accuracy feedback showing the correct answer (green line) and the area representing the accuracy range (gray area). **b** Artificial agents serving as advisers in the ADT. Agents Acc+, Acc-, Conf+, and Conf- were manipulated based on the participant’s parameters (mean accuracy, mean confidence, and metacognitive sensitivity). Acc+ had 20% accuracy *more* than the participant, Conf- 20% mean confidence *less* than the participant, and so on. The other two variables were set equal to the participant’s values. MSe+ and MSe- had fixed levels of correlation between trial-by-trial confidence and accuracy (r_pB_ ≈ 0.7 and r_pB_ ≈ 0, respectively), while mean accuracy and confidence were equal to the participants’ ones. Finally, the AE (Alter Ego) agent showed the same behavior as the participants across all three parameters. **c** Influence. Our main variable of interest is *Influence*, calculated as the shift from the participant’s initial to final decision (s), divided by the distance between the participant’s and the agent’s judgments (d). Influence expresses the weight assigned to the agent’s opinion, from 0 (no influence) to 1 (complete trust in the agent’s opinion).

The Advised Decision Task (ADT) was an interactive version of the IDT. In the ADT (Fig. 2a), the participant interacted with seven virtual agents (Fig. 2b) across seven experimental blocks, divided into two experimental sessions (see the “Experimental procedure” section). Participants were informed that they would interact with multiple artificial agents differing in behavior and characterized by stable performance.

The first part of each trial was identical to the IDT: the participant saw an RDK, made her judgment, and expressed her confidence in the judgment. The participant saw RDKs of three different dot coherence levels based on her performance in the IDT (see the previous section, “Performance assessment in the IDT”). The main difference was that in the ADT, a virtual agent performed the same task alongside the participant: the agent “saw” the same RDK, made a perceptual judgment, and expressed its confidence. After the participant’s confidence assessment, the screen with the black circular area reappeared. Within the area, the participant’s response (red line) and the virtual agent’s response were shown. Also, below the black circular area, participants received visual feedback on their own confidence (red shape) and the virtual agent’s confidence (blue shape). Then, the participant had to make a final decision regarding the direction of the coherent dots. This choice was represented with a straight yellow line. The relative adjustment of the participant’s final decision toward the adviser’s estimate was used as a measure of *influence* of the agent’s advice (Fig. 2c. See the “Main variables” section for details). As in the IDT, feedback on the correct response and the accuracy area was provided following the participant’s choice. The participant could decide when to skip to the subsequent trial by clicking the mouse. This feedback screen was displayed for a minimum of 1 s and a maximum of 5 s to balance the processing of relevant feedback and the necessity to perform many trials consecutively.

Participants performed 54 trials in each block, then moved to the next block, where they interacted with a different virtual agent. In Experiment 1, participants learned about the adviser’s behavioral attributes through trial-by-trial feedback and were not explicitly informed about them before the experimental block began. Participants were monetarily incentivized based on their initial judgments, their final responses, and their metacognitive sensitivity (see the “Monetary incentives” section).

Participants were informed that they would interact with multiple advisers differing in behavior and characterized by stable performance. We deliberately avoided framing the advisers in terms of intentionality, communicative goals, or strategic behavior. Our aim was to evaluate participants’ ability to use feedback and explicit performance-related knowledge to adapt their advice-taking behavior to a given source, and to assess whether and how suboptimality emerges despite sufficient information for optimal integration.

At the end of each of the seven blocks, participants were asked several questions:

1. Think back to YOUR initial answers (red answers) in the block you just faced. How much do you think was YOUR percentage of accurate initial responses?
2. Think back to the agent’s initial responses (blue responses) in the block you just faced. How much do you think was ITS percentage of accurate initial responses?
3. Think back to YOUR confidence ratings in the block you just faced. How much do you think was the average value of YOUR confidence judgments?
4. Think back to the agent’s confidence ratings in the block you just faced. How much do you think was the average value of ITS confidence judgments?
5. Think back to all the trials in which YOU have provided an ACCURATE initial judgment. How much was the average value of YOUR confidence judgments in these trials?
6. Think back to all the trials in which YOU have provided an INACCURATE initial judgment. How much was the average value of YOUR confidence judgments in these trials?
7. Think back to all the trials in which the AGENT has provided an ACCURATE initial judgment. How much was the average value of ITS confidence judgments in these trials?
8. Think back to all the trials in which the AGENT has provided an INACCURATE initial judgment. How much was the average value of ITS confidence judgments in these trials?

Questions 1 and 2 were meant to disclose the participant’s explicit assessment of the agent’s and their own overall accuracy.

Questions 3 and 4 were meant to disclose the participant’s explicit assessment of the agent’s and their own mean confidence.

Questions 5 and 6 aimed to evaluate the participant’s explicit assessment of her own metacognitive sensitivity.

Questions 7 and 8 aimed to evaluate the participant’s explicit assessment of the agent’s metacognitive sensitivity.

### Experimental procedure

Participants underwent two different lab experimental sessions. In the two sessions, the temporal succession of events consisted of:

Session 1:

1. Instructions IDT
2. Training IDT (12 trials)
3. IDT (3 blocks of 24 trials)
4. Instructions ADT
5. ADT (3 or 4 blocks of 54 trials)

Session 2:

1. IDT (task re-familiarization, 12 trials)
2. ADT (3 or 4 blocks of 54 trials, depending on the number of blocks faced in Session 1)

Each participant randomly faced 3 or 4 blocks of the ADT in the first experimental session. In each block, the participant interacted with a different virtual agent. In each block, a virtual agent among the seven was randomly selected. As a constraint, agents manipulated by the same variable (e.g., mean accuracy) were always drawn in the same experimental session (see next paragraph, “Designing agents’ behavior” for the definition of the seven virtual agents). Consequently, the only agent that was not manipulated on any variable (Agent “Alter Ego,” AE) was drawn in Session 1 if the participant faced three blocks in that session, whereas it was drawn in Session 2 if Session 1 consisted of four blocks. In this way, all the virtual agents had the same probability of being drawn in Session 1 and Session 2. See the next paragraph for the definition of the different virtual agents.

The software used for stimulus presentation was Psychtoolbox for Windows version 3.0.16, a toolbox running under Matlab version R2020a. Testing was carried out in person.

### Designing agents’ behavior

In our experimental setup, participants interacted with seven agents during the ADT. Agents’ behavior was manipulated along three dimensions:

1. **Agent mean accuracy (aMA):** The agent’s proportion of accurate judgments along the 54 trials of the experimental block.
2. **Agent mean confidence (aMC):** The mean value of the confidence ratings of the agent along the 54 trials of the experimental block.
3. **Agent metacognitive sensitivity (aMS)**: The correlation between the trial-by-trial agents’ confidence ratings and accuracy.

In particular, each of the seven agents was manipulated based on the data of the participant with whom it was interacting in the current block. The aim was to obtain agents’ parameters with specific relationships to the participant’s parameters.

The first two agents were manipulated based on their mean accuracy. The first agent (Acc+) was set to show higher accuracy (+20%) than the participant, whereas the second agent (Acc–) showed lower accuracy (–20%). This meant that, if the participant showed an accuracy of 0.5, Acc+ showed an accuracy of 0.7, and Acc− showed an accuracy of 0.3. For both agents, the mean confidence and reliability values were set to match the participant’s values to obtain agents that differed in only one parameter (mean accuracy).

The third and fourth agents were manipulated based on their mean confidence. One agent (Conf+) was set to show a higher mean confidence (+20%) than the participant, whereas the other agent (Conf–) showed a lower mean confidence (–20%) than the participant. For both agents, the mean accuracy and metacognitive sensitivity values were set to match the participant’s values.

The fifth and sixth agents were manipulated based on their metacognitive sensitivity. One agent (MSe+) was set to show a high positive correlation between confidence and accuracy (point-biserial correlation: r_pB_ = 0.7) and a high negative correlation between confidence and absolute angular error (angular distance with correct motion direction, Pearson’s correlation: r = −0.8). The other agent (MSe–) was set to show null correlation between confidence and accuracy (r_pB_ = 0) and between confidence and absolute angular error (r = 0). For both agents, the mean accuracy and mean confidence values were set to match the participant’s values. We used fixed correlation coefficients for MSe+ and MSe− agents, with a marked difference between them, to ensure discriminability in their metacognitive sensitivity. We assumed that correlations were more difficult to discriminate than averages; indeed, two sets of vectors with different correlations required a large discrepancy in correlation coefficients to be noticed. To assess the robustness of our metacognitive sensitivity measure, we conducted additional analyses (SI, “Alternative measures of metacognitive ability”) using alternative measures of metacognitive sensitivity (meta-*I* and meta-${I}_{2}^{r}$^43^).

The last agent (AE: Alter Ego) showed the exact parameters of the participants. Therefore, the mean accuracy, mean confidence, and metacognitive sensitivity were set equal to the participant’s values.

### Estimating participants’ behavioral parameters

At the end of the Individual Decision Task (IDT), we computed three behavioral parameters for each participant: mean accuracy, mean confidence, and metacognitive sensitivity. These parameters were calculated from the last two blocks of the IDT for participants who maintained the same level of motion coherence in these two blocks. Only data from the last block were considered for participants who faced an increase in motion coherence in the last block of the IDT. This was intended to estimate participants’ parameters based on the level of motion coherence they would encounter in the advised decision task (ADT). The first block of the IDT was always discarded to control for initial learning effects arising from task novelty.

This dataset was used to estimate the parameters of the agent’s behavior randomly assigned the first block. From the second block onward and throughout the entire experiment, this set of data was updated to include the ADT’s decisions. Therefore, at the beginning of every block of the ADT, the participant’s parameters of mean accuracy, mean confidence, and metacognitive sensitivity were re-estimated based on the previous blocks of the ADT and the relevant blocks of the IDT. For blocks falling in the second experimental session, data from the IDT were not considered.

Then, in each ADT block, these values were used as inputs to the algorithm that generated the virtual agent’s behavior, randomly selected for the current block. After applying the parameter modulation based on the current agent type, the algorithm used the agents’ parameters to generate perceptual judgments and confidence ratings (Full details about the process of generation of agent’s behavior can be found in the Supplementary Methods).

## Sampling and data collection

### Recruiting and participation criteria

Participants were recruited through the University of Milano-Bicocca’s mailing lists and dedicated recruiting platforms (Sona; Orsee). These systems allowed the experiment to be advertised to a list of voluntary students from different departments of the University. However, participation was not restricted to students of the University of Milano-Bicocca. Participants were required to have normal-to-corrected vision, no neurological disorders, and knowledge of the Italian language to ensure comprehension of the experimental instructions.

Data were collected from 96 participants (50 in Experiment 1, 46 in Experiment 2). In Experiment 1, three participants completed only the first experimental session and did not attend the second session, and were therefore excluded from the analysis (see exclusion criteria in the section “Data exclusion”). One participant was excluded for sub-threshold accuracy (initial accuracy: 0.14; final accuracy: 0.13). In Experiment 2, two participants completed only the first session and were excluded, and one participant was excluded for sub-threshold accuracy (initial accuracy: 0.11; final accuracy: 0.11). Thus, the final analysis included 89 participants (46 in Experiment 1, 43 in Experiment 2). In Experiment 1, the sample comprised 20 males and 26 females (mean age = 23.09, SD = 6.76), whereas in Experiment 2, the sample comprised 19 males and 24 females (mean age = 25.28, SD = 8.90). Gender, along with age, was self-reported by participants during the initial demographic screening. We did not collect data on SES, communities of descent, or ethnicity.

### Monetary incentives

We reimbursed participants based on their actual performance in the task. This aimed to incentivize participants to make decisions as accurately as possible.

In the IDT, the participant’s score was determined based on the following parameters:

1. Accuracy of perceptual judgments. The score is a linear function of the proportion of accurate perceptual judgments, converted into € in the range [0,1]. We set a minimum performance bound below which the participant receives the minimum score, and a maximum performance bound above which the participant receives the maximum score. These values are intended to yield scores and reimbursements in line with the predicted distribution of performance instances in the participants’ pool. The choice of the exact performance bounds is based on the results of a pilot study. Specifically, with this method, we expect participants with an average score to obtain an average reimbursement (i.e., 0.5 € for the current parameter) and participants with particularly low or high scores to obtain the minimum and maximum reimbursement, respectively. We set the following limits for the accuracy score: Min. accuracy = 0.10; Max. accuracy = 0.70.
2. Metacognitive sensitivity. Point-biserial correlation between accuracy and confidence, converted in € in the range [0,1] (Min. correlation = 0; Max. correlation = 0.50). A basic definition and interpretation of the point-biserial correlation between confidence and accuracy (i.e., high correlation: high confidence for accurate judgments and low confidence for inaccurate ones) was provided in the instructions.
3. In the ADT, the participant’s score was determined based on the following parameters:
  1. Accuracy of perceptual judgments. The score is a linear function of the proportion of accurate perceptual judgments, converted into € in the range [0, 5] (Min. accuracy = 0.10; Max. accuracy = 0.70).
  2. Accuracy of final decision: The score is a linear function of the proportion of accurate final decisions, converted in € in the range [0, 5] (Min. accuracy = 0.20; Max. accuracy = 0.80).
  3. Metacognitive sensitivity: Point-biserial correlation between accuracy and confidence, converted in € in the range [0,5] (Min. correlation = 0; Max. correlation = 0.50).
  4. Ratings: Absolute distance from the correct response to the actual rating response (rating error) for each question. Since all the questions require estimates in %, we compute the average rating error (mean rating error). The obtained mean rating error is linearly converted into € in the range [0,1] (Min. rating error = 0%; Max. rating error = 50%).

## Data analysis

### Main variables

Measured variables and factors for primary analyses:

- Judgment accuracy: binary variable defining if the current perceptual judgment (provided by the participant or the agent) has been classified as accurate (1) or inaccurate (0). An accurate response is defined as a judgment falling within an angle of 20° (10° clockwise and 10° counterclockwise) with respect to the actual motion direction.
- Confidence: the confidence (i.e., the estimated probability of an accurate response in the correct trial) expressed by the participant or the agent.
- Final decision accuracy: binary variable defining if the current participant’s final decision has been classified as accurate (1) or inaccurate (0). An accurate response is defined as a final decision falling within an angle of 20° (10° clockwise and 10° counterclockwise) with respect to the actual motion direction.

Derived variables for primary analyses:

- Δ confidence normalized: We considered the agent’s and the participant’s trial-by-trial confidence judgments as proportions (confidence/100). Then, for both the participants and the agent, we subtracted from each confidence judgment the average participant’s (or agent’s) confidence to normalize for mean confidence. Eventually, we computed the difference between the two normalized confidence judgments (agent - participant) to express the trial-by-trial relative difference in confidence.
- Δ mean accuracy: We computed the difference between the agent’s and participant’s mean accuracy. Consistent with the computations of our Bayesian model (see “Bayesian modeling of optimal behavior”), participants’ mean accuracy was continuously updated across the entire experiment, while agent-related accuracies were updated within each block. In Experiment 2, the agent’s mean accuracy was not updated trial by trial but instead remained fixed across the trials of a block, because the value disclosed at the beginning of the block accurately reflected the agent’s aggregate performance. See the variable distribution in Supplementary Fig. S1.
- Δ mean confidence: We computed the difference between the agent’s and participant’s mean confidence, using the same approach as Δ mean accuracy. See the variable distribution in Supplementary Fig. S1.
- Δ metacognitive sensitivity: We computed the difference between the agent’s metacognitive sensitivity (point-biserial correlation between trial-by-trial confidence and accuracy) and the participant’s metacognitive sensitivity, using the same approach of Δ mean accuracy. See the variable distribution in Supplementary Fig. S1.
- Participant’s normalized confidence: We considered the participant’s trial-by-trial confidence judgments, and we subtracted from each confidence judgment the average participant’s confidence to normalize for mean confidence. The resulting value was converted to a proportion (/100).
- Agent’s normalized confidence: We considered the agent’s trial-by-trial confidence judgments, and we subtracted from each confidence judgment the average agent’s confidence to normalize for mean confidence. The resulting value was converted to a proportion (/100).
- Individual miscalibration (overconfidence): We computed the averages of the participants’ trial-by-trial confidence and judgment accuracy. We subtracted the participants’ average accuracy from the average confidence, returning an individual measure of confidence (mis)calibration. Positive values indicate *overconfidence*, negative values indicate underconfidence.
- Influence: We computed a trial-by-trial measure of the agent’s *influence* on participants’ final decision in the current trial (Fig. 2c). This index was calculated as the adjustment toward the agent in the participant’s final decision, divided by the distance between the participant’s and the agent’s responses. We always considered the minimum angle between the participant’s and the agent’s responses (Fig. 2c). If the participants’ final decision falls outside α, it was discarded. However, we considered a tolerance angle γ (10°): if the participant’s final decision was outside α but within γ, the decision was considered equivalent to an influence of 0 (closer to the participant’s initial estimate) or 1 (closer to the agent’s estimate). We chose this threshold because it was consistent with the accuracy range. Moreover, trials in which α was smaller than 5° were not included in the computation of the influence variable, since the two initial responses would likely appear nearly equivalent from the participant’s perspective (see the “Control analyses” section).
- Optimality: In each trial, we calculated the absolute distance between actual and observed influence as a measure of distance from optimality (dO). Then we computed 1-dO to obtain a positive index of *optimality* in advice integration.

### Statistical analyses

Statistical analyses were performed mainly using mixed-effects models. Most models have been replicated across both Experiment 1 and Experiment 2. Full results are available in the Supplementary Information. The data met the assumptions of the statistical tests used. Here we list and describe all the mixed-effects models used in the analysis:

- **Model 1.1 (Exp1) and Model 2.1 (Exp2):**

These models aimed to compare participants’ final accuracy with their initial judgment accuracy and the agents’ initial judgment accuracy to investigate accuracy enhancement after advice. Accuracy (*A*) was used as the dependent variable. A random effect (*u*_*sbj*_) was applied to the intercept at the participant level to control for within-participant outcome dependencies. The model included the following predictors:

- decision type (participant, adviser, final) (DT)
- adviser type (initial, final) (AT)
- decision type*adviser type (DT*AT)

Full model: *A* = *β*_*0*_ + *β*_*1*_*DT* + *β*_*2*_*AT* + *β*_*3*_DT*AT + u_*sbj*_ + *ε*

- **Model 1.2 (Exp1) and Model 2.2 (Exp2):**

These models aimed to compare participants’ final accuracy to that of our Bayesian model of optimal advice integration, to investigate the effect of participants’ suboptimality. Accuracy (*A*) was used as the dependent variable. A random effect (*u*_*sbj*_) was applied to the intercept at the participant level to control for within-participant outcome dependencies. The model included the following predictors:

- decision type (final, optimal) (DT)
- adviser type (initial, final) (AT)
- decision type*adviser type (DT*AT)

Full model: *A* = *β*_*0*_ + *β*_*1*_*DT* + *β*_*2*_*AT* + *β*_*3*_DT*AT + u_*sbj*_ + *ε*

- **Model 1.3 (Exp1) and Model 2.3 (Exp2):**

These models tested whether participants adapted to the relative difference between the adviser’s and their own individual traits and informational signals (accuracy, mean confidence, metacognitive sensitivity, and trial-by-trial confidence). Influence (*I*) was used as the dependent variable. A random effect (*u*_*sbj*_) was applied to the intercept at the participant level. Model 1.3 was run, discarding the first third of the trials in each block, assuming that participants needed some trials to estimate the adviser’s attributes. For model 2.3, all data were used since the adviser’s attributes were fully disclosed at the beginning of the block.

The model included the following predictors:

- Δ mean accuracy (*A*)
- Δ mean confidence (M*C*)
- Δ metacognitive sensitivity (M*S*)
- Δ confidence normalized (C)
- Δ metacognitive sensitivity* Δ confidence normalized (MS*C)

Full model: *I* = *β*_*0*_ + *β*_*1*_*A* + *β*_*2*_*MC* + *β*_*3*_*MS* + *β*_*4*_*C* + *β*_*5*_MS*C+u_*sbj*_ + *ε*

- **Model 1.4 (Exp1) and Model 2.4 (Exp2):**

Models 1.4 and 2.4 compared the beta coefficients for the predictors of observed influence (Models 1.3 and 2.3) with those expressing the weights of the predictors of *optimal influence*. To do so, we ran the same models (1.3 and 2.3) and added a dummy variable called *influence type* (0: optimal influence; 1: observed influence). We considered the main effect of influence type and all interactions between influence type and the predictors in Models 1.3 and 2.3. A random effect (*u*_*sbj*_) was applied to the participant-level intercept. Moreover, a random slope (*u*_*it*_) was applied to *influence type*, assuming inter-individual variability in the optimality of advice use. Model 1.4 was run, discarding the first third of the trials in each block, while for model 2.4, all data were used.

The model included the following predictors:

- Δ mean accuracy (*A*)
- Δ mean confidence (M*C*)
- Δ metacognitive sensitivity (*MS*)
- Δ confidence normalized (C)
- Δ metacognitive sensitivity* Δ confidence normalized (MS*C)
- influence type (IT)
- Δ mean accuracy*influence type (A*IT)
- Δ mean confidence*influence type (MC*IT)
- Δ metacognitive sensitivity*influence type (MS*IT)
- Δ confidence normalized*influence type (C*IT)
- Δ metacognitive sensitivity* Δ confidence normalized*influence type (C*MS*IT)

Full model: *I* = *β*_*0*_ + *β*_*1*_*A* + *β*_*2*_*MC* + *β*_*3*_*MS* + *β*_*4*_*C* + *β*_*5*_MS*C + *β*_*6*_*IT* + *β*_*7*_A*IT + *β*_*8*_MC*IT + *β*_*9*_MS*IT + *β*_*10*_C*IT + *β*_*11*_C* MS*IT + u_*sbj*_ *+ u*_*it*_ + *ε*

- **Model 1.4b (Exp1) and Model 2.4b (Exp2):**
  Models 1.4b and 2.4b tested whether the overreaction to non-predictive mean confidence stemmed from an insufficient rescaling of miscalibrated confidence. To test this hypothesis, we ran a variant of Model 1.4 (and 2.4) in which the Δ trial-by-trial confidence [agent–subject] was not normalized by the adviser’s and participant’s mean confidence (Cn). In this formulation, the estimate for the Δ mean confidence regressor reflects the extent to which participants rescaled confidence signals based on overall confidence calibration. All other features of the models were identical to those of Model 1.4 (and 2.4).
  Full model: *I* = *β*_*0*_ + *β*_*1*_*A* + *β*_*2*_*MC* + *β*_*3*_*MS* + *β*_*4*_*Cn* + *β*_*5*_MS*Cn + *β*_*6*_*IT* + *β*_*7*_A*IT + *β*_*8*_MC*IT + *β*_*9*_MS*IT + *β*_*10*_Cn*IT + *β*_*11*_Cn* MS*IT + u_*sbj*_ *+ u*_*it*_ + *ε*
- **Model 1.4c (Exp1) and Model 2.4c (Exp2):**
  Models 1.4c and 2.4c tested whether the insufficient modulation of influence by Δ confidence was primarily driven by a selective underweighting of the adviser’s, rather than the participant’s, confidence signal. To test this, we ran another variant of Model 1.4 (and 2.4) in which the normalized Δ confidence [agent–subject] regressor was replaced with two separate regressors: self (normalized) confidence (SC) and adviser (normalized) confidence (AC). This allowed us to isolate the effect of each confidence signal on influence. All other features of the models were identical to those of Model 1.4.
  Full model: *I* = *β*_*0*_ + *β*_*1*_*A* + *β*_*2*_*MC* + *β*_*3*_*MS* + *β*_*4*_SC + *β*_*5*_*AC* + *β*_*6*_MS*SC + *β*_*7*_MS*AC + *β*_*8*_*IT* + *β*_*9*_A*IT + *β*_*10*_MC*IT + *β*_*11*_MS*IT + *β*_*12*_SC*IT + *β*_*13*_AC*IT + *β*_*14*_SC*MS*IT + *β*_*15*_AC* MS*IT + u_*sbj*_ *+ u*_*it*_ + *ε*
- **Model 1.5, Model 1.7, and Model 1.9 (Exp1):**
  These three models aimed to test the relationship between estimated and actual attributes of the participant and the adviser in Experiment 1. Estimated attributes (for both self and adviser attributes) were collected at the end of each block (see “Participants’ estimation of their own and agents’ attributes”). The dependent variables were participants’ block-by-block estimates of the self’s and the adviser’s accuracy (eA, Model 1.5), mean confidence (eMC, Model 1.7), and metacognitive sensitivity (eMS, Model 1.9). The metacognitive sensitivity estimate was defined as the difference between the estimated mean confidence for accurate and inaccurate (initial) judgments. The independent variable consisted of the actual self and adviser’s attributes (for each block): actual adviser’s accuracy (aA, Model 1.5), mean confidence (aMC, Model 1.7), and metacognitive sensitivity (aMS, Model 1.9). In all three models, we added a dummy variable for identity (self vs. adviser) and an interaction with the actual attribute. In all three models, a random effect (*u*_*sbj*_) was applied to the intercept at the participant level to control for dependency of within-participant outcomes.
  Model 1.5: *eA* = *β*_*0*_ + *β*_*1*_*aA* + *β*_*2*_*ID* + *β*_*3*_aA*ID+ u_*sbj*_ + *ε*
  Model 1.7: *eMC* = *β*_*0*_ + *β*_*1*_*aMC* + *β*_*2*_*ID* + *β*_*3*_aMC*ID + u_*sbj*_ + *ε*
  Model 1.9: *eMS* = *β*_*0*_ + *β*_*1*_*aMS* + *β*_*2*_*ID* + *β*_*3*_aMS*ID + u_*sbj*_ + *ε*
- **Model 1.6, Model 1.8, and Model 1.10 (Exp1):**
  These three models aimed to test the relationship between self and adviser inter-block estimates by adviser type (see Models 1.5, 1.7, 1.9). The dependent variables were the participants’ block-by-block estimates of the self’s and the adviser’s accuracy (eA, Model 1.6), mean confidence (eMC, Model 1.8), and metacognitive sensitivity (eMS, Model 1.10). The metacognitive sensitivity estimate was defined as the difference between the estimated mean confidence for accurate and inaccurate (initial) judgments. We used two dummy variables and their interaction as predictors of interest: identity (*ID*, self vs. adviser) and adviser type (ADV). In all three models, a random effect (*u*_*sbj*_) was applied to the intercept at the participant level to control for dependency of within-participant outcomes.
  Model 1.6: *eA* = *β*_*0*_ + *β*_*1*_*ID* + *β*_*2*_*ADV* + *β*_*3*_ID*ADV + u_*sbj*_ + *ε*
  Model 1.8: *eMC* = *β*_*0*_ + *β*_*1*_*ID* + *β*_*2*_*ADV* + *β*_*3*_ID*ADV + u_*sbj*_ + *ε*
  Model 1.10: *eMS* = *β*_*0*_ + *β*_*1*_*ID* + *β*_*2*_*ADV* + *β*_*3*_ID*ADV + u_*sbj*_ + *ε*
- **Model 3.1**

Model 3.1 aimed to test whether optimality in advice integration was enhanced when full information on the relative competencies of the decision-maker and the adviser was explicitly revealed beforehand (Exp2), compared to a situation in which this information should be learned through trial-by-trial feedback (Exp1).

To answer this question, we pooled data from Exp1 and Exp2. We used the dependent variable Optimality (O). The independent variables consisted of the informational signals used in Model 1.4 and 2.4. Moreover, we added a dummy variable for treatment (0: Exp1; 1: Exp2) to test for the effect of exposure to knowledge about the participant’s and agent’s competencies on optimality. We considered the main effect of treatment and all the interactions between treatment and the informational signals. A random effect (*u*_*sbj*_) was applied to the intercept at the participant level. Model 3.1 was run discarding the first third of the trials of Experiment 1 data. The model included the following predictors:

- Δ mean accuracy (*A*)
- Δ mean confidence (M*C*)
- Δ metacognitive sensitivity (*MS*)
- Δ confidence normalized (C)
- Δ metacognitive sensitivity* Δ confidence normalized (MS*C)
- treatment (T)
- Δ mean accuracy*treatment (A*T)
- Δ mean confidence*treatment (MC*T)
- Δ metacognitive sensitivity*treatment (MS*T)
- Δ confidence normalized*treatment (C*T)
- Δ metacognitive sensitivity* Δ confidence normalized*treatment (C*MS*T)

Full model: *O* = *β*_*0*_ + *β*_*1*_*A* + *β*_*2*_*MC* + *β*_*3*_*MS* + *β*_*4*_*C* + *β*_*5*_MS*C + *β*_*6*_*T* + *β*_*7*_A*T + *β*_*8*_MC*T + *β*_*9*_MS*T + *β*_*10*_C*T + *β*_*11*_C*MS*T + u_*sbj*_ + *ε*

- **Model 3.2:**
  Model 3.2 aimed to test whether different individual levels of confidence predict heterogeneity in suboptimal use of relevant informational signals and attributes. We ran a variant of Model 1.4 (and 2.4) that added a dummy variable for the metacognitive sensitivity group (MSG; 0: low MS; 1: high MS) and its interactions with the other predictors of interest. MSG was obtained by median-splitting individual metacognitive sensitivity scores. We pooled data from Exp1 and Exp2. We discarded the first third of the trials from Experiment 1. A random effect (*u*_*sbj*_) was applied to the intercept at the participant level.
- **Model 3.3:**
  Model 3.3 aimed to test whether different individual levels of metacognitive sensitivity predicted suboptimality in the use of relevant informational signals and attributes. We ran a variant of Model 3.2 in which we focused only on predictors that could shed light on the different uses of the self and adviser’s confidence signals. We added a dummy variable for metacognitive sensitivity group (MSG, 0: low MS; 1: high MS), one for influence type (IT, 0: optimal influence; 1: observed influence) and their interactions with the two main predictors of interest (self (normalized) confidence (SC) and adviser (normalized) confidence (AC)). MSG was obtained by median-splitting individual metacognitive sensitivity scores. We pooled data from Exp1 and Exp2. We discarded the first third of the trials from Experiment 1. A random effect (*u*_*sbj*_) was applied to the intercept at the participant level.
  Full model: *I* = *β*_*0*_ + *β*_*1*_MSG + *β*_*2*_*IT* + *β*_*3*_*SC* + *β*_*4*_*AC* + *β*_*5*_ MSG*IT + *β*_*6*_SC*IT + *β*_*7*_AC*IT + *β*_*8*_SC* MSG + *β*_*9*_AC*MSG + *β*_*10*_SC*IT* MSG + *β*_*11*_AC*IT* MSG *+ u*_*sbj*_ + *ε*
- **Model 3.4:**
  Model 3.4 aimed to test whether different individual levels of overconfidence predicted suboptimality in the use of relevant informational signals and attributes. We ran a variant of Model 1.4 (and 2.4) that added a dummy variable for the overconfidence group (OVG; 0: low overconfidence; 1: high overconfidence) and its interactions with the other predictors of interest. OVG was obtained through a median split of overconfidence (mean confidence − scores). We pooled data from Exp1 and Exp2. We discarded the first third of the trials from Experiment 1. A random effect (*u*_*sbj*_) was applied to the intercept at the participant level.
- **Model 3.5:**
  Model 3.5 aimed to test whether different individual levels of overconfidence predicted suboptimality in the use of relevant informational signals and attributes. We ran a variant of Model 3.4 in which we focused only on predictors that could shed light on the different uses of the self and adviser’s confidence signals. We added a dummy variable for overconfidence group (OVG, 0: low overconfidence; 1: high overconfidence), one for influence type (IT, 0: optimal influence; 1: observed influence), and their interactions with the two main predictors of interest (self (normalized) confidence (SC) and adviser (normalized) confidence (AC)). OVG was obtained through a median split of overconfidence (mean confidence − scores). We pooled data from Exp1 and Exp2. We discarded the first third of the trials from Experiment 1. A random effect (*u*_*sbj*_) was applied to the intercept at the participant level.
  Full model: *I* = *β*_*0*_ + *β*_*1*_*OVG* + *β*_*2*_*IT* + *β*_*3*_*SC* + *β*_*4*_*AC* + *β*_*5*_OVG*IT + *β*_*6*_SC*IT + *β*_*7*_AC*IT + *β*_*8*_SC*OVG + *β*_*9*_AC*OVG + *β*_*10*_SC*IT*OVG + *β*_*11*_AC*IT*OVG + u_*sbj*_ + *ε*
- **Regression models for inter-individual analysis**
  The section “Metacognitive and cognitive skills predict optimality in advice integration” included several additional linear and logistic mixed-effects and simple regressions, testing associations between two variables. When variables were considered trial-by-trial, we used mixed-effects models with a random intercept at the subject level. For continuous individual variables, we used linear regression. When relevant, we tested for differences across experiments by adding a dummy variable for experiment; otherwise, we ran the models separately for Exp1 and Exp2. We tested the following relationships:
  - trial-by-trial confidence and accuracy (separately for Exp1 and Exp2).
  - trial-by-trial dot coherence level and confidence (separately for Exp1 and Exp2).
  - trial-by-trial dot coherence level and accuracy (separately for Exp1 and Exp2).
  - trial-by-trial confidence and RTs of initial perceptual judgments (separately for Exp1 and Exp2).
  - trial-by-trial confidence and RTs of final decisions (separately for Exp1 and Exp2). At -
  - mean confidence and mean accuracy (by experiment).
  - individual metacognitive sensitivity and optimality (by experiment).
  - individual overconfidence and optimality (by experiment).

### Sample size and power estimation

The power analysis was based on Model 1.4 (Experiment 1) and Model 2.4 (Experiment 2), which aimed to test for optimality in advice integration. The R package SimR was employed to run simulations, leveraging data from a pilot study with 14 participants. The pilot data helped estimate realistic effect sizes and distribution parameters for the main study.

For each set of simulations (one for each experiment), the influence type predictor was chosen as the primary effect of interest. This predictor assessed how optimally participants used advice in their decision-making. The standardized beta coefficient for this predictor was set to 0.1, corresponding to an unstandardized beta of 0.03 in the pilot data. In practical terms, this represented a modest 3% difference between optimal and actual influence at the group level. Each participant was expected to contribute 36 observations to the analysis, derived from the total trials per block (54) minus the first third of the block, which was excluded to account for learning effects. Simulations also accounted for additional trial exclusions based on predefined criteria, ensuring a realistic estimate of the available data.

The power analysis aimed for a conventional significance level of *p* = 0.05 and a robust power of 0.9, meaning the study had a 90% chance of detecting the specified effect if it truly existed in the population. Multiple simulations were run with varying sample sizes to determine the minimum required sample size. Each simulation included 10,000 iterations to ensure stable, reliable results. The analysis indicated that a sample size of 40 participants (for each experiment) was the minimum required to achieve the desired power for detecting the specified effect. We also explored the impact of a slightly larger effect size and found that if the standardized beta coefficient for the influence type predictor were increased to 0.15 (corresponding to an unstandardized beta of 0.045), the study would have over 99.9% power with the same sample size of 40 participants.

### Sensitivity power analysis

We conducted a sensitivity power analysis based on the final sample size to evaluate whether the study was adequately powered to detect the smallest effect size of theoretical or pragmatic interest (SESOI) for our main preregistered hypotheses (Models 1.3, 1.4, 2.3, and 2.4). Power was estimated via Monte Carlo simulation (1000 iterations) in PAMLj (Jamovi) for mixed-effects models, using variance components from the final dataset. Because closely comparable prior effect sizes are not available for our paradigm and model-based predictors, we defined a conservative SESOI directly on the scale of our key interaction terms: B = 0.01, corresponding to an approximately 1% change in the relevant moderation (Δ *confidence* × Δ *metacognitive sensitivity*; and Δ *confidence* × Δ *metacognitive sensitivity* × *influence type*). Under this conservative SESOI, simulated power remained high (≈95–96%) for the main and interaction effects. For the between-subject associations between metacognitive indices and individual optimality, power exceeded 80% for standardized effects ≥0.25, which is smaller than all observed effects and commonly considered small in the literature. We therefore treated 0.25 as a conservative SESOI for these correlational analyses. Overall, the final sample size provided high sensitivity to detect effects at or below the SESOI for our main hypotheses.

## Data exclusion

### Exclusion of participants

- Participants who performed only the first experimental session and did not show up for the second session, or who did not complete the experiment.
- Initial and final mean accuracy in the ADT. We set a minimum mean accuracy of 0.15 for initial and final decisions in the ADT to include the participant’s data. We discarded data of participants who performed below this threshold in *both* the performance parameters (initial and final decisions). Indeed, we considered lower levels of accuracy for both parameters as a strong indicator of random behavior (chance level: 0.11).

### Exclusion of trials

*Influence* and *optimal influence* variables were not computed in these cases:

- Distance between the two initial responses <5°.
- Final/Optimal response outside the minimum angle (α) subtended by the two initial responses, with a tolerance of 10°.

### Bayesian modeling of optimal behavior

**Fig. 3:**
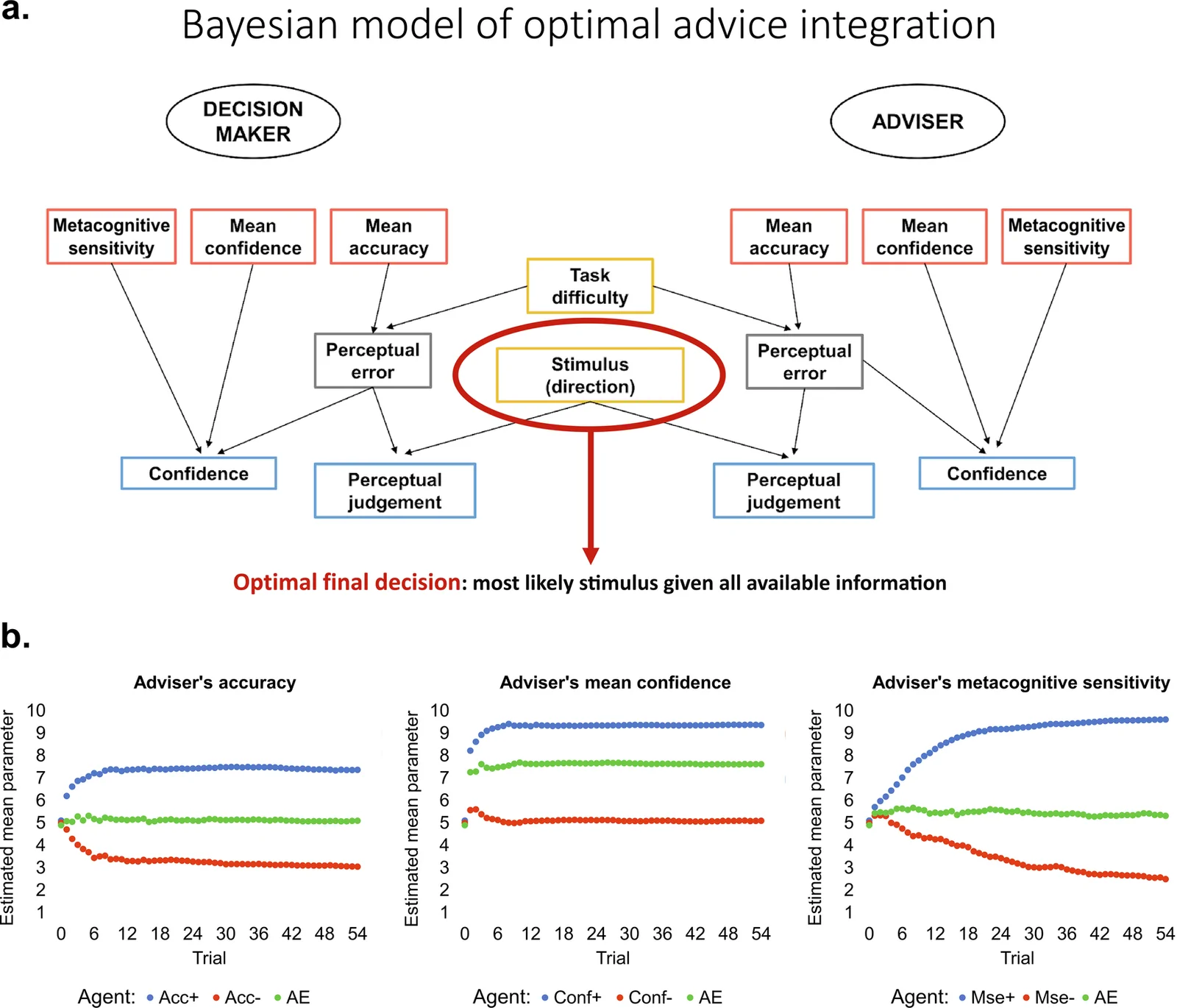
Bayesian model of optimal behavior. **a** Our Bayesian model consisted of a directed acyclic graph (DAG) where nodes (rectangles) expressed random variables, and arcs (arrows) represented causal relationships between those variables (expressed as directed probabilistic dependencies). In each trial, the network gathered definite evidence about local informational signals (blue squares: judgments and confidence evaluations) and conjectures about global attributes (red squares: probability distributions of accuracy, mean confidence, and metacognitive sensitivity) of both the participant (decision-maker) and the adviser to return the most probable stimulus in the current trial that might have generated the evidence. This represented the participant’s optimal final decision in the current trial, from which we calculated optimal influence. Yellow squares represented probability distributions of the stimulus-related variables (task difficulty and motion direction) and were updated after the responses’ evidence but before the feedback to obtain the most probable stimulus given the evidence. **b** Trial-by-trial parameter estimation of the Bayesian model for relevant advisers’ attributes. The three graphs show how, across trials and with trial-by-trial feedback, the model learned the relevant global attributes (accuracy, mean confidence, and metacognitive sensitivity) of different advisers. Colored dots represented the mean estimated parameter level (across participants) for each trial and each agent. In the model, each parameter was expressed on a scale from 1 (very low) to 10 (very high).

Bayesian modeling aimed to identify optimal behavior in the ADT. We defined optimal behavior as the final decision that, given the information available to the participant at that stage, increased the probability of providing an accurate (final) response. Our approach was to build a decision model (Fig. 3a) that could optimally weigh all available cues on each trial and the learned attributes of both the decision-maker and the adviser in each ADT trial (Fig. 3b). Once all the available information was properly weighted, the model returned the optimal final decision, i.e., the optimal integration of advice.

#### Bayesian network

We built a Bayesian network using the software Genie (GeNIe Modeler, BayesFusion)^50^. Bayesian networks (also called causal networks or belief networks) are acyclic directed graphs composed of nodes and arcs. Nodes represented random variables, whereas arcs represented direct probabilistic relationships between them. Our Bayesian network aimed to model the optimal final decisions that participants could make given the available information. Indeed, the network included all the information a participant could use to make her decision during the ADT, both regarding her own behavior and the adviser’s. These pieces of information were expressed by the nodes (i.e., the decision variables). The model included different types of nodes/variables:

1. **Individual property variables**: These nodes represented latent, initially unknown (i.e., uniformly distributed) and mutually independent causes of the decision-maker/adviser’s behavior, which could be progressively estimated and updated via trial-by-trial feedback. These variables included the participant’s mean accuracy, mean confidence, and metacognitive sensitivity, as well as the agent’s mean accuracy, mean confidence, and metacognitive sensitivity.
2. **Evidence variables**: These nodes expressed the evidence available to the participant during each trial of the ADT, including the participant’s perceptual judgment, the participant’s confidence, the agent’s judgment, and the agent’s confidence. The network did not need to estimate these variables, since the participant knew them at each trial before making the final decision.
3. **Objective properties of the stimulus**: These nodes expressed variable features of the perceptual stimulus, namely the level of coherence of the moving dots and the actual angular direction of the coherent dots. These features were objective properties of the environment, but their actual value—before the feedback—was unknown to the participant and to the agent. Their task in each trial was to estimate the most probable objective angular direction of the stimulus, that is, the stimulus that maximized the probabilities of the observed evidence in that trial (2) given what they had learned up to that trial about their own and the other agent’s individual properties (1).
4. **Error variables**: These nodes modeled the distribution of possible perceptual errors (judgment angular errors) of the decision-maker and the adviser in each trial. Their distribution was determined by the decision-maker/adviser’s accuracy (individual property) and task difficulty (an objective property of the stimulus). Combined with the stimulus’s objective direction, they determined the distribution of the decision-maker/adviser’s judgments (evidence). Together with the individual properties “metacognitive sensitivity” and “mean confidence,” they determined the distribution of the decision-maker/adviser confidence response (evidence).

### Modeling approach

#### General description

**Fig. 4:**
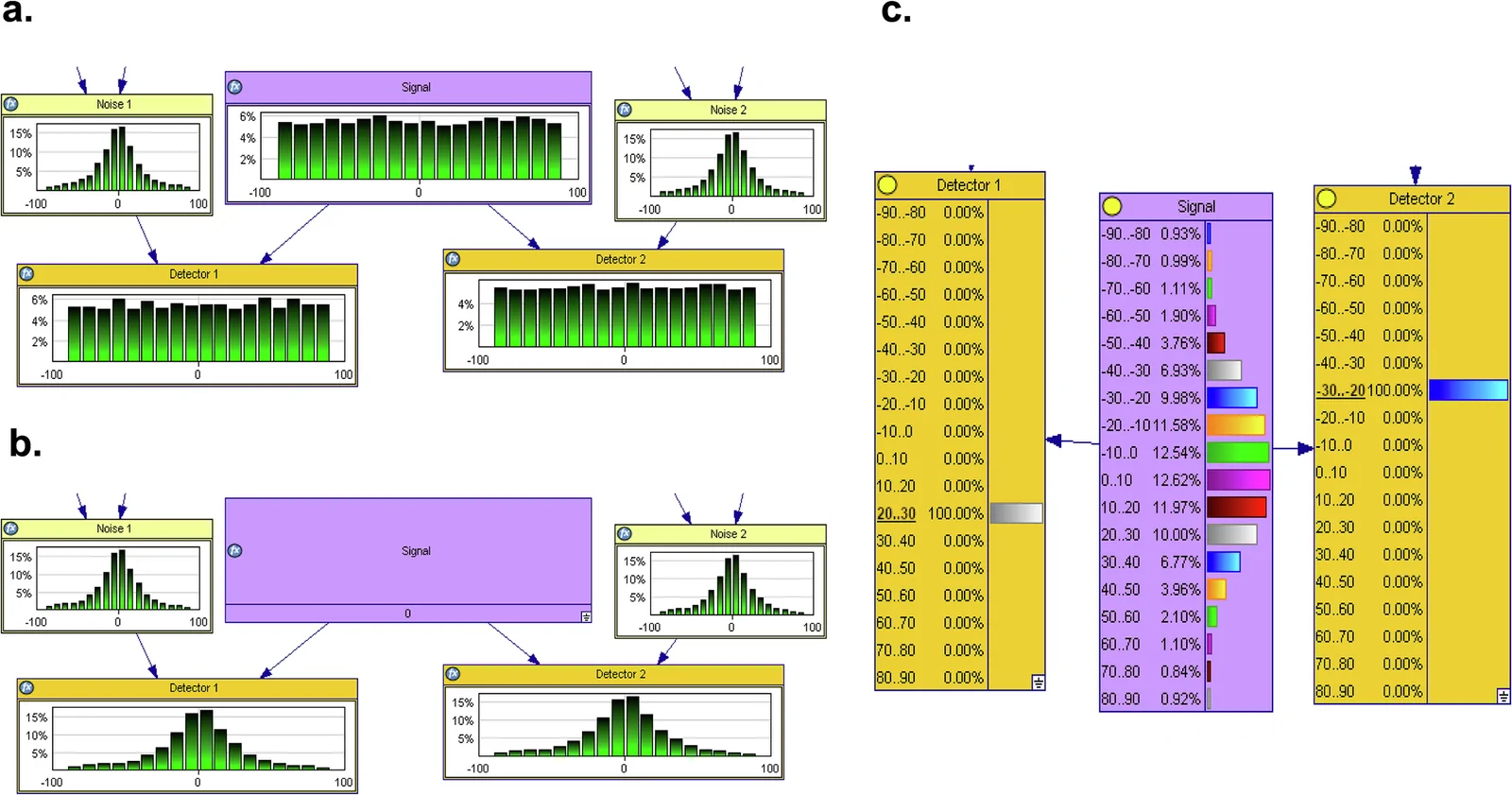
An example of a signal detection model. **a** The priors of the two noise nodes are for illustration purposes only. **b** Unknown to the two detectors, the direction of the objective signal is 0°. Combined with the two noise distributions, it determines the probability distributions of their responses. **c** In the example, detector 1’s response is between 20° and 30°, and detector 2’s response is between −20° and −30°. The evidence updates the probability distribution of the true direction of the actual stimulus.

The model’s core was a probabilistic signal-detection model, with the stimulus direction as the Signal and perceptual error as the noise. An incoming signal, combined with the noise distribution, determined the probability distribution of the two detectors’ judgments. Prior noise distributions were grounded on maximal uncertainty about how skilled the two detectors were at the task (mean accuracy) and about the difficulty of the incoming stimulus (task difficulty). That is, the prior distributions of the n levels of mean accuracy and of the 3 levels of task difficulty (i.e., coherence) were uniform. For each of the n × 3 pairings, the distributions of perceptual errors were estimated by fitting the experimental data into the definitive model. These n × 3 distributions, initially equally weighted (due to uniform priors on accuracies and difficulties), served as the initial prior distributions for the noise nodes. The objective value of the Signal (angular direction), integrated by the noise distributions, determined the direction judgments. Since the Signal distribution was uniform, all judgments were equiprobable before the Signal arrived (example in Fig. 4a). Once the Signal had arrived, the sum of its value and the noise distributions generated the probability distributions of the two detectors’ responses (Fig. 4b). Conversely, if the two detectors’ responses and their noise distributions were known, the inverse probability distribution describing which objective directions of the stimulus maximized the joint probability of observing those two responses could be computed (Fig. 4c).

Of course, once the feedback was provided, the precise errors of both detectors were known. This allowed them to update their noise probability distribution. For example, given that both had gone wrong by 25°, the noise shape that maximized the probability of that error, among the n × 3 available noise shapes, increased its probability. In turn, updating the noise nodes updated the distributions of the probability nodes for both detectors’ accuracy (and of the stimulus difficulty node, but only for the given trial: it stayed uniform in every new trial). The accuracy nodes were updated across all experimental trials, allowing both detectors to learn their own skill levels on the task and to increase precision as trials accumulated. Their learning was exclusively based on observed responses, feedback, and Bayesian updating, starting with noninformative priors, as occurred for ideal rational decision-makers and advisers in the experimental task proper.

The core signal detection model was then extended to include the two confidence responses. For them, the model assumed that confidences were affected by the detector’s estimation of its probable error in each trial, their metacognitive capability to give confidence responses correlated to the probable size of the error (metacognitive sensitivity node), and an individual anchoring with a preference for low or high absolute values of confidence. Following the same logic illustrated for accuracies, as trials accumulated, the metacognitive sensitivity and confidence mean nodes were updated from their initial noninformative distributions to an increasingly accurate estimate of the detectors’ mean confidence and metacognitive sensitivity.

#### Model training

Probabilistic dependencies between variables were defined by fitting probability distributions to simulated data from thousands (i.e., 7680) of artificial agents, combining all possible levels of the Individual properties variables. Training the model on data that encompassed all combinations of these properties (such as mean accuracy, mean confidence, and metacognitive sensitivity) enabled the network to optimally establish probabilistic dependencies between the agents’ individual properties and the evidence variables. This approach ensured the model could generalize across a wider range of agent behaviors. We highlighted that the algorithms used to produce artificial agents were the same as those used to create agents in the ADT (see section “Generating agents’ behavior”): these algorithms were (bio)inspired by data of actual human behavior. Specifically, we created an agent starting from a specific set of parameters (mean accuracy, mean confidence, and metacognitive sensitivity), added random noise to each parameter (±0–0.10), and repeated the process 120 times for each set of parameters, creating 120 agents with similar (but not identical) attributes. Then we moved to a new set of parameters to create another cluster of 30 agents with similar characteristics, and so on. The reference values for the parameters of our three variables of interest were set as follows (Mean accuracy: 0.10–0.90; Mean confidence: 10–90; Metacognitive sensitivity: −0.05–0.75). We used 64 combinations of starting parameters (each of the three variables had 4 possible starting levels, with random noise added).

It was important to note that this data did *not* include participants’ final decisions, since our goal was to estimate the *optimal* final decisions based on the evidence available to participants before they made their final decision. Individual property nodes were discretized into quantiles and used to define distributions for participants and agents based on their mean accuracy, mean confidence, and metacognitive sensitivity, respectively. The between-participant variability across these variables and the contingent values assumed by the stimulus objective properties nodes determined the probability distribution of the hierarchically dependent nodes (Error variables and Evidence variables) based on the causal relationships depicted in Fig. 3a. These probability distributions were derived from simulated artificial data, either the actual data of participants and agents in the experiment or simulated data. Prototypes of the model were fitted to data from pilot participants, and they successfully managed, after simulating all participants’ responses across all experimental trials, to correctly classify participants and agents into their actual accuracy, mean confidence, and confidence reliability quantiles.

#### Estimating optimal final decisions

Once the model was trained, we implemented the following steps for the estimation of the optimal responses:

1. At each trial, the model was fed with the evidence the participant observed (evidence variables).
2. The network estimated the posterior probability distributions of all the hierarchically superior nodes (individual property variables, perceptual error variables, and objective properties of the stimulus variables). The individual property variables were updated between trials, whereas the perceptual error and stimulus property variables were updated within trials and reverted to noninformative priors for each new trial.
3. By looking at the estimated probability distribution of the “Stimulus” node (which was unknown to the network at this stage), we obtained a direct estimate of the most probable angular direction of the current visual stimulus that might have generated the evidence, given all the information available to the participant. The most probable angular direction, extracted from the central tendency of such probability distribution, was interpreted as the “optimal final decision” a rational decision-maker could provide, given the available information.
4. The model was then provided with evidence of the actual angular direction of the visual stimulus in the current trial (i.e., the feedback).
5. Once the model received feedback on the current trial’s correct response, it updated all the probability distributions of the hierarchically superior nodes (including the Individual Properties variables) to refine its estimates of the probability distribution of the relevant variables referred to the participant and the current interacting agent, which were used as prior probabilities for the following trial. The estimates of the Individual properties variables related to the participant continued to update throughout the entire experiment, whereas those referring to the agent were reset to the initial values (i.e., uniform priors within the individual properties nodes) at the beginning of each block, i.e., when the participant started to interact with a new agent. In Experiment 2 (see below), our Bayesian model did not update the Individual properties variables of the agent on a trial basis, but kept them constant within the block and across trials, since the attributes disclosed at the start of the block precisely described the aggregate behavior of the agent along the entire block (see section “Experiment 2”).

#### Quantifying optimal use of advice

Once we had obtained the optimal final decision for each participant and trial, we computed the optimal influence variable. Optimal influence was calculated in the same way as the influence variable (see the paragraph “Derived variables”), but the optimal final decision suggested by the model was used instead of the actual participant’s final decision. Once we obtained a trial-by-trial optimal influence variable, we computed an optimality index as the absolute difference between the optimal and observed influence. Moreover, we computed an individual optimality index by averaging the optimality index across trials.

### Experiment 2

**Fig. 5:**
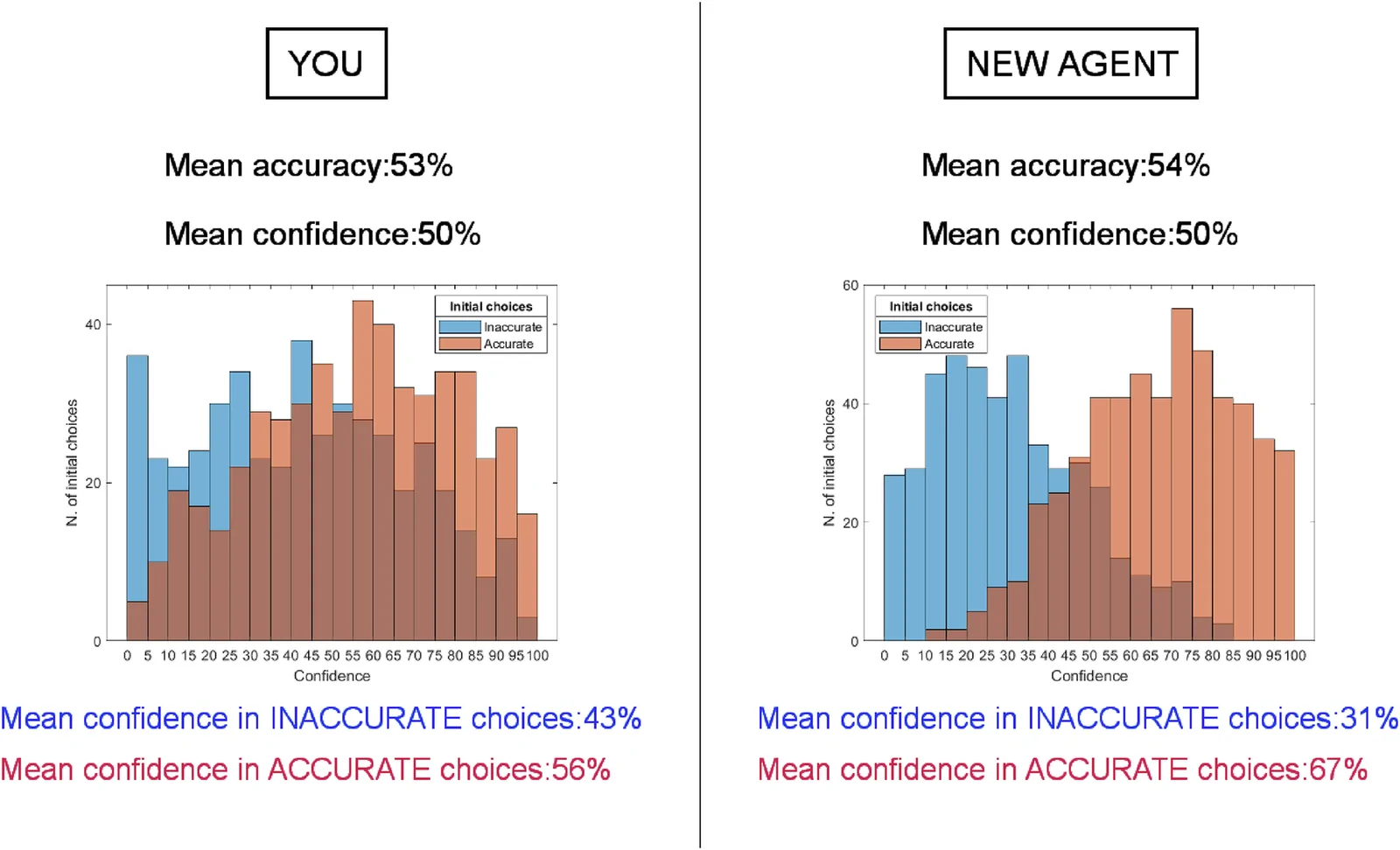
Example screen displaying the behavior of both participants and agents in Experiment 2. This screen appeared at the start of each ADT experimental block. It was divided vertically, with the left side presenting information about the participant and the right side providing details about the current agent. In this example, the participant and the agent had similar mean accuracy and mean confidence, but they differed substantially in their metacognitive sensitivity (participant: low metacognitive sensitivity; agent: high metacognitive sensitivity).

Experiment 2 was identical to Experiment 1 except for one key modification. Before each ADT block (i.e., before interacting with a specific artificial agent), participants were shown detailed information about their own and the agent’s behavioral parameters, including mean accuracy, mean confidence, and metacognitive sensitivity, on a dedicated screen (Fig. 5). Both the participant’s and the agent’s mean accuracy and mean confidence were provided as numerical percentages. Participant data were drawn from previous ADT blocks (or from the IDT for the first block), using the same data later used to generate the next agent. Agent data were based on the exact set of 54 trials in the upcoming block.

To facilitate the interpretation of metacognitive sensitivity, defined as a point-biserial correlation coefficient, we combined graphical and numerical representations. A graph illustrated the distribution of confidence judgments for accurate versus inaccurate perceptual decisions, with greater overlap indicating lower metacognitive sensitivity and less overlap indicating higher metacognitive sensitivity. During the instructions, the presented graph was carefully described to participants in terms of its core components (x- and y-axes, bars, and colors). Participants were therefore explicitly instructed on how to read the graph, with illustrative examples. However, they were not instructed on how to use this information during the advice-taking process. Additionally, the mean confidence values for accurate and inaccurate judgments were shown; larger differences reflected higher metacognitive sensitivity. Participant metacognitive sensitivity was drawn from previous ADT blocks (or the IDT for the first block), while agent metacognitive sensitivity was based on 1000 simulated trials using the agent’s behavioral parameters. Participants were informed that their parameters reflected past performance, whereas the agent’s parameters represented the agent’s typical behavior in the upcoming block. Consequently, in Experiment 2, the Bayesian model kept the agent’s individual properties constant within each block, while participant properties continued to update across trials and blocks to capture potential fluctuations over time.

## Results

In the Advised Decision Task (ADT; Fig. 2a), participants interacted with a series of overtly artificial advisers. On each trial, they first estimated the global motion direction in a Random Dot Kinematogram and rated their confidence as the probability of being correct. They then received the adviser’s judgment and confidence rating for the same stimulus. Based on this advice, participants revised their estimate. Finally, they received feedback on the true direction. Adviser behavior was systematically manipulated along three key dimensions: (1) accuracy, (2) mean confidence, and (3) metacognitive sensitivity (Fig. 2b). To quantify advice use, we defined influence as the relative adjustment of the participant’s final decision toward the adviser’s estimate (Fig. 2c).

We defined optimal behavior in the ADT as the final decision that maximized the probability of an accurate response, given all information available to the participant at that trial. To formalize this, we implemented a Bayesian decision model (Fig. 3a) that integrates all available cues on each trial and the learned attributes of both the decision-maker and the adviser (Fig. 3b).

## Experiment 1: adaptation through trial-by-trial feedback

In Experiment 1, participants adapted their advice use solely through trial-by-trial feedback. They received no explicit information about advisers’ characteristics, which had to be inferred gradually from performance outcomes. This design mirrors real-life situations in which people judge their own and others’ competence by observing repeated successes and failures rather than relying on an external authority.

### Advice enhances accuracy, but below the optimal benchmark

First, we test whether advice enhanced participants’ accuracy, and whether such improvement was comparable to or lower than that predicted by our Bayesian model of optimal advice integration.

**Fig. 6:**
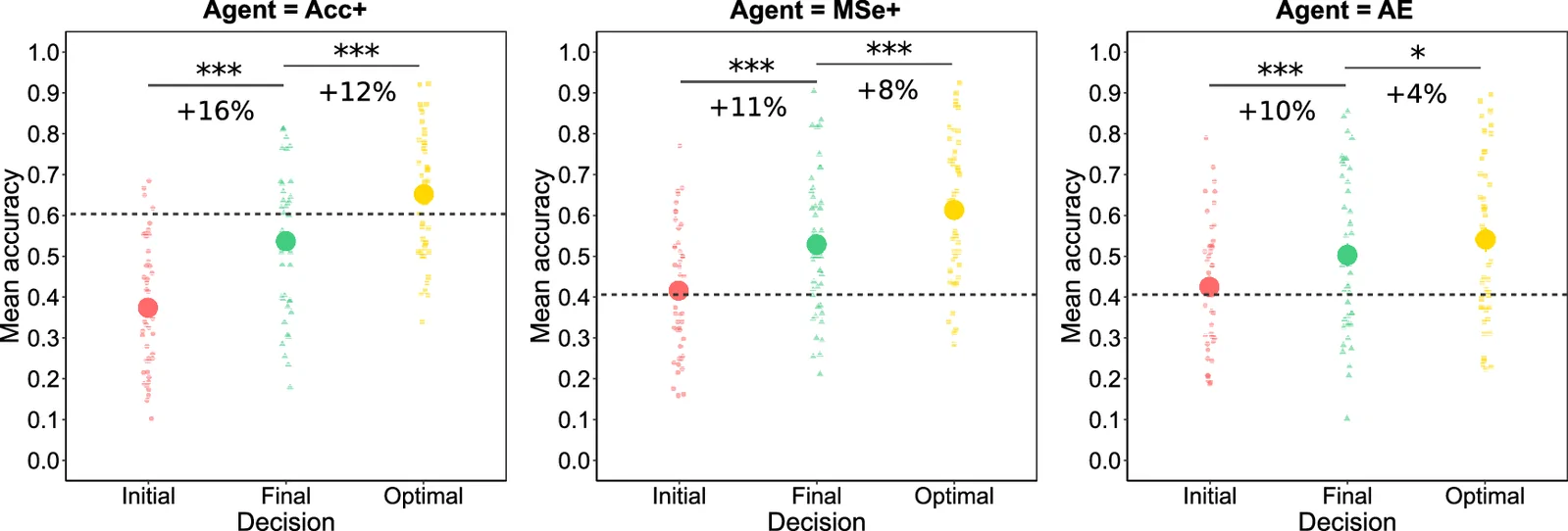
Observed and optimal decision accuracy. Mean accuracy of participants’ initial and final decisions and accuracy of the optimal decision predicted by Bayesian modeling for interactions with agents Acc+, MSe+, and AE (*N* = 46). Little shapes represent individual mean accuracies, while big dots represent the sample mean. Dotted lines represent the adviser’s mean accuracy across participants. Asterisks represent results of Model 1.1 and Model 1.2: *p < 0.05, ***p < 0.001. Full results in the SI (Supplementary Tables S3–S5).

Results of Model 1.1, which compares participants’ initial and final accuracy across adviser types, showed that participants’ decision accuracy improved when interacting with most advisers, except when the adviser was less accurate than the participant (Tables S1–S4, SI). On average, accuracy increased by 9%, from 42% to 51%, across the seven adviser conditions. The greatest improvement (Fig. 6) was observed in blocks with an adviser who was more accurate than the participant (Acc+: +16%) and in blocks with an adviser who exhibited high metacognitive sensitivity (MSe+: +11%). We did not observe a worsening of performance in any condition.

Final accuracy exceeded both the participant’s and the adviser’s whenever their accuracies were similar. With a less accurate adviser (Acc–), final accuracy stayed close to the initial level, but, importantly, it did not decline (equivalence test, H_0_ = Initial - Final ≥ 0.05, TOST(45) = −3.1, *p* = 0.002). With a more accurate adviser (Acc+), accuracy improved substantially (+16%), but it still did not reach the level of the adviser (−6%).

Results are equivalent when the first third of trials within each block was excluded to control for an initial learning phase (see Model 1.1b. See also the supplementary section “Temporal and learning effects,” which illustrates learning effects within experimental blocks).

Then we compared the participant’s final accuracy with that of the Bayesian model. Results of Model 1.2 (Fig. 6, Supplementary Table S5) showed significant gaps between the model’s optimal accuracy and participants’ final accuracy in several adviser conditions: Acc+ (+12%), MSe+ (+8%), AE (+4%), and Conf− (+4%). In the other three conditions, the participants’ final accuracy and optimal one were statistically comparable: Conf+ (+3%), Acc- (+1%), MSe- (+0%). Although participants substantially improved their accuracy when interacting with advisers who are either more accurate (Acc+) or exhibit high metacognitive sensitivity (MSe+), these were also the contexts in which their advice integration deviated most from optimality. Participants underutilized the potential benefit of high-quality advice, resulting in a measurable loss in achievable accuracy.

### Advice is used flexibly upon decision-makers’ and advisers’ attributes

To investigate the sources of suboptimality in participants’ advice integration, we first tested whether they modulate the weight assigned to advice based on the relative difference between their own and the current adviser’s behavioral attributes (Model 1.3). Rational expectations are summarized in Table 1. For the analysis, we excluded the first third of each block, assuming that participants required an initial learning period to infer the adviser’s characteristics and calibrate their behavior (see “Temporal and learning effects” in the SI). Indeed, the results indicate that participants’ behavior tends to stabilize toward the end of the first third of the experimental block, suggesting that the knowledge acquired during this period was sufficient to guide their subsequent advice-related behavior.

**Table 1:** Summary of expected rational use of advice based on decision-makers’ and advisers’ attributes

Attribute	Rational expectation
**Accuracy**	Influence should increase (or decrease) as the accuracy gap between the adviser and the decision-maker widens. Influence should be ≈0.5 when their accuracies are equal.
**Average confidence**	When the adviser and decision-maker have similar accuracies, differences in mean confidence should not affect the influence. Confidence should be rescaled to allow unbiased comparisons based on trial-by-trial confidence judgments.
**Metacognitive sensitivity**	The higher the adviser’s metacognitive sensitivity, the more the decision-maker should weigh confidence on a trial-by-trial basis, and vice versa.

First, participants exhibited a mean influence of 0.36 (SD = 0.10; model intercept (vs. 0): B = 0.37, SE = 0.01, t = 26.35, *p* < 0.001, 95% CI [0.35, 0.40]), which was significantly lower than the reference value of 0.5 (one-sample Wilcoxon signed-rank test: W = 40, *p* < 0.001, 95% CI [−0.17, −0.11], r_rb_ = −0.93). This reference value is based on the finding that advisers were, on average, as accurate as participants (participants’ accuracy: M = 0.46, SD = 0.15; advisers’ accuracy: M = 0.45, SD = 0.13). Given this symmetry in accuracy, an optimal agent should weight both opinions equally (i.e., influence = 0.5).

Participants adjusted their reliance on advice based on both their own attributes and the adviser’s attributes (Supplementary Table S6). Appropriately, they placed greater weight on advice as the difference in accuracy between the adviser and themselves increased (Δ *mean accuracy* [agent–subject]: B = 0.29, SE = 0.02, t = 14.30, *p* < 0.001, 95% CI [0.25, 0.33]). However, a similar pattern also emerged with differences in mean confidence (Δ *mean confidence* [agent–subject]: B = 0.28, SE = 0.03, t = 10.59, *p* < 0.001, 95% CI [0.22, 0.33]), such that participants were more influenced when the adviser’s average confidence was higher, regardless of the adviser’s actual accuracy.

In addition to global features such as accuracy and mean confidence, participants also considered trial-specific differences in confidence (Δ *confidence)* between themselves and the adviser. Specifically, they were more likely to follow advice when the adviser reported higher confidence than they did on a given trial, after normalizing for individual mean confidence (Δ *confidence* [agent–subject]: B = 0.51, SE = 0.01, t = 49.73, *p* < 0.001, 95% CI [0.49, 0.53]). Crucially, the effect of this trial-by-trial confidence difference was moderated by another global feature shared by both participants and advisers: metacognitive sensitivity. Participants were more responsive to trial-by-trial confidence signals when the adviser’s confidence was more predictive than their own (interaction: Δ *metacognitive sensitivity* × Δ *confidence*: B = 0.10, SE = 0.04, t = 2.59, *p* = 0.010, 95% CI [0.03, 0.18]), reflecting a rational calibration of influence based on comparative metacognitive performance.

### Advice use is adaptive, but suboptimal

After establishing that participants adjusted their advice-taking behavior as a function of both their own attributes and those of the adviser (Model 1.3), we next fitted an additional mixed-effects model (Model 1.4). This model included the same predictors as Model 1.3 and additionally incorporated interactions with the factor *influence type* (real influence vs. optimal influence), allowing us to directly assess how participants’ attribute-based modulation of advice use compares with the optimal influence predicted by our Bayesian model.

**Fig. 7:**
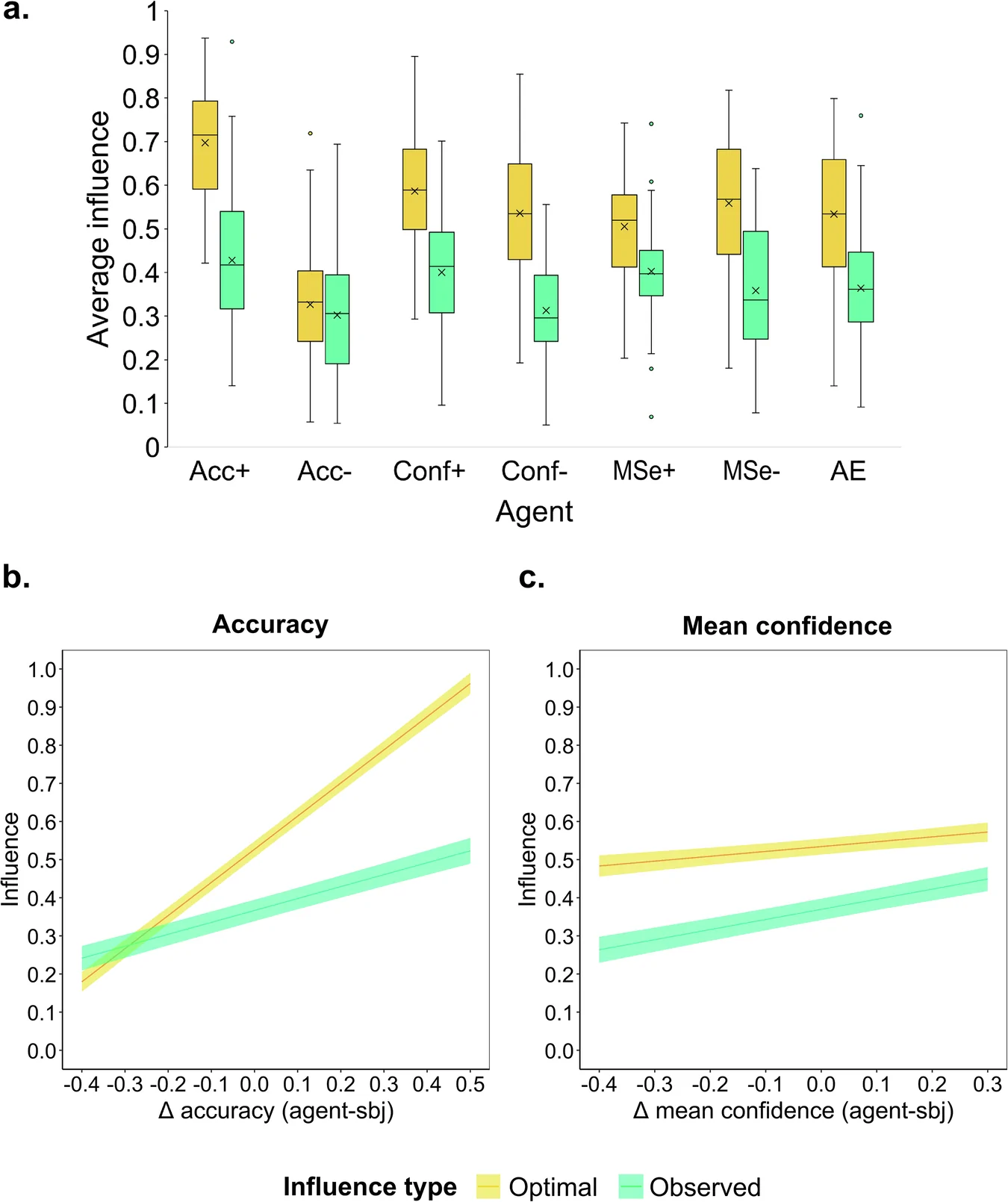
Modulation of advice use. **a** Boxplots of participants’ mean influence (observed in green, optimal in gold) across the seven adviser conditions (*N* = 46). The box encompasses the interquartile range (IQR) with the internal horizontal line representing the median and the ‘X’ marker indicating the arithmetic mean. Whiskers extend to the maximum and minimum values within 1.5*IQR, while individual points denote statistical outliers. The first third of trials in each block was excluded from the analysis. **b** Model 1.4 estimates (unstandardized betas) of participants’ influence as a function of Δ* accuracy* [agent–subject] by influence type (observed in green, optimal in gold). Ribbons express 95% confidence intervals of the regression estimates. Estimates based on data from 46 participants. **c** Model 1.4 estimates (unstandardized betas) of participants’ influence as a function of Δ *mean confidence* [agent–subject] by influence type (observed in green, optimal in gold). Ribbons express 95% confidence intervals of the regression estimates. Estimates based on data from 46 participants.

Results (Supplementary Table S7) indicated that participants’ influence systematically deviated from the normative benchmark across all dimensions. Bayesian modeling revealed robust evidence of egocentric advice discounting, as shown by a significant main effect of *influence type* (real vs. optimal): B = −0.16, SE = 0.01, t = −11.92, *p* < 0.001, 95% CI [−0.19, −0.14]. Participants’ influence was largely below the optimal level across all adviser types, remaining under 0.5 even with Acc+ (Fig. 7a). In other words, they trusted their own judgment more, even when the adviser was more competent.

Participants also under-modulated their influence in response to differences in mean accuracy, as indicated by a significant interaction between Δ *mean accuracy* and *influence type* (B = −0.56, SE = 0.03, t = −20.31, *p* < 0.001, 95% CI [−0.62, −0.51]). This suggests that while participants used accuracy as a cue to guide their reliance on advice, they did so to a much lesser extent than would be expected under optimal Bayesian integration (Fig. 7b). In contrast, participants were overly sensitive to a non-normative cue such as Δ *mean confidence* (B = 0.15, SE = 0.03, t = 4.26, *p* < 0.001, 95% CI [0.08, 0.22], Fig. 7c). This indicates that participants placed disproportionate weight on advisers who expressed higher average confidence, even when these advisers were no more accurate.

**Fig. 8:**
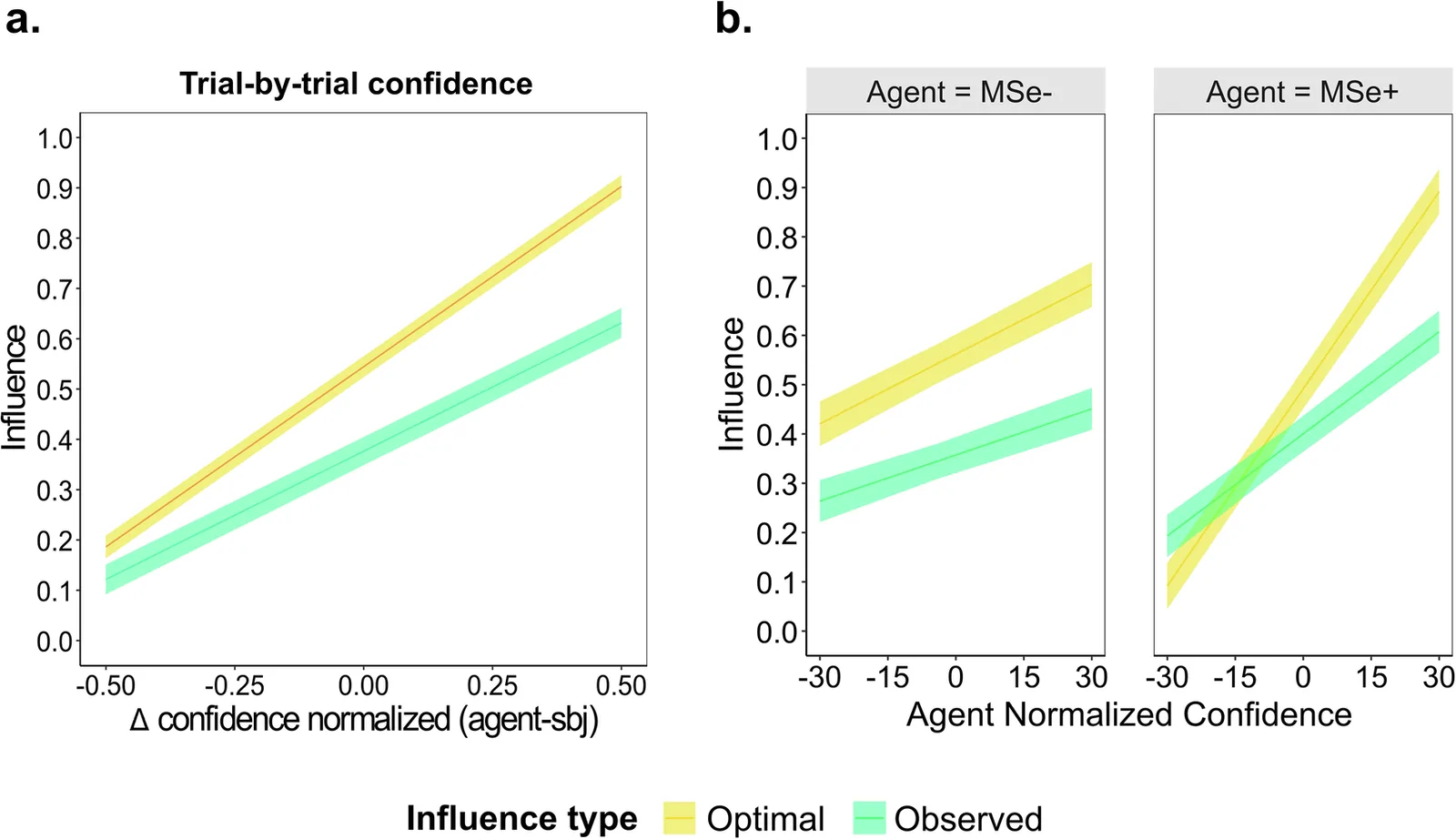
Use of trial-by-trial confidence. **a** Model 1.4 estimates (unstandardized betas) of participants’ influence as a function of Δ *confidence* *normalized* [agent - subject] by influence type (observed in green, optimal in gold). Ribbons express 95% confidence intervals of the regression estimates. Estimates based on data from 46 participants. **b** Model 1.4 estimates (unstandardized betas) of participants’ influence as a function of the *agent’s normalized* (trial-by-trial) *confidence* by *influence type* (observed in green, optimal in gold). The left panel refers to interactions with the agent MSe-, the right panel to interactions with the agent MSe+. Ribbons express 95% confidence intervals of the regression estimates. Estimates based on data from 46 participants.

Participants’ influence was also insufficiently modulated by local cues. In particular, the effect of Δ (trial-by-trial) *confidence* on advice-taking was significantly reduced relative to the optimal model (B = −0.21, SE = 0.01, t = −15.35, *p* < 0.001, 95% CI [−0.24, −0.18]), indicating that participants underutilized differences in confidence between themselves and the adviser (Fig. 8a). Furthermore, participants failed to appropriately adjust their use of Δ *confidence* based on both their own and the adviser’s metacognitive sensitivity. This was evidenced by a significant three-way interaction between Δ *metacognitive sensitivity*, Δ *confidence*, and *influence type* [real-optimal]: B = −0.20, SE = 0.05, t = −3.60, *p* < 0.001, 95% CI [−0.31, −0.09]. In other words, participants did not sufficiently increase their reliance on advice when the adviser was confident, and the adviser’s confidence was highly predictive of correctness (Fig. 8b).

### Emerging biases in the processing of confidence signals

Results from Model 1.4 revealed that participants tended to overrely on overconfident advisers while simultaneously underweighting highly predictive trial-level confidence signals. To better understand the mechanisms underlying these biases, we ran two variants of Model 1.4.

**Fig. 9:**
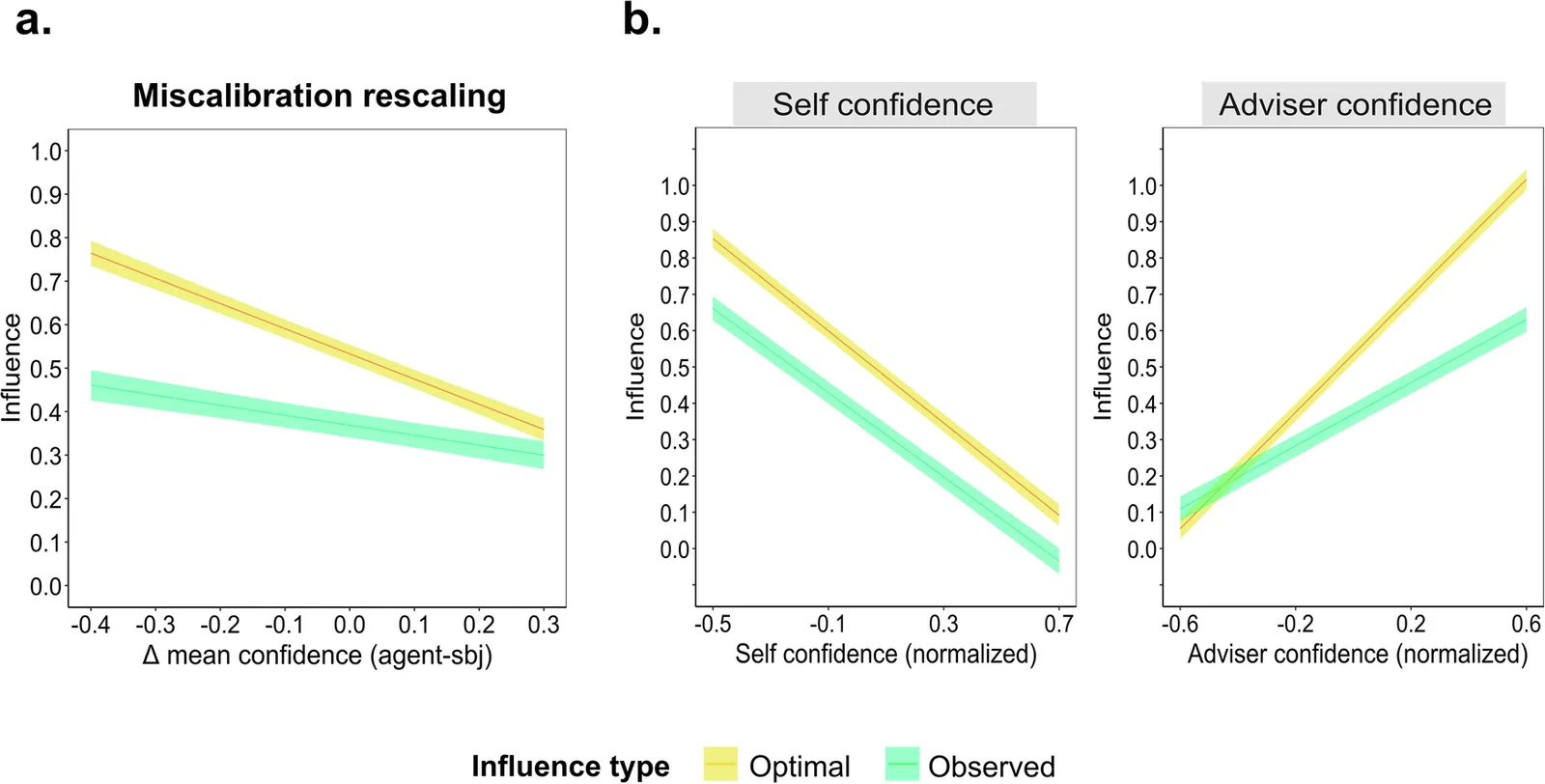
Use of trial-by-trial confidence. **a** Model 1.4b estimates (unstandardized betas) of participants’ influence as a function of Δ *mean confidence* [agent - subject] by influence type (observed in green, optimal in gold). Ribbons express 95% confidence intervals of the regression estimates. Estimates based on data from 46 participants. **b** Model 1.4c estimates (unstandardized betas) of participants’ influence as a function of the agent’s normalized trial-by-trial confidence by influence type (observed in green, optimal in gold). The left panel refers to the *self confidence* regressor, the right panel to the *adviser confidence* regressor. Ribbons express 95% confidence intervals of the regression estimates. Estimates based on data from 46 participants.

First, we hypothesized that the overreaction to non-predictive mean confidence reflected a failure to rescale miscalibrated confidence. To test this hypothesis, we ran a variant of Model 1.4 (Model 1.4b, Supplementary Table S8) in which the Δ *confidence* [agent–subject] was not normalized by the adviser’s and participant’s mean confidence. In this formulation, the coefficient for Δ *mean confidence* captures the extent to which participants rescaled confidence signals based on overall calibration. After controlling for accuracy, appropriate rescaling should reduce the influence of the adviser’s mean confidence as it increases. The results (Fig. 9a) show that participants did rescale confidence (B = −0.23, SE = 0.03, t = −8.65, *p* < 0.001, 95% CI [−0.28, −0.18]), but the adjustment was markedly weaker than optimal (interaction with *influence type* [real – optimal]: B = 0.35, SE = 0.04, t = 9.45, *p* < 0.001, 95% CI [0.28, 0.42]).

Second, we test for asymmetries in the weight assigned to self- versus other-confidence on a trial-by-trial basis (Model 1.4c, Supplementary Table S9). Specifically, we hypothesized that the insufficient modulation of influence by Δ *confidence* was primarily driven by a selective underweighting of the adviser’s, rather than the participant’s, confidence signal. Results showed that, when compared to an optimal benchmark, both confidence signals were underused (real – optimal: *self confidence* B = 0.05, SE = 0.02, t = 2.78, *p* = 0.005, 95% CI [0.02, 0.09]; *adviser confidence* B = −0.37, SE = 0.02, t = −18.49, *p* < 0.001, 95% CI [−0.41, −0.33]; Fig. 9b). Importantly, the underweighting was far more pronounced for a*dviser confidence* (–0.37) than for *self-confidence* (0.05), a difference of over sevenfold. These results highlight that participants not only discounted the adviser’s opinion but also underutilized the adviser’s confidence signal, leading to a much weaker-than-optimal adjustment of influence based on the adviser’s confidence. In particular, participants did not accord sufficient weight to the adviser’s opinion when the adviser was highly confident (Fig. 9b). Taken together, these findings suggest that the reduced sensitivity to Δ (trial-by-trial) *confidence* primarily reflected a neglect of the adviser’s confidence, rather than a general failure to integrate confidence information.

### Does suboptimality depend on erroneous attribute estimates?

The observed suboptimality may originate from difficulty estimating advisers’ attributes (e.g., I do not realize that the adviser is more accurate than me), rather than from flawed integration per se.

At the end of each experimental block, we collected participants’ explicit estimates of their own and their advisers’ relevant attributes. On average, participants tended to overestimate both their own accuracy and that of the adviser (estimated self-accuracy: 55%; actual self-accuracy: 46%; estimated adviser accuracy: 56%; actual adviser accuracy: 45%). Crucially, the mean estimated accuracy was comparable between the participant and the adviser. We observed a strong relationship between actual accuracy and estimated mean accuracy (Model 1.5, Supplementary Table S10) for both self-assessment (B = 0.65, SE = 0.06, t = 11.17, *p* < 0.001, 95% CI [0.53, 0.76]) and agent’s assessment (B = 0.49, SE = 0.05, t = 9.67, *p* < 0.001, 95% CI [0.39, 0.60]). Breaking down participants’ ratings for self vs. adviser performance across agents (Model 1.6, Supplementary Table S11), participants noticed that Acc+ and Acc- differ in accuracy (B = −15.83 SE = 2.56, t = −6.18, *p* < 0.001, 95% CI [−20.86, −10.79]). Moreover, they correctly identified the agent Acc+ as more accurate than them (Adviser - Self: B = 9.96, SE = 2.56, t = 3.89, *p* < 0.001, 95% CI [4.92, 14.99]) and the agent Acc- as less accurate than them (Adviser - Self: B = −5.96, SE = 2.56, t = −2.33, *p* = 0.020, 95% CI [−10.99, −0.92]). The high metacognitive sensitivity of agent MSe+ enhanced the perception of its accuracy (Adviser - Self: B = 6.70, SE = 2.56, t = 2.61, *p* = 0.009, 95% CI [1.66, 11.73]). For all the other agents, the self and agents’ estimates were not statistically different (see Supplementary Table S11). Similar results were observed for estimated mean confidence (Models 1.7–1.8, Supplementary Tables S12, S13). For metacognitive sensitivity (Models 1.9–1.10, Supplementary Tables S14, S15), we found a significant positive relationship between actual and estimated agent metacognitive sensitivity (B = 0.34, SE = 0.11, t = 3.23, *p* = 0.001, 95% CI [0.13, 0.54]). Despite this, participants’ estimates of MSe+ and MSe- metacognitive sensitivity did not differ significantly (B = −4.22, SE = 3.43, t = −1.23, *p* = 0.219, 95% CI [−10.95, 2.51]).

Overall, participants provided reasonably good estimates of their own and advisers’ performance and mean confidence, but failed to detect differences in advisers’ metacognitive sensitivity. This indicates that suboptimal advice use cannot be explained solely by misestimation; metacognitive sensitivity was particularly difficult to infer from feedback, and this may have contributed to integration failures.

## Experiment 2: adaptation through explicit disclosure of attributes

To further test the role of attribute misestimation, we designed a second version of the task in which participants were explicitly informed of both their own and their adviser’s attributes before each block, *in addition* to receiving trial-by-trial feedback (Fig. 5). By removing uncertainty about attribute estimation, Experiment 2 isolated the integration process itself, allowing us to assess whether suboptimal advice use persisted even when all relevant information was transparently available. Results of the inter-block estimates of advisers’ attributes indicate that, in Experiment 2, participants were able to discriminate agents’ skills across all relevant dimensions (See SI, “Inter-block performance ratings—Experiment 2” section). This confirms that the additional information provided in Experiment 2 effectively supported participants in identifying advisers’ attributes beyond what could be learned from trial-by-trial feedback alone.

We ran the same analyses as in Experiment 1, and the results largely mirrored those findings despite the additional information provided at the start of each block. Participants improved their accuracy after advice from all advisers, except the least accurate one (Model 2.1). On average, accuracy increased by 10% (from 43% to 53%), with the largest gains observed with advisers who were more accurate (+17%) or showed high metacognitive sensitivity (+15%; Supplementary Tables S16, S18, S19). Final accuracy exceeded both individual judgments whenever participant and adviser had similar accuracy, matched the initial level with Acc− (equivalence test, H_0_ = Initial - Final ≥ 0.05, TOST(42) = −1.91, *p* = 0.031), and remained below the adviser’s accuracy in Acc+ (–4%).

**Fig. 10:**
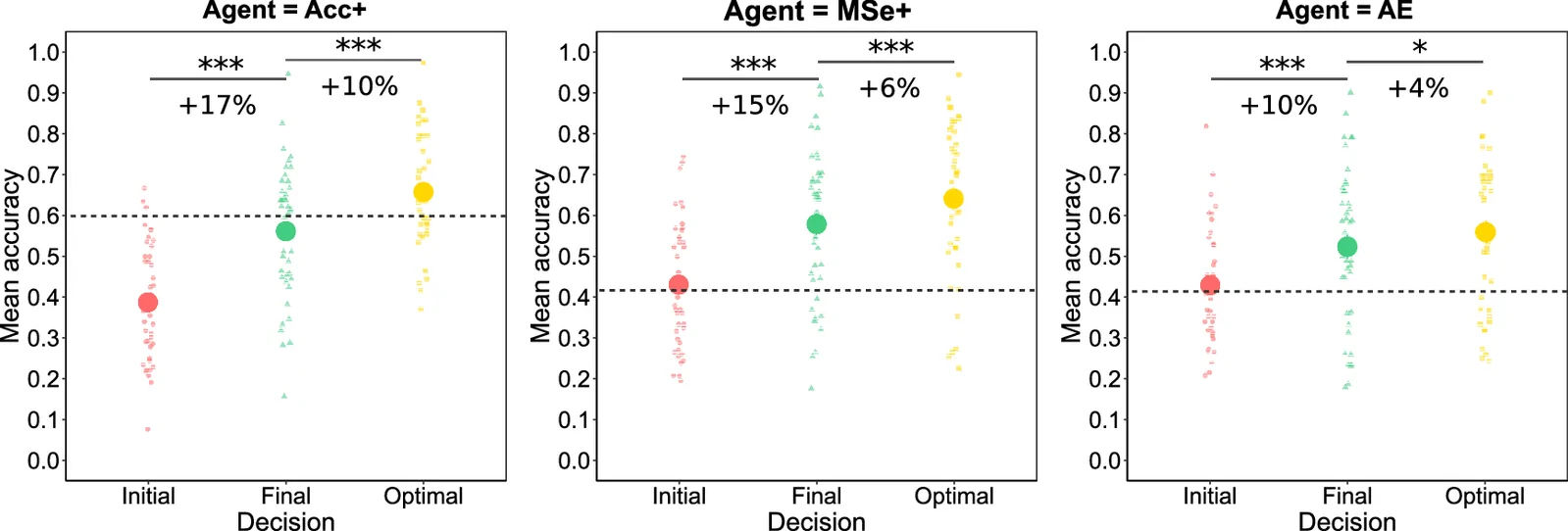
Observed and optimal decision accuracy (Exp 2). Mean accuracy of participants’ initial and final decisions and accuracy of the optimal decision predicted by Bayesian modeling for interactions with agents Acc+, MSe+, and AE (*N* = 43). Little shapes represent individual mean accuracies, while big dots represent the sample mean. Dotted lines represent the adviser’s mean accuracy across participants. Asterisks represent results of Model 2.1 and Model 2.2: *p < 0.05, ***p < 0.001.

The observed accuracy enhancement was inferior to that predicted by optimal Bayesian weighting (Model 2.2, Fig. 10) in interaction with agents Acc+ (enhancement: +10%), MSe+ (+6%), and AE (+4%), but matched the Bayesian benchmark in the other four conditions (Supplementary Tables S16, S20).

Analyzing participants’ advice use (Model 2.3), we found persistent evidence of egocentric advice discounting: notwithstanding an average accuracy across all agents identical to the one of the participant the mean influence was below the rational 0.5 level (mean influence = 0.35, SD = 0.09, one-sample Wilcoxon signed-rank test (vs. 0.5): W = 11, *p* < 0.001, 95% CI [−0.17, −0.12], r_rb_ = −0.98). As in Experiment 1, participants modulated their reliance on advice according to both their own and the adviser’s attributes: mean accuracy, mean confidence, trial-by-trial confidence, and the interaction between trial-level confidence and metacognitive sensitivity (Supplementary Table S21).

We then examined deviations from observed versus optimal behavior (Model 2.4), as defined through Bayesian modeling. The Bayesian benchmark was identical to that of Experiment 1 but incorporated the additional information about advisers’ attributes now available to both participants and the model (see “Methods,” Experiment 2).

Results (Supplementary Figs. S2–S4, Supplementary Tables S22–S24) revealed that, despite this enriched information, participants’ influence weights diverged from optimal integration in ways that closely mirrored those in Experiment 1. Specifically, they systematically underweighted advice overall, relied insufficiently on mean accuracy, and overrelied on mean confidence, failing to appropriately downscale its role when it was miscalibrated (Model 2.4b). Trial-by-trial confidence was also underutilized and insufficiently modulated by its predictive value, with the shortfall arising primarily from an underweighting of the adviser’s confidence signal relative to participants’ own (Model 2.4c).

Together, these findings replicated Experiment 1 and strengthened the conclusion that although participants were sensitive to relevant informational cues, their adjustments remained quantitatively inadequate.

To test the impact of information disclosure on the optimality of advice integration, we used Model 3.1 (Supplementary Table S25) to directly compare trial-by-trial advice-weighting optimality (optimality index; see “Methods”) across the two experiments (*treatment* factor). Critically, we found no main effect of *treatment* (Experiment 2 vs. Experiment 1: B = 0.01, SE = 0.02, t = 0.72, *p* = 0.471, 95% CI [−0.02, 0.05]), suggesting that disclosing the participant’s and adviser’s attributes did not significantly improve the optimality of advice integration. Furthermore, the degree of (sub)optimality in the use of key informational cues was largely comparable across experiments. Specifically, we observed no significant interactions between *treatment* and Δ *mean confidence*, Δ *metacognitive sensitivity*, or the Δ *metacognitive sensitivity* × Δ *confidence interaction* (see Supplementary Table S25). By contrast, the effect of Δ *confidence* showed a significant treatment interaction (B = 0.05, SE = 0.01, t = 3.34, *p* < 0.001, 95% CI [0.02, 0.07]), indicating that participants in Experiment 2 were closer to optimal weighting when the adviser was more confident than themselves.

Taken together, these results indicated only a limited effect of disclosure attributes on advice integration.

### Metacognitive skills predict optimality in advice integration

In this section, we tested whether individual *metacognitive* abilities, such as metacognitive sensitivity and calibration (i.e., metacognitive bias), support optimal advice integration.

Trial-by-trial confidence was predictive of their perceptual judgment accuracy in both experiments (Experiment 1: B = 0.03, SE = 0.00, z = 28.67, *p* < 0.001, 95% CI [0.03, 0.03]; Experiment 2: B = 0.02, SE = 0.00, z = 26.06, *p* < 0.001, 95% CI [0.02, 0.03]). Confidence was higher for easier perceptual stimuli (i.e., a higher percentage of dots moving coherently) in both experiments (Experiment 1: F(2, 17340) = 139, *p* < 0.001; Experiment 2: F(2, 16209) = 168, *p* < 0.001). Similarly, perceptual difficulty predicted judgment accuracy (Experiment 1: X^2^(2, 17384) = 294, *p* < 0.001; Experiment 2: X^2^(2, 16250) = 294, *p* < 0.001). Higher confidence was associated with faster RTs in participants’ initial perceptual judgments (Experiment 1: B = −3.17, SE = 0.12, t = −27.00, *p* < 0.001, 95% CI [−3.20, −2.94]; Experiment 2: B = −2.93, SE = 0.11, t = −26.60, *p* < 0.001, 95% CI [−3.15, −2.72]) and final decisions (Experiment 1: B = −1.23, SE = 0.08, t = −15.40, *p* < 0.001, 95% CI [−1.39, −1.07]; Experiment 2: B = −1.40, SE = 0.08, t = −16.50, *p* < 0.001, 95% CI [−1.57, −1.23]). At the group level, participants’ mean confidence predicted mean accuracy (B = 0.54, SE = 0.10, t = 5.24, *p* < 0.001, 95% CI [0.34, 0.75]), with no difference across experiments (interaction: B = −0.22, SE = 0.21, t = −1.04, *p* = 0.300, 95% CI [−0.63, 0.20]).

**Fig. 11:**
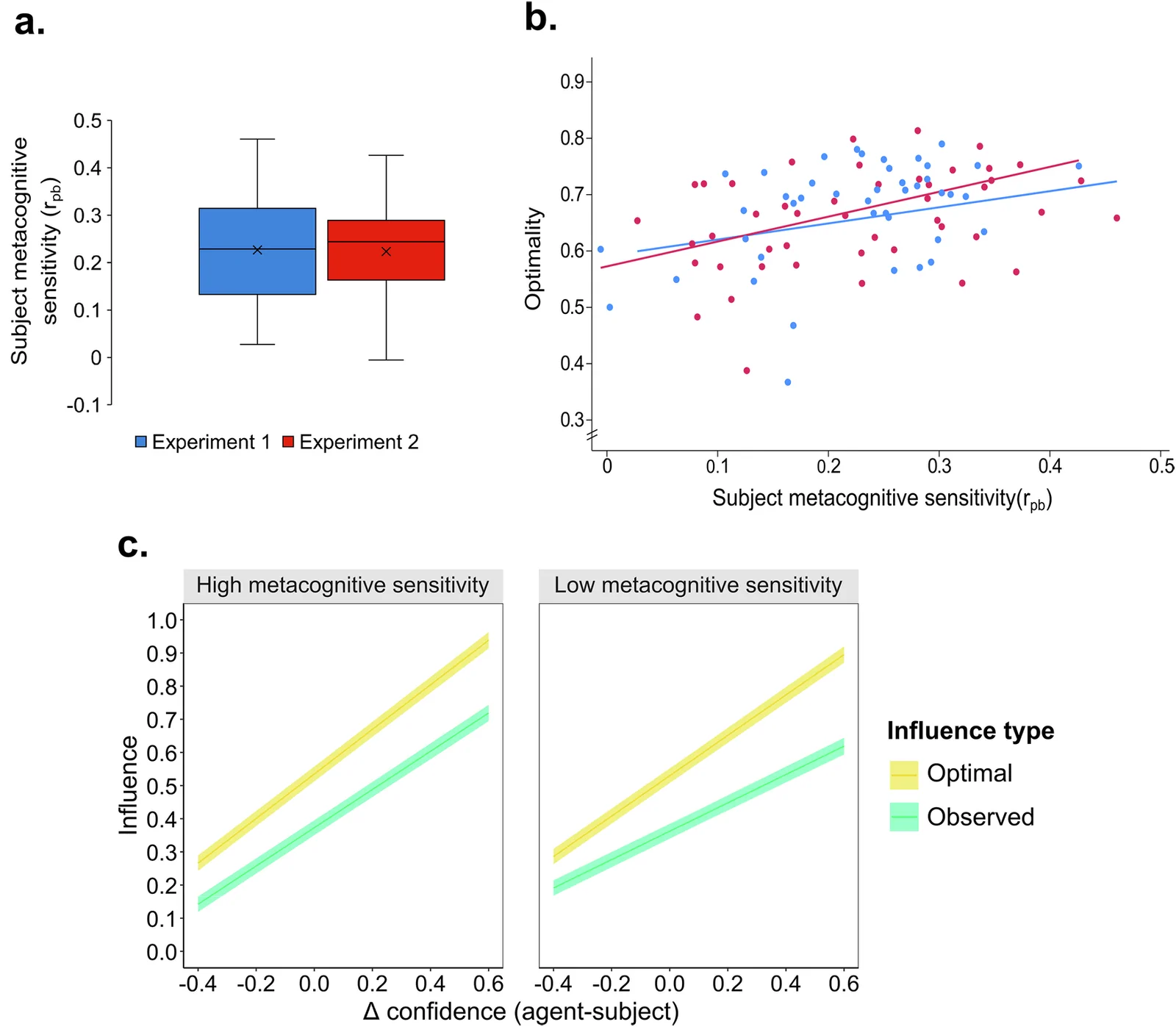
Metacognitive sensitivity and optimality. **a** Boxplots of participants’ metacognitive sensitivity (r_pb_) by experiment (*N* = 89). The box encompasses the IQR with the internal horizontal line representing the median and the ‘X’ marker indicating the arithmetic mean. Whiskers extend to the maximum and minimum values within 1.5*IQR. **b** Scatterplot of individual subject metacognitive sensitivity and optimality, by experiment (*N* = 89). **c** Model 3.2 estimates (unstandardized betas) of participants’ influence as a function of *Δ* (normalized) trial-by-trial *confidence* (agent-subject) by *influence type* (observed in green, optimal in gold). The left panel refers to participants with high metacognitive sensitivity, and the right panel to participants with low metacognitive sensitivity, as defined by a median split at the individual level of metacognitive sensitivity. Ribbons express 95% confidence intervals of the regression estimates. Estimates based on data from 89 participants.

Participants’ individual levels of metacognitive sensitivity were comparable across experiments (Exp1: M = 0.23, SD = 0.11; Exp2: M = 0.22, SD = 0.09; Mann–Whitney U test: U = 988, *p* = 0.997, 95% CI [−0.05, 0.05], r_rb_ = 0.00; Fig. 11a).

**Fig. 12:**
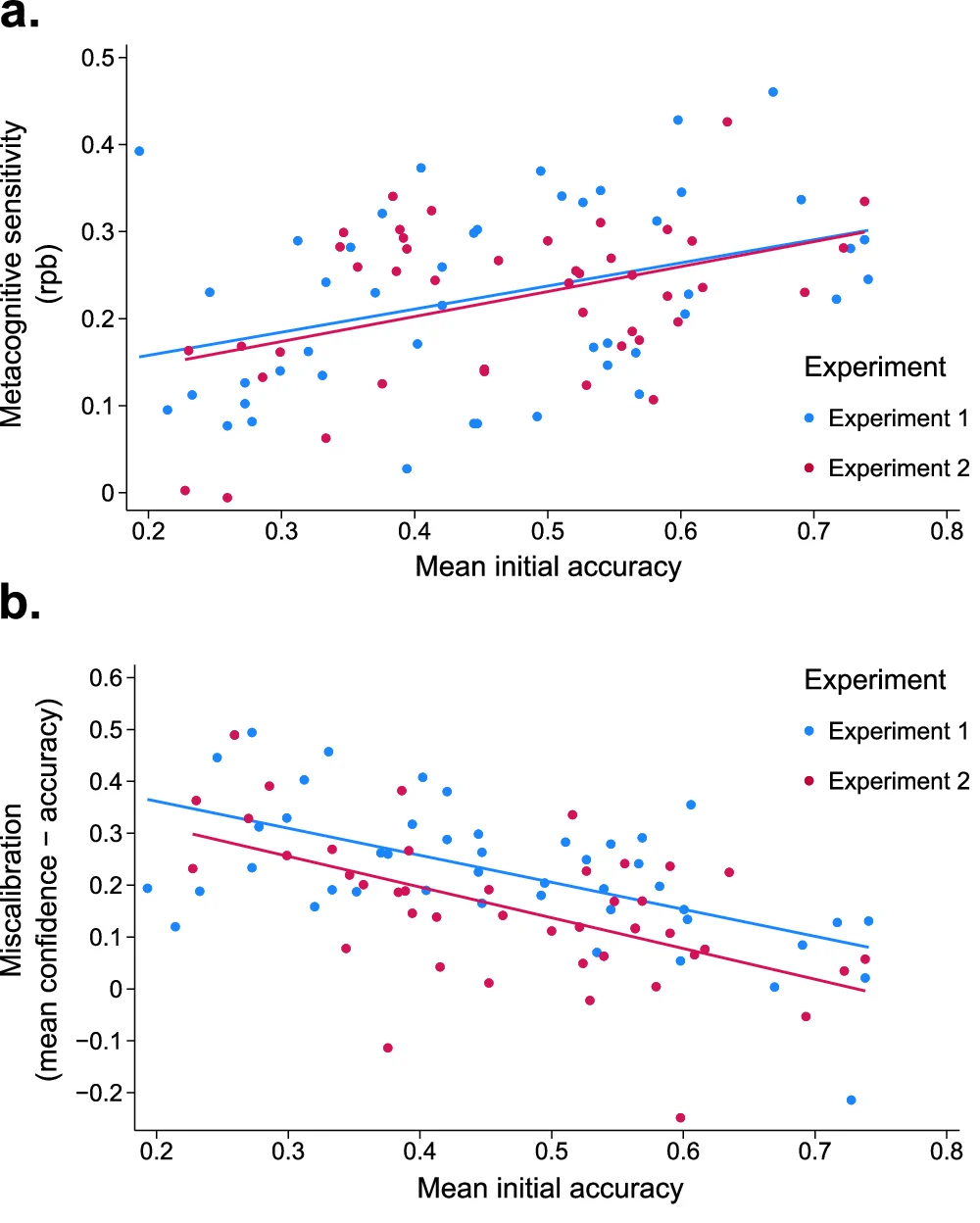
Accuracy and metacognition. **a** Scatterplot of mean initial accuracy and individual subject metacognitive sensitivity by experiment (*N* = 89). **b** Scatterplot of mean initial accuracy and miscalibration (i.e., overconfidence: mean confidence - accuracy) by experiment (*N* = 89).

Moreover, metacognitive sensitivity was positively associated with participants’ (initial) mean judgment accuracy (B = 0.28, SE = 0.07, t = 3.95, *p* < 0.001, 95% CI [0.14, 0.42]. Figure 12a), with no effect of treatment (B = 0.02, SE = 0.14, t = 0.15, *p* = 0.880, 95% CI [−0.26, 0.30]).

In turn, lower individual metacognitive sensitivity was associated with lower optimality in advice integration (B = 0.36, SE = 0.09, t = 3.95, *p* < 0.001, 95% CI [0.18, 0.55], Fig. 11b), with no significant difference across experiments (interaction: B = 0.16, SE = 0.18, t = 0.84, *p* = 0.402, 95% CI [−0.21, 0.52]). We investigated the drivers of this relationship by testing whether participants’ individual metacognitive sensitivity affected the use of relevant attributes (Model 3.2). Results (Supplementary Table S26) across the two experiments revealed that participants with low metacognitive sensitivity were more distant from the optimal benchmark in the use of trial-by-trial Δ *confidence* (Low CP - High CP: B = −0.06, SE = 0.02, t = −3.57, *p* < 0.001, 95% CI [−0.10, −0.03], Fig. 11c), and Δ *accuracy* (Low CP - High CP: B = −0.13, SE = 0.04, t = −3.50, *p* < 0.001, 95% CI [−0.20, −0.06]), compared to participants with high metacognitive sensitivity. Results from Model 3.3 (Supplementary Table S27) further clarified that the greater suboptimality in the use of Δ *confidence* among participants with low metacognitive sensitivity arose from insufficient adjustment to both their own and the adviser’s confidence (self: B = 0.06, SE = 0.03, t = 2.17, *p* = 0.03, 95% CI [0.01, 0.11]; adviser: B = −0.12, SE = 0.03, t = −4.42, *p* < 0.001, 95% CI [−0.17, −0.07]).

The relationship between metacognitive sensitivity and first-order task performance is well documented and theoretically expected (e.g., see the relationship between *d’* and meta-*d’*^43–47^). Consequently, between-subject variability in task accuracy could, in principle, explain the observed effects of metacognitive sensitivity. We therefore conducted three additional analyses to test (1) whether our results generalize to alternative measures of metacognitive ability and (2) whether the association between metacognitive sensitivity and optimality remains robust when controlling for shared variance with accuracy.

First, we tested the robustness of our metacognitive sensitivity measure (i.e, the point-biserial correlation) against an alternative measure based on different methodological assumptions: meta-*I*, a measure of mutual information derived from information theory^43^. The between-subject analyses using meta-*I* replicated the r_pb_ results: meta-*I* significantly predicted optimality (B = 0.88, SE = 0.28, t = 3.15, *p* = 0.002, 95% CI [0.32, 1.43]), with no evidence of differences across experiments (interaction: B = 0.31, SE = 0.56, t = 0.55, *p* = 0.585, 95% CI [−0.80, 1.41]). Second, we examined the relationship between metacognitive ability and optimality using metacognitive efficiency (specifically, meta-${I}_{2}^{r}$^43^) in place of our metacognitive sensitivity index (see SI, section “Alternative measures of metacognitive ability”). Meta-${I}_{2}^{r}$ is explicitly designed to isolate metacognitive sensitivity from its dependence on first-order performance. A regression model revealed that meta-${I}_{2}^{r}$ significantly predicted optimality (B = 0.73, SE = 0.26, t = 2.75, *p* = 0.007, 95% CI [0.20, 1.25]), with no significant interaction with treatment (B = 0.25, SE = 0.53, t = 0.47, *p* = 0.637, 95% CI [−0.80, 1.30]). Third, we estimated a regression model predicting optimality that included accuracy and its corresponding interaction terms alongside metacognitive sensitivity and treatment. Metacognitive sensitivity remained a significant predictor of optimality even after controlling for accuracy (B = 0.51, SE = 0.21, t = 2.45, *p* = 0.017, 95% CI [0.10, 0.93]), while the interaction with experiment remained non-significant (B = −0.26, SE = 0.42, t = −0.63, *p* = 0.529, 95% CI [−1.10, 0.57]).

Taken together, these results indicate that the relationship between metacognitive sensitivity and optimality cannot be accounted for by first-order task performance. Instead, they support the conclusion that the ability to generate sensitive metacognitive judgments uniquely contributes to more effective advice integration.

**Fig. 13:**
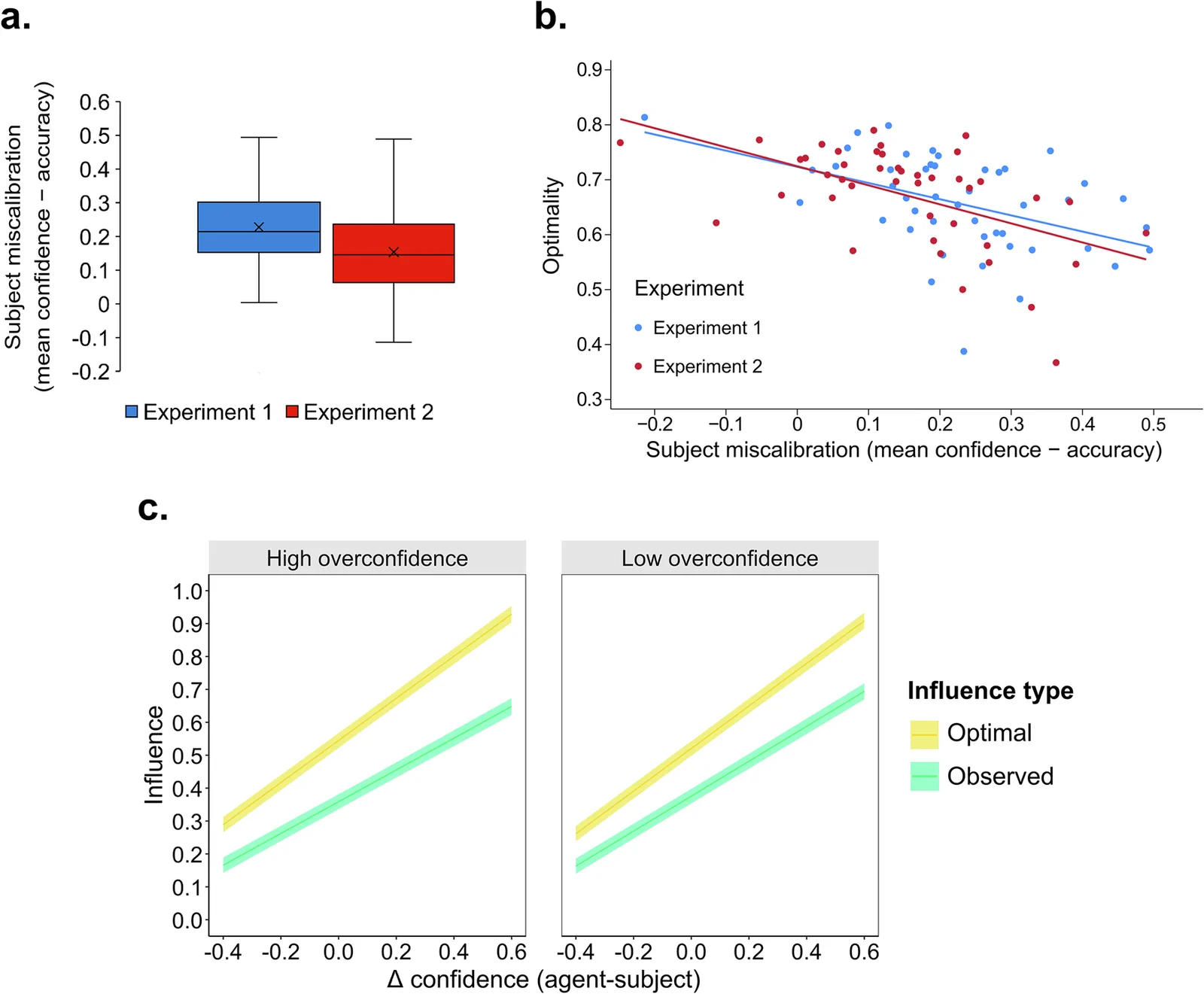
Confidence calibration and optimality. **a** Boxplots of participants’ miscalibration (mean confidence - accuracy) by experiment (*N* = 89). The box encompasses the IQR with the internal horizontal line representing the median and the ‘X’ marker indicating the arithmetic mean. Whiskers extend to the maximum and minimum values within 1.5*IQR. **b** Scatterplot of individual subject miscalibration and optimality, by experiment (*N* = 89). **c** Model 3.4 estimates (unstandardized betas) of participants’ influence as a function of Δ (normalized) trial-by-trial *confidence* (agent-subject) by *influence type* (observed in green, optimal in gold). The left panel refers to participants with high overconfidence, and the right panel to participants with low overconfidence, as defined by a median split at the individual level of miscalibration (mean confidence - accuracy). Ribbons express 95% confidence intervals of the regression estimates. Estimates based on data from 89 participants.

Despite receiving full feedback, participants in Experiment 1 exhibited marked positive miscalibration (i.e., overconfidence) in their confidence judgments (Fig. 13a). Mean confidence (M = 0.69, SD = 0.13) was significantly higher than mean accuracy in initial perceptual judgments made without advice (M = 0.46, SD = 0.15; Wilcoxon signed-rank test: W = 23, *p* < 0.001, 95% CI [−0.27, −0.19], r_rb_ = −0.96). Overconfidence persisted in Experiment 2, despite the disclosure of participants’ own mean confidence and accuracy (Confidence: M = 0.63, SD = 0.13; Accuracy: M = 0.47, SD = 0.13; Wilcoxon signed-rank test: W = 59, *p* < 0.001, 95% CI [−0.20, −0.11], r_rb_ = −0.88). However, we observed a significant treatment effect in Experiment 2: additional information reduced participants’ overconfidence, without eliminating it (Mann–Whitney U test on individual miscalibration: U = 669, *p* = 0.009, 95% CI [0.02, 0.13], r_rb_ = −0.32).

Higher levels of overconfidence were associated with lower initial accuracy (B = −0.56, SE = 0.09, t = −6.42, *p* < 0.001, 95% CI [−0.73, −0.38], Fig. 12b), with no effect of treatment (B = −0.07, SE = 0.17, t = −0.41, *p* = 0.681, 95% CI [−0.42, 0.27]). Importantly, individual overconfidence was associated with reduced optimality in advice integration (B = −0.32, SE = 0.06, t = −5.23, *p* < 0.001, 95% CI [−0.44, −0.20]; Fig. 13b), while the interaction with treatment is not significant (B = −0.05, SE = 0.12, t = −0.43, *p* = 0.672, 95% CI [−0.30, 0.19]). Results of Model 3.4 (Supplementary Table S28) showed that participants with higher overconfidence made less effective use of trial-by-trial Δ *confidence* (High - Low: B = −0.03, SE = 0.02, t = −1.98, *p* = 0.047, 95% CI [−0.07, −0.00], Fig. 13c), and Δ *accuracy* (High - Low: B = −0.16, SE = 0.04, t = −4.26, *p* < 0.001, 95% CI [−0.23, −0.08]), compared to less overconfident participants. Moreover, overconfident participants appeared to waste the advice more, showing stronger egocentric discounting (High - Low: B = −0.03, SE = 0.00, t = −6.26, *p* < 0.001, 95% CI [−0.04, −0.02]). Moreover, overconfident participants were less attracted by differences in mean confidence (Δ *mean confidence*, High - Low: B = −0.09, SE = 0.04, t = −1.97, *p* = 0.049, 95% CI [−0.17, −0.00]). Results from Model 3.5 (Supplementary Table S29) indicated that the greater suboptimality in the use of Δ *confidence* among overconfident participants stemmed from a specific insufficient adjustment to the *adviser’s* *confidence* (self: B = −0.04, SE = 0.03, t = −1.35, *p* = 0.178, 95% CI [−0.09, 0.02]; adviser: B = −0.13, SE = 0.03, t = −4.81, *p* < 0.001, 95% CI [−0.18, −0.08]).

**Fig. 14:**
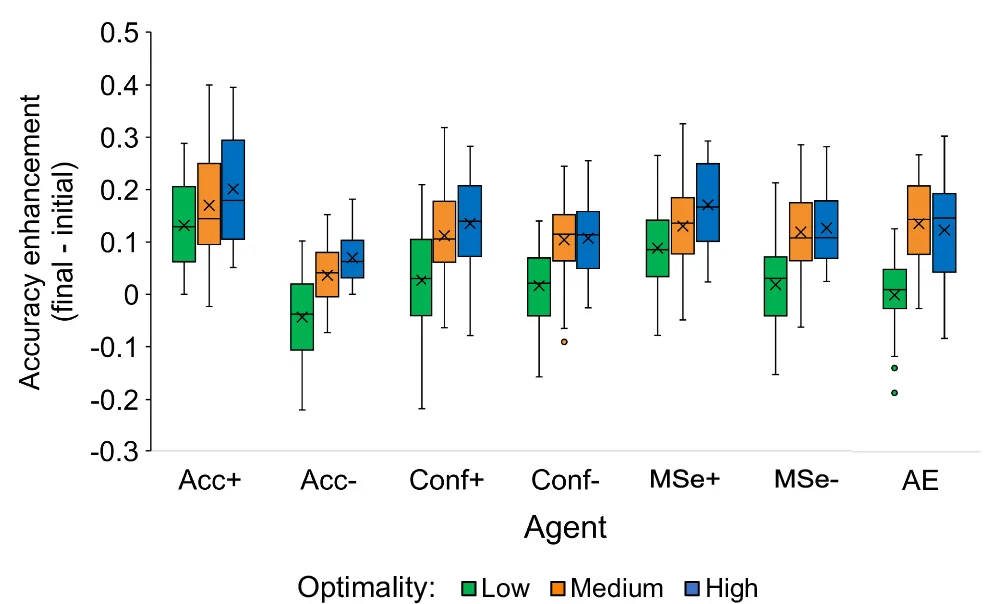
Individual optimality and accuracy enhancement. Boxplot of accuracy enhancement (final - initial accuracy) for tertiles of individual mean optimality (green: low; orange: medium; blue: high). The graph includes data from both experiments (*N* = 89). Tertiles were calculated based on ADT mean optimality, averaging across all experimental trials independently of agent type. Then we calculated the accuracy enhancement separately for the seven agent types. The box encompasses the IQR with the internal horizontal line representing the median and the ‘X’ marker indicating the arithmetic mean. Whiskers extend to the maximum and minimum values within 1.5*IQR. Individual points denote statistical outliers.

Overall, participants with stronger metacognitive abilities—reflected in better metacognitive sensitivity and calibration—achieved higher advice integration optimality. This inter-individual variability had important consequences for the effectiveness of advice (Fig. 14). The tertile with the lowest overall mean optimality showed negligible accuracy gains (0–2%) across most advisers, and even declined for the low-accuracy adviser (–4%). In contrast, the tertile with the highest overall mean optimality improved by an average of +13% relative to their initial decisions, enhancing accuracy across all advisers, including the low-accuracy one (+7%).

## Discussion

Human decision-making is fallible. One of the most effective strategies to mitigate this fallibility is the integration of external advice^51,52^. Advances in digital communication and artificial intelligence have greatly expanded access to high-quality advice, shifting the primary challenge from obtaining advice to integrating multiple sources optimally^4,5,9,49,53^. Our results showed that advice almost always improved participants’ initial performance, and in most conditions their advised decisions even outperformed the adviser. This generally strong performance by untrained participants reflected their spontaneous use of both their own and the adviser’s cognitive and metacognitive cues. However, comparison with the normative benchmark revealed that this modulation was quantitatively insufficient, resulting in suboptimal performance. Importantly, we also found substantial inter-individual differences in advice integration quality, systematically linked to participants’ metacognitive abilities.

### The half-full glass: sizable yet suboptimal improvements after advice

Although advice boosted performance, participants failed to realize its full potential, with an average of 13% (range: 2–28%) in accuracy gains left unrealized. Among the cognitive mechanisms outlined in the Introduction, insufficient information cannot account for this gap: participants received trial-by-trial feedback and confidence ratings, and in Experiment 2, they were explicitly informed of both their own and the adviser’s attributes. Selective attention to a subset of cues is also unlikely: participants modulated influence as a function of accuracy, mean confidence, metacognitive sensitivity, and local signals (judgments, trial-level confidence). Nor does failed adaptation fit the data: weighting strategies clearly adjusted to adviser characteristics.

The gap, therefore, points to systematic errors in information combination. Participants consistently placed more trust in their own judgments and confidence than in the adviser’s, even when no differences in accuracy or calibration justified such asymmetry. This egocentric advice discounting (EAD)^1,2,20,24,25,54,55^ was observed across all adviser types. Beyond replicating this well-established bias, our results extend previous work in two important ways. First, we show that EAD does not stem from erroneous estimates of relative competence (i.e., overplacement)^33,56^: participants displayed typical levels of discounting even when fully informed of both their own and the adviser’s attributes, including cases where the adviser was explicitly described as 20% more accurate. Importantly, EAD emerged even though trial-by-trial outcome feedback allowed participants to directly evaluate and revise any initial assumptions or skepticism about the adviser’s reliability. Second, we demonstrate that the bias extends to metacognitive cues: participants weighted their own confidence more heavily than the adviser’s, even when both signals were equally predictive. As a result, trial-by-trial fluctuations in self-confidence had a stronger impact on decisions than equivalent variations in the adviser’s confidence, explaining participants’ underutilization of trial-level confidence differences.

Nevertheless, egocentric discounting alone cannot explain all observed deviations. We identify a second source of suboptimality: a global-to-local integration deficit, defined as the difficulty of translating global knowledge of an adviser’s competence into consistent, trial-level adjustments. While egocentric discounting establishes a default influence below the rational 50% benchmark with symmetric partners, the global-to-local deficit prevents decision-makers from fully acting upon the trial-by-trial implications of a partner’s competence. This leads to a systematic under-modulation of advice weighting, a pattern that aligns with the classical anchoring-and-adjustment bias^57^ or, more recently, with behavioral attenuation^58^. The latter is a widespread phenomenon where the responsiveness of decisions to relevant evidence is insufficient due to information-processing constraints. Thus, while egocentric discounting acts as an intercept bias, behavioral attenuation blunts the slope of the response curve to diagnostic traits, causing decisions to regress toward a simplified, middle-ground default.

In sum, suboptimality did not result from a lack of information, selective attention, or failed adaptation. Instead, it originated from two integration errors: a default overweighting of self-generated judgments and a global-to-local deficit (a specific case of behavioral attenuation) in which diagnostic traits were weakly or inconsistently applied at the moment of decision.

Despite these systematic deviations from optimality, our findings also highlight the consistent benefits of advice. Final accuracy surpassed that of both individual judgments when advisers were similar or superior on any dimension, and only plateaued when advisers were markedly less accurate (≈20% lower), though participants still avoided performance decrements. These findings confirm a robust result from the social sciences: even imperfect advice typically enhances performance^6,8,13^. In addition, our work extends previous research along three key respects. First, we developed an exact quantitative measure of the suboptimality affecting participants’ judgment revision, lacking in previous studies. Second, we eliminated a limitation of earlier work by providing participants with complete diagnostic information, ensuring that suboptimality cannot be attributed to ignorance of the adviser’s behavior. Third, we systematically tested advice-taking across a wide range of adviser profiles, offering broader coverage of the variety of real-world interactions.

Taken together, these findings underscore a dual message. When provided with relevant information, individuals can flexibly use external input to improve performance; however, these gains are constrained by systematic integration errors that prevent them from reaping the full benefits of collaboration.

### Metacognition and inter-individual variability: a triple burden?

Consistent with previous literature^59–61^ participants displayed clear metacognitive limitations: they were markedly overconfident, and their trial-by-trial confidence only weakly predicted accuracy. Metacognitive competence was not only limited on average but also highly variable across individuals. In line with previous reports, lower first-judgment accuracy was associated with greater overconfidence and weaker metacognitive sensitivity. This pattern, referred to as the “double burden”^62,63^, may reflect a fundamental deficit in the quality of task-related signals available to less-skilled individuals^64,65^. Regardless of its cognitive origin, the double burden implies a compounding disadvantage: individuals with poorer initial performance also struggle to accurately assess their own limitations, creating a vicious cycle in which poor metacognition hinders recognition of the need for external advice.

Importantly, metacognitive ability also predicted the optimality of advice use. Individuals with weaker metacognition deviated more from normative integration, not because their signals were less informative (our Bayesian model accounted for this), but because they failed to use them effectively. They underweighted trial-by-trial confidence (especially the adviser’s) and failed to adequately adjust for the adviser’s accuracy, reinforcing a bias toward self-generated input. Taken together, these inter-individual dynamics may constitute a *triple burden*: individuals with low initial accuracy also tend to have poor metacognitive ability, which, in turn, is associated with a less effective use of external advice. This newly added disadvantage leaves them less able to compensate through external support.

### Practical considerations

Our findings provide a framework for characterizing the impact of advice in environments where judgment is augmented by external input, such as AI-assisted medical diagnosis^5,66–71^. We demonstrate that when responsibility remains with a single decision-maker and feedback is available, the optimal integration of advice outperforms simple delegation, even when the adviser’s accuracy exceeds that of the user.

Furthermore, the availability of a normative optimality benchmark provides a metric, the “advice gap,” to quantify performance loss relative to a theoretical optimum. This index allows for the precise quantification of the performance ceiling achievable through improved integration and provides a basis for comparing these potential gains against other sources of suboptimality. The presented Bayesian model can be adapted to various adviser characteristics and decision contexts to generate these estimates.

Finally, the wide inter-individual variability observed suggests that the expected benefits of expert or AI advice are modulated by the decision-maker’s integration efficiency. In cases where integration optimality is low, the accuracy gains provided by the adviser may be entirely offset. These results indicate that the success of advice-reliant systems depends on the efficiency of the integration process as much as on the accuracy of the advice itself.

### Limitations

The present study employed artificial agents with fixed, well-defined behavioral attributes, not conceived in terms of intentions and potential social orientations. This enabled us to isolate mechanisms of cognitive and metacognitive integration while abstracting from social factors such as intentionality, reputation-building, and strategic behavior. On the other hand, this approach restricts naive generalization to social interactions with human advisers.

Second, the task involved a perceptual decision-making paradigm with explicit feedback and quantifiable uncertainty. Even though this warrants precise modeling of optimal integration, advice use in more complex, ambiguous, or feedback-poor domains may rely on partially different mechanisms.

Third, our Bayesian model provides a normative benchmark conditional on the information available to participants, but it does not capture potential cognitive costs, heuristic constraints, or representational limits that may shape real-world decision strategies. Future work should examine how such constraints interact with advice integration and provide a *descriptive* model of the integration process.

## Conclusions

As advice becomes ever more abundant, the challenge shifts from access to effective use. In information-rich environments, people benefit from most advisers, yet they fall short of optimal benchmarks due to persistent egocentric bias and difficulties in translating global knowledge into decision-level adjustments. These gaps are not evenly distributed: they are most pronounced in individuals with lower competence, thereby compounding disadvantage. Beyond human–human settings, these insights are particularly relevant for human–AI interaction, where the quality of advice and metacognitive cues can vary widely^72^. By quantifying the gap between observed and optimal advice use, our study identifies concrete targets for training and intervention, paving the way toward more reliable and effective assisted decision-making.

## Supplementary information

Transparent Peer Review file

Supplementary Information file

## Data Availability

The data required to reproduce the results and figures presented in this paper are publicly available in a dedicated Open Science Framework (OSF) repository at https://osf.io/5ekdb/.

## Code availability

The complete Jamovi analysis files (.omv), containing the raw data, statistical parameters, and analysis scripts required to reproduce the main statistical analyses presented in this paper, are publicly available via the Open Science Framework (OSF) at https://osf.io/5ekdb/.
