# Supplementary material for: Adaptive yet suboptimal integration of advice in decision-making
Source: Commun Psychol. 2026 May 16;4:123. doi: 10.1038/s44271-026-00456-1 (PMC13547347; doi:10.1038/s44271-026-00456-1)
Supplement: Supplementary file 1 [file 44271_2026_456_MOESM1_ESM.pdf]

nature portfolio

# Peer Review File

# Adaptive yet suboptimal integration of advice in decision making

Corresponding Author: Dr Joshua Zonca

**This file contains all editorial decision letters in order by version, followed by all author rebuttals in order by version.**

Version 0:

Decision Letter:

** Please ensure you delete the link to your author homepage in this e-mail if you wish to forward it to your coauthors **

Dear Dr Zonca,

Thank you for your patience during the peer-review process. Your manuscript titled "The advice gap: adaptive yet suboptimal advice integration" has now been seen by 4 reviewers, and I include their comments at the end of this message. They find your work of interest but raised some important points. We are interested in the possibility of publishing your study in Communications Psychology, but would like to consider your responses to these concerns and assess a revised manuscript before we make a final decision on publication.

We therefore invite you to revise and resubmit your manuscript, along with a point-by-point response to the reviewers. Please highlight all changes in the manuscript text file.

Editorially, we consider particularly important that you address the concerns raised about the baseline of decision revision without advice (R#1 & 3), about the recency effects regarding the integration of advice (R#2 and R#4) and the learning dimension (R#1-4), and that you clarify/discuss how you work integrate with the several relevant strand of literature identified by the reviewers (R#2, R#4).

Please also provide a sensitivity power analysis that is based on the smallest effect size of theoretical or pragmatic interest (SESOI) of all hypotheses. We require a scientific justification of what makes the specific target effect size the smallest effect size of interest. This should be informed by comparable effect sizes in the field of study (but not single studies).

As you revise the manuscript in response to these issues, please also implement all requests in the attached Mandatory Revision Requests document. All requirements listed in this document need to be fully met, or the work will be returned to you for further revisions without peer review. This workflow is in place to increase the likelihood that the paper will be accepted for publication. It reduces the number of rounds of revision (and review) and ensures that the reviewers vet a version of the article that is compliant with journal policies. If you have any questions regarding the required revisions, please contact the journal prior to resubmission to avoid a negative outcome.

We are committed to providing a fair and constructive peer-review process. Please don't hesitate to contact us if you wish to discuss the revision in more detail.

Please submit the following items:

- Revised manuscript
- Point-by-point response to the referees' comments
- Mandatory Revision Requests Table (attached).
- Cover letter (as a separate document)

via this link: Link Redacted .

** This url links to your confidential home page and associated information about manuscripts you may have submitted or are reviewing for us. If you wish to forward this email to co-authors, please delete the link to your homepage first **

We hope to receive your revised paper within 8 weeks; please let us know if you aren't able to submit it within this time so that we can discuss how best to proceed. If we don't hear from you, and the revision process takes significantly longer, we may close your file. In this event, we will still be happy to reconsider your paper at a later date, provided it still presents a significant contribution to the literature at that stage.

We look forward to seeing the revised manuscript and thank you for the opportunity to review your work.

Best regards,

Troby Lui, on behalf of

Mael Lebreton

Troby Lui, PhD
Associate Editor
Communications Psychology

Mael Lebreton, PhD
Editorial Board Member
Communications Psychology
orcid.org/0000-0002-2071-4890

REVIEWER EXPERTISE:
Reviewer #1: metacognition
Reviewer #2: metacognition, advice
Reviewer #3: metacognition
Reviewer #4: metacognition, advice

REVIEWER REPORTS:

Reviewer #1 (Remarks to the Author):

The paper by Zonca et al. investigates how humans integrate advice in decision-making contexts, using a Bayesian model simulation as a benchmark to quantify how humans deviate from optimality. The authors conducted two experiments using a paradigm in which subject first made their own decision and then were given the advisor's decision and confidence, allowing them to calibrate their decision. There are seven blocks of trials, and in each block the advisor's attributes vary in one dimension: either accuracy, confidence, or predictability of confidence (confidence accuracy correlation). They also included a block where the advisor was comparable to the participant along all attributes. In Experiment 1 subjects were not informed of the advisors' attributes and in Experiment 2 they were informed. The authors found that while advice generally improves performance, the advice integration remains systematically suboptimal compared to the Bayesian optimality benchmark. This topic is interesting and the writing is generally clear, but I do have some theoretical concerns regarding the conclusion they draw as well as some questions for the results and methods.

Major comments
1. For matched-accuracy advisers, the adviser isn't informationally superior, but subjects still showed an increase in their accuracy. While this is mathematically possible, as they just average two noisy sample from the same distribution and the accuracy might improve, I wonder whether the observed accuracy boost could be at least partly driven by the fact that they were just given a second chance to adjust their initial judgement (Resulaj et al., 2009). One potential way to look into this is, perhaps, by analyzing trials where agent's estimate closely matches the subject's initial estimate. If accuracy improves even when advisers provide minimal new information, this would suggest that revision opportunity effects exist.
2. The Bayesian model functions as an ideal observer with perfect knowledge of task statistics, while human participants receive no explicit information about these statistics and face rapidly changing conditions (every 54 trials) in Experiment 1. Given that in studies that examine learning, human typically requires much more trials to learn (Behrens et al., 2007); here, participants were not given the opportunity to achieve something even close to the author's definition of "optimality". In this context, relying more on confident advisers represents a reasonable heuristic. Also, it might be helpful for the authors to analyze learning within blocks (first vs. second half) to assess adaptation, though 54 trials likely provide insufficient learning opportunity. Anyhow, the conclusions regarding "suboptimality" in Experiment 1 should be toned down.
3. In the introduction, the authors identified five sources of suboptimality, but they appear to overlap with each other conceptually. For example, "Integration errors" (combining information non-optimally) subsumes "failed adaptation" (not

adjusting to adviser characteristics), as the latter represents a specific type of integration error. Similarly, "selective consideration" essentially describes suboptimal evidence weighting, another form of integration error. The authors might consider clarifying the distinctions.

Minor comments
- The correct response is defined as an "accuracy range" within 10 degrees, but how was this range determined?
- For accessing metacognitive skills, why use correlation rather than established measures like meta-d' and M-Ratio?
- The authors might consider reporting whether randomization/counterbalancing was used for adviser order, as order effects could confound results.
- Can stimuli appear at any direction? If yes, how is the random guess rate calculated for continuous angular space? The "R" marker (indicating random guess reference line to subjects) explanation to participants should also be detailed.
- The authors start introducing modeling results without clearly explaining what these models' variants are, which makes it really hard to follow.
- Are these typos in SI? Seems like it should be "Model 1.2" instead of "Model 1,2". Similarly, for "Model 2,1", "Model 2,2".
- Table numbering appears incorrect (S1-2 referenced after S3-4).
- Why only excluding the first third of each block for model 1.3 to account for initial learning? Why is such exclusion not applied for model 1.1 and 1.2? This seems inconsistent. Also, this number (1/3) seems arbitrary.
- Figure 7 as an instruction seem too complex for subjects to interpret, such that understanding distributions and differential confidence for correct/incorrect trials requires some knowledge about stats/math. Were participants given additional instruction?
- The description of the polynomial mapping from mean accuracy to SD is not clear. In line 842-843 "The conversion followed a polynomial function with parameters [–358.5177 621.9958 –361.1622 81.3906]", these parameters seem to be the coefficients of a 3rd-order polynomial fit approximating the inverse of the function that maps SD to accuracy as there is no closed-form solution? However, this is not explained at all, nor did they explicitly state the analytic form of the underlying accuracy -> SD relationship they used.
- The authors used an iterative procedure to generate advisers' trial-by-trial behavior (page 33), and the tolerance threshold of this procedure progressively increased across iterations (up to 0.2). A tolerance of 0.2 on the scale of 0-1 is actually large, and thus the advisor behavior simulated might differ from intended. It might be clearer to report how often the algorithm had to rely on high tolerance values and the distribution of realized advisor attributes relative to target values at each participant x block level.
- In the current design, subjects provide confidence rating after their initial judgment and are not allowed to revise the confidence rating after seeing the adviser's estimate and confidence. This simplifies the modeling, but it would be interesting if subjects are allowed to change their confidence ratings to learn about metacognitive updating. They might change their confidence even when they keep or only slightly change their initial judgment.

Reviewer #2 (Remarks to the Author):

Comments on "The advice gap: adaptive yet suboptimal advice integration"
In this paper the authors examined the way participants integrate advice in a perceptual decision-making task. They manipulated the advisers accuracy, confidence and calibration, and examine how participants adapted their integration policy in the different conditions. This was done once with no explicit description of the advisers, and participants needed to learn advisers properties from experience, and once with full details available to participants. Finally, the authors generated optimal decisions based on a Bayesian integration model, which served as a benchmark for participants' optimal decisions. The authors found that participants generally benefitted from advice, and were able to adapt their integration based on advisers' accuracy and calibration. However, participants over-relied on advisers' confidence. Overall participants advice integration was suboptimal, when compared with the optimal model. Explicit information did not shift advice taking behavior. Finally, individual metacognitive abilities were related to advice taking, as participants with more calibrated confidence were more optimal in their advice integration.
This is a carefully designed worked, with interesting findings. Some findings were already reported before, such as egocentric bias and reliance on confidence for advice integration. However, some other manipulations are novel, and the comparison of explicit and implicit description of advisers abilities is interesting. It was easy to read the paper, results are clear and methods are detailed. I appreciate the data availability, analysis code would be even better.
Comments
1. The framing of advice is not well defined in this paper. To the best of my understanding, advice was presented as virtual agent's advice, but no clear details about what participants thought was the source of advice, the intentions of agents, or their variabilities is described.
2. More broadly, this paper could be better situated within the literature on social learning. Advice is usually associated with agents intentionally sharing information in order to influence other's behavior (Hertz, Bell, and Raihani 2021), with some consequences for adviser (Anlló et al. 2024). It is different from observation or imitation, where there is no intentionality in sharing information (Najar et al. 2020). In addition, crowds can provide information (Yonah and Kessler 2021). These framings, especially advice, have profound effect on advice taking, usually leading to over compliance (Pereg et al. 2024) or to normative pressures (Mahmoodi et al. 2022, 2015). Can the author elaborate on how this work fits within these ideas?
3. There seem to be a bias towards confidence as a signal of accuracy. This seems to be a relatively reliable heuristics, as in many real world scenarios this is the case. I think that this is the point of works from Bang, Bahrami and Flemming. I am not sure how your findings fit-in with their work.
4. It may be that participants show egocentric bias because advice appears after their own answer is presented – what if this was not the case, and they would see the advice before making their own decision? See (Hertz et al. 2016; Nafcha and Hertz 2022). It may be that anchoring on one's own advice is the root of bias in this work as well, and that tweaking the

design may allow increased optimality.
5. Do you think that the Bayesian model may have assumptions that are overoptimistic about human cognition, e.g., perfect estimation of sensory noise, perfect memory, unbiased integration of trial-by-trial confidence and accuracy, internal representation of noise and so on? There are currently works on resource-rational models of bounded computational cognitive capacity which may indicate some reasons for non-optimality here.
6. Technical point – was there ever a negative influence? How do you measure this?
7. Accuracy effects – when an adviser is more accurate, it also means less distance from participant, compared with less accurate advisors. This leaves less space for improvement and change after viewing the advice. Did it affect influence?
8. In model 1.4 you report mean influence of 0.36 – why is this an egocentric bias? What would be the expected influence for no bias? I guess it should be 0.5, right? So is 0.36 really lower than 0.5? you compare it in the model to 0 in the model, right?
9. How was 'initial mean accuracy' defined? I could not find the definition.
10. Estimation of learning – did you fit a mode estimating participants trial-by-trial learning about the advisers' accuracy, confidence and calibration? Can participant learn about these? Experience with variance in advisors accuracy and confidence may lead to egocentric bias. While explicit information mitigates some of the learning issue, it may also be that participants find it hard to understand some features (confidence, calibration) while accuracy is easier to understand.

Anlló, Hernán, Gil Salamander, Nichola Raihani, Stefano Palminteri, and Uri Hertz. 2024. "Experience and Advice Consequences Shape Information Sharing Strategies." Communications Psychology 2 (1): 1–13.
Hertz, Uri, Vaughan Bell, and Nichola Raihani. 2021. "Trusting and Learning from Others: Immediate and Long-Term Effects of Learning from Observation and Advice." Proceedings. Biological Sciences / The Royal Society 288 (1961): 20211414.
Hertz, Uri, Margaux Romand-Monnier, Konstantina Kyriakopoulou, and Bahador Bahrami. 2016. "Social Influence Protects Collective Decision Making from Equality Bias." Journal of Experimental Psychology. Human Perception and Performance 42 (2): 164–72.
Mahmoodi, Ali, Dan Bang, Karsten Olsen, Yuanyuan Aimee Zhao, Zhenhao Shi, Kristina Broberg, Shervin Safavi, et al. 2015. "Equality Bias Impairs Collective Decision-Making across Cultures." Proceedings of the National Academy of Sciences 112 (12): 3835–40.
Mahmoodi, Ali, Hamed Nili, Dan Bang, Carsten Mehring, and Bahador Bahrami. 2022. "Distinct Neurocomputational Mechanisms Support Informational and Socially Normative Conformity." PLoS Biology 20 (3): e3001565.
Nafcha, Orit, and Uri Hertz. 2022. "The Devil Is in the Task Structure." ELife 11 (April): e78930.
Najar, Anis, Emmanuelle Bonnet, Bahador Bahrami, and Stefano Palminteri. 2020. "The Actions of Others Act as a Pseudo-Reward to Drive Imitation in the Context of Social Reinforcement Learning." Edited by Matthew F. S. Rushworth. PLoS Biology 18 (12): e3001028.
Pereg, Maayan, Uri Hertz, Ido Ben-Artzi, and Nitzan Shahar. 2024. "Disentangling the Contribution of Individual and Social Learning Processes in Human Advice-Taking Behavior." NPJ Science of Learning 9 (1): 4.
Yonah, Merav, and Yoav Kessler. 2021. "'they Don't Know Better than I Do': People Prefer Seeing for Themselves over Using the Wisdom of Crowds in Perceptual Decision Making." Journal of Cognition 4 (1): 30.

Reviewer #3 (Remarks to the Author):

I co-reviewed this manuscript with one of the reviewers who provided the listed reports. This is part of the Communications Psychology initiative to facilitate training in peer review and to provide appropriate recognition for Early Career Researchers who co-review manuscripts.

Reviewer #4 (Remarks to the Author):

I greatly enjoyed reading and reviewing this manuscript by Zonca and colleagues. This is really interesting and well-written work. I also appreciate that the study was pre-registered.
Overall, the manuscript tackles an important question: how people integrate external advice, how their own metacognitive ability influences this integration, and whether they take into account another advisor's accuracy and metacognition. The study design is thoughtful, and the attempt to benchmark human performance against an optimal model is commendable. However, substantial clarifications, deeper engagement with the existing literature, and some additional analyses are required before the manuscript is suitable for publication. I provide these comments in a constructive spirit: with revisions, the paper has the potential to make a really valuable contribution to the field.

Major Comments

1. Confidence Predictivity: definition, timing of introduction, and conceptual alignment
On page 3, the manuscript refers to "confidence predictivity" without explaining what this is until much later. This explanation should come earlier.
More importantly, confidence predictivity—operationalised as a correlation between error and confidence—is no longer the standard way the metacognition literature captures insight or metacognitive sensitivity. While I can see why this measure may have seemed practical in this task (particularly given the continuous, angular error measures), it is well established that such correlations are sensitive to first-order performance (e.g., Maniscalco & Lau, 2012; Fleming & Lau, 2014). This issue requires explicit discussion.

I appreciate that the advisors were already constructed based on this measure, and I am not suggesting the authors rewrite the entire experimental logic or run any additional experiments. However, additional post-hoc analyses using standard metacognitive metrics would greatly strengthen the manuscript. Meta-I(-ratio) seems most appropriate given varying difficulty; this can be easily calculated using the R toolbox statConfR (c.f. Dayan, 2023). These analyses would help demonstrate that the effects do not hinge solely on this potentially biased operationalisation.
Furthermore, the term "confidence predictivity" is rare in the literature. It would be more consistent to refer to it as something similar to a phi coefficient to avoid proliferating new terminology or "toothbrushes"—keeping with the tongue-in-cheek metaphor by Elson et al. (2023).

2. Missing discussion of trust and suspected deception
The manuscript only briefly mentions trust, which is a central variable in advice-taking research (e.g., Pescetelli & Yeung, 2020). People might distrust computer-generated or experimenter-generated advice, especially when advice is personalised or appears "too good." From my own anecdotal experience, participants often suspect deception throughout advice studies. My concern is that the egocentric advice discounting observed here may partly reflect participants hedging against potentially malicious or unreliable advice, rather than a principled integration deficit. This should be explicitly discussed.
Relatedly: Were participants debriefed? Did any report suspicion about the advisors? This information is essential for interpreting the findings.
Moreover, the task environment is highly artificial. This is not a criticism per se—such paradigms are extremely useful precisely because they isolate integration mechanisms from real-world social influences—but the limitations for generalising to naturalistic advice-taking require deeper discussion.

3. Benchmarking against an optimal model
A positive and valuable aspect of the manuscript is the explicit benchmarking of human performance against an optimal solution. This is useful for the field and the authors should keep this element prominent. However, this paper is by no means the first or only one to advocate such an approach, and additional relevant research should be cited and discussed. The authors already cite one such study (Bahrami, Olsen, Latham, Roepstorff, Rees, & Frith, 2010). I did a quick search and found Pescetelli, Hauperich, & Yeung (2021), letting me suspect there are likely other studies that have attempted this.

4. Learning over time
Was the task long enough for participants to actually learn or infer advisor quality? See, for instance, Carlebach & Yeung (2023) for work showing how confidence plays a key role in this gradual process.
One way to test this directly is to split the data into first versus second halves of the experiment and examine whether advice integration increases over time. This may also relate to trust-building (see above). This would of course be an additional, exploratory (and potentially slightly underpowered) analysis that would go beyond your preregistered analysis plan. I do believe, however, that it will be highly useful for readers to understand any such potential trends in your data.
This point is also linked to the phenomenon described as a global-to-local integration deficit, which is fascinating but appears a little underdeveloped. Page 22 would benefit from unpacking this idea in more detail. Have there been parallel findings in other research domains? How should a participant know the global characteristics of an advisor in Experiment 1 at the beginning of a block? Is this truly an integration deficit or simply learning in progress?

5. Missing literature
As pointed out above, several important strands of literature are absent and should be integrated to ground the manuscript more firmly. In addition to papers mentioned above, one relevant missing study is Hertz, Palminteri, Brunetti, Olesen, Frith, & Bahrami (2017). Other relevant pockets of literature include work on information search and confidence (e.g., Steve Fleming's lab; Kobe Desender; Nick Yeung). This is intended as constructive criticism to further anchor the study in the existing literature.

6. Preregistration inconsistencies
It is excellent that both studies were carefully preregistered. However, the section in the supplement where the authors list deviations from the preregistrations requires considerably more detail.
Relatedly, after briefly skimming the preregistrations, I noticed several preregistered tests missing from the manuscript (e.g., RT analyses, control analyses). On page 19 of the supplement, the authors state that "a few exploratory analyses were not reported in the final manuscript." But these analyses appear to have been preregistered and thus cannot be omitted.

7. Bayesian model fitting
I am not familiar with the GeNIe toolbox, so apologies if I am overlooking something. That said, I found myself wondering how we can be confident that the Bayesian model used to generate the optimal final decisions was indeed the most appropriate model for this task. At present, the manuscript does not report any formal model comparison, fitted parameters, parameter recovery, or model confusion analyses. These checks are typically essential for demonstrating that a model both fits the data well and is distinguishable from plausible alternatives. If these analyses were conducted, it is necessary to include them; if not, I think it would be important to acknowledge this as a limitation.

Minor Comments
1. The finding that participants weighted advisor confidence more heavily than advisor accuracy may have a simple explanation: advisor confidence is presented before feedback, whereas accuracy only becomes clear after feedback. In a complex task with many information streams, primacy effects are highly plausible. Please discuss this alternative interpretation.
2. Please clarify that Model 1.4 refers to a mixed-effects model and briefly outline what this model predicts and which predictors it includes. It took me until I read the supplement to understand that this was not a variant of the Bayesian ideal

observer model.
3. I may have missed this, but I am still unsure why the Pre+ / Pre– agents were not defined relative to each participant.
4. In Figure 1, the small number shown on screen (e.g., blue “49”, red “76”) is ambiguous. Is this the stimulus angle or the adviser’s confidence rating? This could be more clearly explained in the caption and main text.
5. When constructing personalised advisors based on participants’ own accuracy and metacognition, how did the authors address cases where participants were at ceiling or bottom for accuracy/confidence/resolution? Some descriptive statistics on the distributions of variables such as sMA/sMC/sCR and aMA/aMC/aCR would be very helpful. Apologies if I missed this information somehow.
6. Please include which software was used to present the stimuli. Was testing carried out in-person or online?
7. Figure 12 is intriguing, but one key point requires clarification: are the tertiles created across all conditions and kept constant? In other words, are the participants included in the green bar (ACT+) the same participants included in the green bar (ACT–)? Or are these averages computed across differing subsets? This affects how optimality should be interpreted.
8. The way accuracy and confidence were presented in Experiment 2 may not be ecologically valid. Prior work (e.g., Gigerenzer and colleagues) suggests that format strongly affects cognitive processes, in this case information integration. The authors should discuss whether the presentation format may explain the weaker advice integration in Experiment 2.
9. At the bottom of page 2, the phrasing can be misunderstood to mean that relative differences between advisors’ cognitive/metacognitive skills were manipulated. In reality, this is the delta between participant and agent—please clarify.
10. On page 4 the minus sign is missing for the Pre– agent.

Referenced Literature
Carlebach, N., & Yeung, N. (2023). Flexible use of confidence to guide advice requests. Cognition, 230, 105264. https://doi.org/10.1016/j.cognition.2022.105264
Dayan, P. (2023). Metacognitive Information Theory. Open Mind, 7, 392–411. https://doi.org/10.1162/opmi_a_00091
Elson, M., Hussey, I., Alsalti, T., & Arslan, R. C. (2023). Psychological measures aren’t toothbrushes. Communications Psychology, 1(1). https://doi.org/10.1038/s44271-023-00026-9
Fleming, S. M., & Lau, H. C. (2014). How to measure metacognition. Frontiers in Human Neuroscience, 8(July), 443. https://doi.org/10.3389/fnhum.2014.00443
Hertz, U., Palminteri, S., Brunetti, S., Olesen, C., Frith, C. D., & Bahrami, B. (2017). Neural computations underpinning the strategic management of influence in advice giving. Nature Communications, 8(1). https://doi.org/10.1038/s41467-017-02314-5
Maniscalco, B., & Lau, H. (2012). A signal detection theoretic approach for estimating metacognitive sensitivity from confidence ratings. Consciousness and Cognition, 21(1), 422–430. https://doi.org/10.1016/j.concog.2011.09.021
Pescetelli, N., & Yeung, N. (2021). The role of decision confidence in advice-taking and trust formation. Journal of Experimental Psychology: General, 150(3), 507–526. https://doi.org/10.1037/xge0000960
Pescetelli, N., Hauperich, A. K., & Yeung, N. (2021). Confidence, advice seeking and changes of mind in decision making. Cognition, 215(October 2020). https://doi.org/10.1016/j.cognition.2021.104810

* TRANSPARENT PEER REVIEW: Communications Psychology uses a transparent peer review system. This means that we publish the editorial decision letters including Reviewers' comments to the authors and the author rebuttal letters online as a supplementary peer review file. However, on author request, confidential information and data can be removed from the published reviewer reports and rebuttal letters prior to publication. If your manuscript has been previously reviewed at another journal, those Reviewers' comments would not form part of the published peer review file.

** Visit Nature Research's author and referees' website at <a href="http://www.nature.com/authors">www.nature.com/authors</a> for information about policies, services and author benefits**

Communications Psychology is committed to improving transparency in authorship. As part of our efforts in this direction, we are now requesting that all authors identified as ‘corresponding author’ create and link their Open Researcher and Contributor Identifier (ORCID) with their account on the Manuscript Tracking System prior to acceptance. ORCID helps the scientific community achieve unambiguous attribution of all scholarly contributions. You can create and link your ORCID from the home page of the Manuscript Tracking System by clicking on ‘Modify my Springer Nature account’ and following the instructions in the link below. Please also inform all co-authors that they can add their ORCIDs to their accounts and that they must do so prior to acceptance.
https://www.springernature.com/gp/researchers/orcid/orcid-for-nature-research

For more information please visit http://www.springernature.com/orcid

If you experience problems in linking your ORCID, please contact the <a href="http://platformsupport.nature.com/">Platform Support Helpdesk</a>.

Version 1:

Decision Letter:

** Please ensure you delete the link to your author homepage in this e-mail if you wish to forward it to your coauthors **

Dear Dr Zonca,

Your manuscript titled "Adaptive yet suboptimal integration of advice in decision making" has now been seen by our reviewers, whose comments appear below. In light of their advice I am delighted to say that we are happy, in principle, to publish a suitably revised version in Communications Psychology.

We therefore invite you to revise your paper one last time to address the remaining concerns of our reviewers and a list of editorial requests. At the same time we ask that you edit your manuscript to comply with our format requirements and to maximise the accessibility and therefore the impact of your work.

EDITORIAL REQUESTS:

Please review our specific editorial comments and requests regarding your manuscript in the attached "Editorial Requests Table". Please outline your response to each request in the right hand column. Please upload the completed table with your manuscript files as a Related Manuscript file.

If you have any questions or concerns about any of our requests, please do not hesitate to contact me.

SUBMISSION INFORMATION:

In order to accept your paper, we require the files listed here https://www.nature.com/documents/commsj-file-checklist.pdf .

OPEN ACCESS:

Communications Psychology is a fully open access journal. Articles are made freely accessible on publication. For further information about article processing charges, open access funding, and advice and support from Nature Research, please visit https://www.nature.com/commspsychol/open-access

At acceptance, you will be provided with instructions for completing the open access licence agreement on behalf of all authors. This grants us the necessary permissions to publish your paper. Additionally, you will be asked to declare that all required third party permissions have been obtained, and to provide billing information in order to pay the article-processing charge (APC).

* TRANSPARENT PEER REVIEW: Communications Psychology uses a transparent peer review system. On author request, confidential information and data can be removed from the published reviewer reports and rebuttal letters prior to publication. If you are concerned about the release of confidential data, please let us know specifically what information you would like to have removed. Please note that we cannot incorporate redactions for any other reasons.

* CODE AVAILABILITY: All Communications Psychology manuscripts must include a section titled "Code Availability" at the end of the methods section. We require that the custom analysis code supporting your conclusions is made available in a publicly accessible repository at this stage; please choose a repository that generates a digital object identifier (DOI) for the code; the link to the repository and the DOI must be included in the Code Availability statement. Publication as Supplementary Information will not suffice.

* DATA AVAILABILITY:
All Communications Psychology manuscripts must include a section titled "Data Availability" at the end of the Methods section. More information on this policy, is available in the Editorial Requests Table and at <a href="http://www.nature.com/authors/policies/data/data-availability-statements-data-citations.pdf">http://www.nature.com/authors/policies/data/data-availability-statements-data-citations.pdf</a>.

Please use the following link to submit the above items:
Link Redacted
** This url links to your confidential home page and associated information about manuscripts you may have submitted or be reviewing for us. If you wish to forward this email to co-authors, please delete the link to your homepage first **

We hope to hear from you within two weeks; please let us know if you need more time.

Best regards,

Troby Lui, on behalf of

Mael Lebreton

Troby Lui, PhD
Associate Editor
Communications Psychology

Mael Lebreton, PhD
Editorial Board Member
Communications Psychology
orcid.org/0000-0002-2071-4890

REVIEWERS' COMMENTS:

Reviewer #1 (Remarks to the Author):

I thank the authors for the thorough revisions. My concerns have all been addressed and the manuscript has improved substantially. I have only one remaining minor comment: in the rebuttal the authors explain that the ±10° accuracy window was chosen based on pilot studies, but I wasn't able to find that in the Methods. I suggest adding this detail to the Methods explicitly.

Reviewer #2 (Remarks to the Author):

I appreciate the authors replies and additional analyses addressing my comments and questions. I have no further comments.

Reviewer #3 (Remarks to the Author):

I co-reviewed this manuscript with one of the reviewers who provided the listed reports. This is part of the Communications Psychology initiative to facilitate training in peer review and to provide appropriate recognition for Early Career Researchers who co-review manuscripts.

Reviewer #4 (Remarks to the Author):

The authors provided thorough and thoughtful revisions of their manuscript. Overall, I believe the manuscript has improved significantly.

In particular, I think the revisions regarding the measurement of metacognition have been handled well. The authors now define their central measure of 'confidence predictivity' early in the manuscript, clearly link it to established metacognitive measures, and provide additional analyses in the supplement using more standard indices. The manuscript is now much more firmly embedded in the metacognition literature (and I appreciate that it sits in-between different fields). This was a central concern in my previous review, and I think the authors have done a good job addressing it.

I still feel a bit uneasy about the decision to introduce the term 'confidence predictivity', as this is not the standard label for this type of measure. My concern is primarily about potential label proliferation in a literature that already contains many overlapping constructs. That said, I recognise that the construct is now clearly defined and linked to established measures, and I do not wish to insist further on this point, especially as it does not appear to have concerned the other reviewers to the same extent.

1) On claims regarding optimality
I still disagree with the sentence stating that "few studies have attempted to formally quantify (sub)optimality." As the authors themselves acknowledge, there is already a substantial body of work benchmarking behaviour against normative or optimal standards in this line of research. While I appreciate that additional relevant studies have now been cited, I would encourage the authors to further tone down this statement to avoid overstating the novelty of this aspect of their approach.

2) On the new learning analysis and the global-to-local integration deficit
I think the newly added learning analysis is a valuable addition to the manuscript.
However, I believe the second part of my previous comment regarding the global-to-local integration deficit has not been fully addressed. I would still encourage the authors to unpack this idea in more detail, please see my previous comments.

3) On alternative explanations for egocentric advice discounting
Finally, I still think the discussion would benefit from a more detailed consideration of alternative explanations for egocentric advice discounting. The authors provide a convincing argument that distrust may not fully explain the observed effects in their response to me. However, readers are likely to similarly consider distrust as one plausible account, and aspects of your response to my previous comment could therefore be incorporated into the manuscript to expand the discussion of alternative explanations further (alongside other explanations such as perfectionism/self-reliance motives or reputational concerns).

Overall, the manuscript has improved considerably, particularly in its treatment of metacognitive measures and learning dynamics. The remaining points mostly concern framing and clarification and should be straightforward to address.

** Visit Nature Research's author and referees' website at <a href="http://www.nature.com/authors">www.nature.com/authors</a> for information about policies, services and author benefits**

**Open Access** This Peer Review File is licensed under a Creative Commons Attribution 4.0 International License, which permits use, sharing, adaptation, distribution and reproduction in any medium or format, as long as you give appropriate credit to the original author(s) and the source, provide a link to the Creative Commons license, and indicate if changes were made.
In cases where reviewers are anonymous, credit should be given to 'Anonymous Referee' and the source.
The images or other third party material in this Peer Review File are included in the article's Creative Commons license, unless indicated otherwise in a credit line to the material. If material is not included in the article's Creative Commons license and your intended use is not permitted by statutory regulation or exceeds the permitted use, you will need to obtain permission directly from the copyright holder.
To view a copy of this license, visit https://creativecommons.org/licenses/by/4.0/

## Decision letter

Dear Dr Zonca,

Thank you for your patience during the peer-review process. Your manuscript titled "The advice gap: adaptive yet suboptimal advice integration" has now been seen by 4 reviewers, and I include their comments at the end of this message. They find your work of interest but raised some important points. We are interested in the possibility of publishing your study in Communications Psychology, but would like to consider your responses to these concerns and assess a revised manuscript before we make a final decision on publication.

We therefore invite you to revise and resubmit your manuscript, along with a point-by-point response to the reviewers. Please highlight all changes in the manuscript text file.

Editorially, we consider particularly important that you address the concerns raised about the baseline of decision revision without advice (R#1 & 3), about the recency effects regarding the integration of advice (R#2 and R#4) and the learning dimension (R#1-4), and that you clarify/discuss how you work integrate with the several relevant strand of literature identified by the reviewers (R#2, R#4).

Please also provide a sensitivity power analysis that is based on the smallest effect size of theoretical or pragmatic interest (SESOI) of all hypotheses. We require a scientific justification of what makes the specific target effect size the smallest effect size of interest. This should be informed by comparable effect sizes in the field of study (but not single studies).

As you revise the manuscript in response to these issues, please also implement all requests in the attached Mandatory Revision Requests document. All requirements listed in this document need to be fully met, or the work will be returned to you for further revisions without peer review. This workflow is in place to increase the likelihood that the paper will be accepted for publication. It reduces the number of rounds of revision (and review) and ensures that the reviewers vet a version of the article that is compliant with journal policies. If you have any questions regarding the required revisions, please contact the journal prior to resubmission to avoid a negative outcome.

We are committed to providing a fair and constructive peer-review process. Please don't hesitate to contact us if you wish to discuss the revision in more detail.

Please submit the following items:

- Revised manuscript
- Point-by-point response to the referees' comments
- Mandatory Revision Requests Table (attached).
- Cover letter (as a separate document)

via this link:
https://mts-commspsychol.nature.com/cgi-bin/main.plex?el=A5DU5sA4A2GaO7I2A9ftdQEwEWRnCEm2x9mTOa8IgAZ .

** This url links to your confidential home page and associated information about manuscripts you may have submitted or are reviewing for us. If you wish to forward this email to co-authors, please delete the link to your homepage first **

We hope to receive your revised paper within 8 weeks; please let us know if you aren't able to submit it within this time so that we can discuss how best to proceed. If we don't hear from you, and the revision process takes significantly longer, we may close your file. In this event, we will still be happy to reconsider your paper at a later date, provided it still presents a significant contribution to the literature at that stage.

We look forward to seeing the revised manuscript and thank you for the opportunity to review your work.

Best regards,

Troby Lui, on behalf of

Mael Lebreton

Troby Lui, PhD
Associate Editor
Communications Psychology

Mael Lebreton, PhD
Editorial Board Member
Communications Psychology
orcid.org/0000-0002-2071-4890

## Point-by-point response to the referees' comments

*REVIEWER REPORTS:*

***Reviewer #1 (Remarks to the Author):***

*The paper by Zonca et al. investigates how humans integrate advice in decision-making contexts, using a Bayesian model simulation as a benchmark to quantify how humans deviate from optimality. The authors conducted two experiments using a paradigm in which subject first made their own decision and then were given the advisor's decision and confidence, allowing them to calibrate their decision. There are seven blocks of trials, and in each block the advisor's attributes vary in one dimension: either accuracy, confidence, or predictability of confidence (confidence accuracy correlation). They also included a block where the advisor was comparable to the participant along all attributes. In Experiment 1 subjects were not informed of the advisors' attributes and in Experiment 2 they were informed. The authors found that while advice generally improves performance, the advice integration remains systematically suboptimal compared to the Bayesian optimality benchmark. This topic is interesting and the writing is generally clear, but I do have some theoretical concerns regarding the conclusion they draw as well as some questions for the results and methods.*

We thank reviewer #1 for the thorough and helpful review of our work. In the revised manuscript, we have conducted several additional analyses to address the reviewer's comments and concerns. In particular, we have 1) examined temporal and learning dynamics of advice use within blocks; 2) controlled for potential changes of mind; 3) used alternative measures of metacognitive abilities (meta-$I$ and meta-$I^r_2$, as also suggested by reviewer #4); 4) assessed participants' comprehension of the communicated information in Experiment 2; and 5) ran additional models to control for the effect of inclusion/exclusion of the initial phase of learning (Experiment 1). We have also clarified several methodological aspects highlighted by the reviewer, including the rationale behind our Bayesian model and technical details about the task implementation. We are convinced that these additions greatly improved ou manuscript. A detailed response to each comment is provided below.

*Major comments*

*1. For matched-accuracy advisers, the adviser isn't informationally superior, but subjects still showed an increase in their accuracy. While this is mathematically possible, as they just average two noisy sample from the same distribution and the accuracy might improve, I wonder whether the observed accuracy boost could be at least partly driven by the fact that they were just given a second chance to adjust their initial judgement (Resulaj et al., 2009). One potential way to look into this is, perhaps, by analyzing trials where agent's estimate closely matches the subject's initial estimate. If accuracy improves even when advisers provide minimal new information, this would suggest that revision opportunity effects exist.*

Given our manipulations of agents, 5 of 7 agents matched participants in accuracy. In all cases, final performance was superior to initial performance. As the reviewer correctly notes, this improvement could be expected even with a simple opinion-averaging strategy. However, we show that human participants adopted more sophisticated approaches: they took into account

not only their own and the agent's confidence, but also the agent's confidence average and predictivity, thereby modulating the weight assigned to the advice based on its expected trial-by-trial accuracy.

While we agree that small improvements could, in principle, be partly attributable to "changes of mind" (Resulaj et al., 2009), we believe this explanation is unlikely in the context of our first-order task. After the initial judgment, participants no longer have access to the perceptual stimulus and receive no additional information that could systematically shift their original opinion in the correct direction, apart from the advice itself. Also, it is important to note that our conclusions primarily rely on comparisons across agents or subjects. These comparisons implicitly control for any potential change-of-mind effect, assuming it remains constant across agents.

Nonetheless, as a robustness check, we conducted the re-analysis suggested by the reviewer. We examined trials where the agent's estimate closely matched the subject's initial opinion, using a 5° distance cut-off, consistent with our primary influence and accuracy analyses. In both experiments, when interacting with the five accuracy-matched agents, participants showed a significant increase in accuracy for trials with a distance ≥5° (Exp1: from 0.423 to 0.507; Exp2: from 0.439 to 0.544). Conversely, no accuracy gain was observed in trials with a distance < 5°(Exp1: from 0.652 to 0.646; Exp2: from 0.656 to 0.654).

*2. The Bayesian model functions as an ideal observer with perfect knowledge of task statistics, while human participants receive no explicit information about these statistics and face rapidly changing conditions (every 54 trials) in Experiment 1. Given that in studies that examine learning, human typically requires much more trials to learn (Behrens et al., 2007); here, participants were not given the opportunity to achieve something even close to the author's definition of "optimality". In this context, relying more on confident advisers represents a reasonable heuristic. Also, it might be helpful for the authors to analyze learning within blocks (first vs. second half) to assess adaptation, though 54 trials likely provide insufficient learning opportunity. Anyhow, the conclusions regarding "suboptimality" in Experiment 1 should be toned down.*

We thank the Reviewer for the comment on an important feature of our design, which gives us the opportunity to clarify this aspect more effectively. The Bayesian model is indeed an optimal model, as the Reviewer notes, but only in the sense that it performs optimal information updating. It does not possess more knowledge than the subjects, let alone "perfect knowledge" about the task statistics. A priori, before receiving the information from the 54 trials in each block (which is exactly the same information available to the subjects), the optimal model knows nothing about the agent's or subjects' characteristics beyond extremely general meta-hypotheses about the distributions of their accuracies and confidence predictivities. That is, at the beginning of each block, all the optimal model "knows" is that agents might be very good or very bad, and since there is no initial clue as to which is which, it adopts an average expected agent quality (uniform distribution over all the potential parameter levels). It is plausible that human subjects, who also begin the task knowing little or nothing, would start with similarly broad expectations about both themselves and the adviser agents: an average accuracy that could later prove to be very good or very poor once experience on the task accumulates. In Bayesian terms, we are then assuming that both the model and the subjects have "weakly informative" priors (Gelman et al., 2014), for the subject derived from general

experience, and for the model by training on a data set that included all possible agents' performances, and then averaging uniformly. This assumption is kept for all priors: accuracies, confidences, and confidence predictivities. This general prior knowledge is then necessarily discretized, for computational reasons, into ten "agent profiles", each for the three parameters: accuracy, confidence, and confidence predictivity. Yet. Yet, this forced discretization does imply that the model has perfect statistical knowledge of the task. On the contrary, if anything, the discretization into only 10 levels of performance might cause a (small) loss of information for the model compared to the subjects.

Beyond this very general prior about agents' characteristics, the model assumes that trials may vary in difficulty. But it has no information about which difficulty level will come next, nor about the distribution of difficulty levels across the block. It therefore adopts uniform priors once again (which, in this case, happened to match the true underlying distribution, although neither the model nor the subjects had any way of knowing this initially). Again, it is reasonable to assume that human subjects began the task with a comparable expectation: that some trials would be easier and some harder, but with total uncertainty about their relative frequencies.

As a consequence, we think that our optimal model does not have any knowledge advantage over the subjects. Actually, subjects and model share the same initial knowledge, and then both of them receive exactly the same stream of evidence during the block. As the Reviewer correctly points out, the model's only advantage lies in its "perfection": it combines its priors with incoming information in an optimally Bayesian way, and therefore it performs optimal Bayesian updating. Human subjects, as our data show, perform the same qualitative computations but with substantially less precision, leading to systematic suboptimality.

The main text was amended (Introduction) to better clarify that the optimal model did not have any knowledge advantage over the subjects.

Finally, we ran additional analyses to rule out the possibility that the human subjects simply lacked enough trials to learn to integrate advice to an optimal level and therefore reverted to relying on the "confidence heuristic". These analyses were included in the SI in a dedicated section of the Secondary/Exploratory endpoints ("Temporal and learning effects"). In brief, the results of Experiment 1 show that optimality in advice use increased significantly from the first third to the second third of trials, after which it remained stable. This confirms that while an early learning phase occurs, participants quickly reached a plateau in the second third of the block. This also validates our primary analytical approach, which excluded the first third of trials to ensure we were measuring stable integration behavior.

In contrast, no such early learning phase was observed in Experiment 2, where optimality was already high in the first third of trials. Together, these findings indicate that trial-by-trial learning in Experiment 1 was relatively rapid and did not continue to increase throughout the block. Conversely, the additional information provided in Experiment 2 allowed participants to effectively bypass this learning phase.

Given the results of these additional analyses, to answer the reviewer's comment about the emergence of a "confidence heuristic", we cannot exclude that participants used it to some degree to guide their behavior. Nonetheless. once they had learned the adviser's basic characteristics (e.g., the adviser's accuracy and confidence predictivity), a blind reliance on confidence became *unnecessary* and even *counterproductive*, especially when navigating agents with differing accuracy and confidence predictivity profiles.

References

Gelman, A., Carlin, J. B., Stern, H. S., Dunson D.B., Vehtari A., & Rubin, D. B. (2014). Bayesian data analysis. Chapman and Hall/CRC. p. 55

*3. In the introduction, the authors identified five sources of suboptimality, but they appear to overlap with each other conceptually. For example, "Integration errors" (combining information non-optimally) subsumes "failed adaptation" (not adjusting to adviser characteristics), as the latter represents a specific type of integration error. Similarly, "selective consideration" essentially describes suboptimal evidence weighting, another form of integration error. The authors might consider clarifying the distinctions.*

We agree with the reviewer that failed adaptation and selective consideration may also constitute additional examples of integration errors. To make both the conceptual relationships and the empirical distinctions more explicit, we revised the labels of each hypothesis, which now read as follows:

- Integration error – selection
- Integration error – combination
- Integration error – adaptation

We believe that these revised labels more accurately capture the commonalities among the hypotheses while also highlighting the specific characteristics of each class.

*Minor comments*
*- The correct response is defined as an "accuracy range" within 10 degrees, but how was this range determined?*

The accuracy range was determined through pilot studies to achieve an optimal balance between perceptual difficulty and the distribution of accuracy both within and between participants. This methodological parameter, along with the experimental task and design, was formally documented in our pre-registration. We also included this information in the revised manuscript (Methods, Individual decision task (IDT)).

*- For accessing metacognitive skills, why use correlation rather than established measures like meta-d' and M-Ratio?*

From a normative point of view, appropriately weighting a confidence signal requires a measure of how well that signal predicts accuracy. In the parlance of metacognitive research, this corresponds to metacognitive sensitivity, for which meta-d' is one possible measure. By contrast, M-ratio indexes metacognitive efficiency, which in our context is relevant only as an individual trait, useful, for instance, when exploring potential relations with optimality.

We are aware that correlation measures show some dependence on task performance (see Phi, Fig. 7 in Rahnev, 2025; however, note that task dependency is even stronger for meta-d'). This must therefore be taken into account when interpreting between-subject associations involving such indicators. On the other hand, correlation exhibits good test–retest reliability and is well-suited for continuous variables, whereas meta-d' requires preliminary discretisation, which entails some information loss.
Nevertheless, we share the interest (also raised by another reviewer) in exploring additional dimensions, such as metacognitive efficiency, as well as alternative measures of metacognitive

sensitivity to assess their potential relationship with optimality. The Reviewer #4 advised to use the meta-$I$ and meta-$I^r_2$ (Dayan, 2023). We have now conducted these additional analyses and reported them in the Results (section "Metacognitive skills predict optimality in advice integration") and in a dedicated section of the Supplementary Information ("Alternative measures of metacognitive ability").

Reference

Dayan, P. (2023). Metacognitive Information Theory. Open Mind, 7, 392–411.
Rahnev, D. (2025). A comprehensive assessment of current methods for measuring metacognition. Nature Communications, 16(1), 701.

*- The authors might consider reporting whether randomization/counterbalancing was used for adviser order, as order effects could confound results.*

The randomization strategy (random with constraints) is described in detail within the Experimental Procedure subsection of the Methods.

*- Can stimuli appear at any direction? If yes, how is the random guess rate calculated for continuous angular space? The "R" marker (indicating random guess reference line to subjects) explanation to participants should also be detailed.*

While stimuli may appear in any direction, the confidence scale reflects the probability of an accurate response, defined by the previously established ±10° accuracy range. Consequently, the "random guess" baseline is calculated as the probability of a response falling within this 20° window over the full 180° range of possible directions (if we consider one of the two halves of the plane). This results in an expected accuracy of approximately 11° (as rounded to the nearest integer) for a participant responding randomly.

*- The authors start introducing modeling results without clearly explaining what these models' variants are, which makes it really hard to follow.*

We integrated the main text with a brief description of these models, alongside the full specification in the Methods section ("Statistical analyses").

*- Are these typos in SI? Seems like it should be "Model 1.2" instead of "Model 1,2". Similarly, for "Model 2,1", "Model 2,2".*
*- Table numbering appears incorrect (S1-2 referenced after S3-4).*

Thanks for pointing out these mistakes! We amended them.

*- Why only excluding the first third of each block for model 1.3 to account for initial learning? Why is such exclusion not applied for model 1.1 and 1.2? This seems inconsistent. Also, this number (1/3) seems arbitrary.*

We excluded the first third of each block in Model 1.3 based on a conservative interpretation of the learning curves estimated during the piloting phase. These curves indicated that participants typically stabilized their use of advice features by this point. In the revised

Supplementary Information (section "Temporal and learning effects"), we show that this cutoff corresponds to a stabilization of optimality in advice use, with most gains occurring within approximately the first third of the block.
We nevertheless acknowledge that any cutoff involves a degree of arbitrariness. For this reason, we avoided post-hoc parameter tuning by preregistering the exclusion criterion in advance.

Regarding why this exclusion was not applied in Models 1.1 and 1.2, we note that these models were originally intended to capture overall accuracy outcomes in the presence of trial-by-trial learning, thereby characterizing how learning dynamics influence accuracy (e.g., in contrast to Experiment 2).
We nonetheless agree with the reviewer that it is informative to analyze accuracy outcomes while controlling for the initial learning phase, as this provides a cleaner comparison of initial, final, and optimal accuracy after relevant information has been acquired. Accordingly, we have added two supplementary models (SI, Primary endpoints, Models 1.1b and 1.2b; SI) in which the first third of trials within each block was excluded. The results are fully comparable across the two analytical approaches.

*- Figure 7 as an instruction seem too complex for subjects to interpret, such that understanding distributions and differential confidence for correct/incorrect trials requires some knowledge about stats/math. Were participants given additional instruction?*

An important contextual point is that our instructions were meant to convey only the relevant features of the agents (and of participants themselves), not to prescribe how these features should be used. What to do with the provided information had to rest on participants' intuitive understanding of the problem situation. We fully agree with the Reviewer that such information must be presented as clearly as possible.
Our participant pool consisted primarily of university students enrolled in bachelor's programs in economics and psychology. In both curricula, students routinely work with proportions, percentages, and basic distributional concepts. Presenting accuracy and confidence information as percentages was therefore consistent with their background knowledge and with the response format used during the task (participants reported their confidence as a 0–100% chance of being correct).
For the same reason, we opted to convey metacognitive sensitivity using mean confidence for correct and incorrect answers rather than a correlation-based metric. The simple difference between these two percentage values (or, graphically, the degree of overlap between the corresponding distributions) captures the essential information in a format that aligns with participants' own confidence reports, while avoiding a potentially more abstract statistical measure. Moreover, during the instructions, the presented graph was carefully described to participants in terms of its core components (x- and y-axes, bars, and colors). Participants were therefore explicitly instructed on how to read the graph, with illustrative examples. However, they were not instructed on how to use this information in the process of advice taking (e.g., relying more on confidence signals when the correlation is high). Besides, a basic definition and interpretation of the point-biserial correlation between confidence and accuracy (i.e., higher confidence for accurate judgments and lower confidence for inaccurate ones) was provided in the instructions when explaining the monetary incentives, as confidence-accuracy correlation was one of the parameters influencing performance and monetary reward. We have added this

information in the revised manuscript (Methods, Monetary incentives; Disclosure of participants' and agents' behavior in Experiment 2).
During the final debriefings in both pilot studies and the main experiments, no participants reported difficulties in understanding the instructions.

To further reassure the reader about participants' comprehension, we may refer to the new section (SI) dedicated to the temporal evolution of optimality across experiments ("Temporal and learning effects"). Results show that in Experiment 1, optimality increased from the first to the second third of trials and then stabilized in the other two sub-blocks, whereas no comparable early learning phase was observed in Experiment 2, where optimality was already high in the first third of trials. This finding indirectly suggests the full effectiveness of the provided agent descriptions in Exp 2.

Finally, we added analyses of participants' inter-block attribute estimates in Experiment 2 ("Inter-block performance ratings – Experiment 2"). These results indicate that the additional information provided in Experiment 2 effectively supported participants in identifying the relevant attributes of the agents.

*- The description of the polynomial mapping from mean accuracy to SD is not clear. In line 842-843 "The conversion followed a polynomial function with parameters [–358.5177 621.9958 –361.1622 81.3906]", these parameters seem to be the coefficients of a 3rd-order polynomial fit approximating the inverse of the function that maps SD to accuracy as there is no closed-form solution? However, this is not explained at all, nor did they explicitly state the analytic form of the underlying accuracy -> SD relationship they used.*

We thank the reviewer for pointing out that the description of the mapping between mean accuracy and SD was insufficiently clear. We agree and have clarified this procedure in the revised manuscript (Methods, "Distribution of the agent's perceptual judgments" section).
In our approach, the relationship between SD and expected accuracy is defined implicitly by the decision rule and the assumed sensory noise distribution, and it does not admit a closed-form inverse. Specifically, accuracy is determined by the probability that samples drawn from a truncated Gaussian angular error distribution (bounded between –90° and 90°) fall within the accuracy threshold (±10°), which cannot be analytically inverted to obtain SD as a function of accuracy.
To obtain this inverse mapping, we used a simulation-based procedure. For a range of SD values, we generated truncated Gaussian angular error distributions, extracted the corresponding expected accuracy, and averaged this relationship across repeated simulation runs. This yielded a numerical SD → accuracy function capturing the expected performance of an idealized observer under the task constraints. We then approximated the inverse of this empirical relationship (accuracy → SD) by fitting a third-order polynomial over the relevant accuracy range. The coefficients reported in the manuscript (i.e., [–358.5177, 621.9958, –361.1622, 81.3906]) correspond to this polynomial approximation.
This polynomial was subsequently used in the artificial agent to reproduce SD values for target accuracy levels, ensuring that the agent expresses the same normative relationship between variability and accuracy as the generative model, rather than an arbitrary or free-parameter mapping. In the revised manuscript, we explicitly state the polynomial's analytic form, clarify

that it approximates the inverse accuracy → SD relationship, and describe the simulation procedure used to derive it.

*- The authors used an iterative procedure to generate advisers' trial-by-trial behavior (page 33), and the tolerance threshold of this procedure progressively increased across iterations (up to 0.2). A tolerance of 0.2 on the scale of 0-1 is actually large, and thus the advisor behavior simulated might differ from intended. It might be clearer to report how often the algorithm had to rely on high tolerance values and the distribution of realized advisor attributes relative to target values at each participant x block level.*

We thank the reviewer for the suggestion. We have now included in the SI a specific section ("Agent generation - parameter accuracy") describing the accuracy of our agent generation approach.
Overall, discrepancies between target and actual agents' parameters were small. The mean absolute deviation (between-subject SD in parentheses) was 0.031 (0.009) for mean accuracy, 0.017 (0.005) for mean confidence, and 0.057 (0.023) for confidence predictivity. Agents with absolute deviations greater than 0.10 were rare (mean accuracy: 3% of agents; mean confidence: 0%; confidence predictivity: 16%), and became negligible when considering a tolerance of 0.20 (mean accuracy: 0%; mean confidence: 0%; confidence predictivity: 3%).
Importantly, our main analyses (e.g., Models 1.3, 1.4, 2.3, 2.4, ..) included the actual relative differences between each generated agent's parameters and the participant's corresponding parameters as predictors, thereby explicitly controlling for any residual deviation between target and implemented agent behavior.

*- In the current design, subjects provide confidence rating after their initial judgment and are not allowed to revise the confidence rating after seeing the adviser's estimate and confidence. This simplifies the modeling, but it would be interesting if subjects are allowed to change their confidence ratings to learn about metacognitive updating. They might change their confidence even when they keep or only slightly change their initial judgment.*

We agree with the reviewer that allowing participants to revise their confidence after receiving advice would provide valuable insight into metacognitive updating, including cases in which confidence changes occur independently of overt changes in judgment. Indeed, we have implemented such designs in separate experiments specifically aimed at addressing these questions.
In the present study, however, we deliberately chose not to include post-advice confidence ratings to simplify the task structure, reduce overall duration, and limit the number of modeled variables. We reasoned that the availability of a continuous, quantitative judgment signal was sufficient to capture advice integration behavior, without requiring an additional confidence report to disambiguate the relatively rare cases in which participants' final judgments coincided with either their initial estimate or the adviser's estimate.
We acknowledge that incorporating post-advice confidence measures represents an important direction for future work and are grateful to the reviewer for highlighting this point.

***Reviewer #2 (Remarks to the Author):***

*Comments on "The advice gap: adaptive yet suboptimal advice integration"*
*In this paper the authors examined the way participants integrate advice in a perceptual decision-making task. They manipulated the advisers accuracy, confidence and calibration, and examine how participants adapted their integration policy in the different conditions. This was done once with no explicit description of the advisers, and participants needed to learn advisers properties from experience, and once with full details available to participants. Finally, the authors generated optimal decisions based on a Bayesian integration model, which served as a benchmark for participants' optimal decisions.*
*The authors found that participants generally benefitted from advice, and were able to adapt their integration based on advisers' accuracy and calibration. However, participants over-relied on advisers' confidence. Overall participants advice integration was suboptimal, when compared with the optimal model. Explicit information did not shift advice taking behavior. Finally, individual metacognitive abilities were related to advice taking, as participants with more calibrated confidence were more optimal in their advice integration.*
*This is a carefully designed worked, with interesting findings. Some findings were already reported before, such as egocentric bias and reliance on confidence for advice integration. However, some other manipulations are novel, and the comparison of explicit and implicit description of advisers abilities is interesting. It was easy to read the paper, results are clear and methods are detailed. I appreciate the data availability, analysis code would be even better.*

We thank the reviewer for the thoughtful and constructive evaluation of our work. In the revised manuscript, and in line with the reviewer's suggestions, we have (1) more clearly situated our paper within the existing literature on advice taking, social influence, and learning; (2) conducted several additional analyses to examine temporal and learning dynamics, assess participants' comprehension of the communicated information, and evaluate effects related to the level of consensus between participants' and agents' responses; and (3) clarified methodological aspects highlighted by the reviewer. In addition, following the reviewer's suggestion, we have made the analysis code available in the online repository. We believe that these changes greatly improved ou manuscript. A detailed response to each comment is provided below.

*Comments*

*1. The framing of advice is not well defined in this paper. To the best of my understanding, advice was presented as virtual agent's advice, but no clear details about what participants thought was the source of advice, the intentions of agents, or their variabilities is described.*

We thank the reviewer for raising this point and agree that the advice's framing required clearer specification. In both experiments, advice was presented as originating from virtual agents characterized by stable performance properties rather than as social partners with explicit intentions. Participants were informed that they would interact with multiple advisers with varying skills, and that their task was to use available information to adapt their behavior to the specific adviser they were interacting with.
In Experiment 1, participants received trial-by-trial feedback on the correctness of their own response, allowing them to learn each adviser's properties through experience. In Experiment 2,

participants additionally received explicit information at the beginning of each block about the adviser's characteristics. In both cases, participants were provided with all the information required to compute an optimal response policy for each adviser.

Importantly, we deliberately avoided framing the advisers in terms of intentionality, communicative goals, or strategic behavior. Our aim was to evaluate participants' ability to use feedback and explicit performance-related knowledge to adapt their advice-taking behavior to a given source, and to assess whether and how suboptimality emerges despite sufficient information for optimal integration. Introducing assumptions about intentions or social motivations could have led participants to rely on heuristic strategies that were not central to the questions addressed here.

We have now clarified these aspects of the task framing and the information provided to participants in the Methods (section "Advised decision task (ADT)) and added a dedicated sentence at the beginning of the Results section).

*2. More broadly, this paper could be better situated within the literature on social learning. Advice is usually associated with agents intentionally sharing information in order to influence other's behavior (Hertz, Bell, and Raihani 2021), with some consequences for adviser (Anlló et al. 2024). It is different from observation or imitation, where there is no intentionality in sharing information (Najar et al. 2020). In addition, crowds can provide information (Yonah and Kessler 2021). These framings, especially advice, have profound effect on advice taking, usually leading to over compliance (Pereg et al. 2024) or to normative pressures (Mahmoodi et al. 2022, 2015). Can the author elaborate on how this work fits within these ideas?*

We thank the reviewer for this thoughtful suggestion and for highlighting important distinctions within the social learning literature, including advice, observation, imitation, and crowd-based information use. We agree that intentionality, normative pressures, and strategic information sharing play a critical role in many social learning contexts and profoundly shape advice-taking behavior.

Our study is best understood as complementary to this literature, rather than as directly addressing these social dimensions. Specifically, our primary goal was to isolate the cognitive mechanisms underlying information use, independently of social factors such as intentional communication, trust, reputation, normative influence, or strategic incentives. To this end, we deliberately designed the task to minimize social components in several ways.

First, advice was provided by artificial agents rather than human advisers, and agents were described as having stable performance characteristics without any reference to goals, intentions, or motivations. The agents' judgments consisted solely of a perceptual choice and an associated confidence estimate. No social framing, communicative intent, or strategic information sharing was implied or encouraged. In this sense, the "advice" in our task is closer to an externally provided informational signal than to socially embedded advice as typically studied in the social learning literature.

Second, participants were given full information about the agents' attributes, either through feedback or explicit descriptions, thereby reducing uncertainty about the agents' reliability and eliminating the need to infer intentions or hidden motives. This distinguishes our paradigm from studies emphasizing trust formation, reputational dynamics, or norm-driven compliance (e.g., Hertz et al., 2021; Anlló et al., 2024; Pereg et al., 2024).

Third, unlike work on imitation or observation learning (e.g., Najar et al., 2020), our task does not involve learning from others' actions via reinforcement or pseudo-reward mechanisms.

Similarly, unlike crowd-based decision-making paradigms (e.g., Yonah & Kessler, 2021), participants interacted with a single agent rather than aggregating information across multiple independent sources, thereby simulating typical assisted-decision-making scenarios (e.g., AI-assisted).
Within this controlled, non-social setting, our results show that participants nevertheless exhibit systematic, and partly suboptimal, patterns of information integration and use, from a purely cognitive perspective. Our findings provide a useful baseline for the broader social learning literature. By characterizing how confidence and accuracy cues are integrated in the absence of intentionality, normativity, or strategic interaction, our work helps disentangle which aspects of advice-taking behavior reflect fundamental cognitive constraints and which are specifically driven by social dynamics. We believe this distinction is particularly relevant for understanding advice use in emerging assisted decision-making environments, such as interactions with AI systems, where traditional social assumptions may not apply.
We have clarified this positioning in the revised manuscript (Introduction) to better situate our contribution relative to existing work on social learning.

*3. There seem to be a bias towards confidence as a signal of accuracy. This seems to be a relatively reliable heuristics, as in many real world scenarios this is the case. I think that this is the point of works from Bang, Bahrami and Flemming. I am not sure how your findings fit-in with their work.*

Our findings are compatible with this literature, while extending it by explicitly incorporating metacognitive skill, operationalized as confidence predictivity. In much of the previous work, confidence predictivity is either implicitly assumed or indirectly supported by a positive correlation between confidence and accuracy, which indeed makes reliance on confidence a generally beneficial heuristic.
Here, we show that participants adapt suboptimally to confidence predictivity: they rely too strongly on weak or non-predictive confidence signals and insufficiently on highly predictive ones. This suboptimal use of confidence predictivity has tangible behavioral consequences. In particular, when interacting with the high-predictivity agent (Pre+), participants exhibit a significant drop in accuracy relative to the optimal Bayesian model.
We agree with the reviewer that this limited (though not absent) adaptivity may, to some extent, reflect the use of a general "confidence heuristic" (e.g., Pulford et al., 2018) that is applied broadly across advisers and only partially depends on their actual confidence predictivity. Such a heuristic may be relatively common in real-world settings, where feedback and information about others' confidence predictivity can be sparse or unavailable in human–human interactions. However, many decision contexts provide rich information, either through decision feedback or explicit disclosure, and these contexts are highly relevant in practice. Consider, for example, a medical doctor relying on advice from an AI system whose attributes and performance are precisely specified by the developer/hospital (e.g., an AI that is highly accurate and exhibits strong metacognitive ability). In such cases, reliance on a generic confidence heuristic would be suboptimal, and effective decision making would require better adaptation to the adviser's specific characteristics. Our results suggest that this adaptation is not trivial: even under conditions of full information, decision makers tend to adjust only partially and suboptimally to the adviser's attributes, paving the way for targeted interventions.

References

Pulford, B. D., Colman, A. M., Buabang, E. K., & Krockow, E. M. (2018). The persuasive power of knowledge: Testing the confidence heuristic.Journal of Experimental Psychology: General, 147(10), 1431–1444. https://doi.org/10.1037/xge0000471

*4. It may be that participants show egocentric bias because advice appears after their own answer is presented – what if this was not the case, and they would see the advice before making their own decision? See (Hertz et al. 2016; Nafcha and Hertz 2022). It may be that anchoring on one's own advice is the root of bias in this work as well, and that tweaking the design may allow increased optimality.*

Our experimental design was not specifically tailored to quantify the relative contributions of egocentric bias, anchoring, or global-to-local deficits. We share the reviewer's interest in these mechanisms, and indeed, we are currently developing new studies aimed precisely at disentangling their respective impacts. For this reason, our present data cannot provide conclusive evidence on the question raised.
However, a recent study (Aiyer & Yeung, 2025) directly examined the role of the timing of advice. They found that post-stimulus advice, such as in our experiments, exerted a larger influence on participants' judgments than pre-stimulus, anchoring-like advice. Generalizing their findings to our context would imply that presenting advice before stimulus presentation would, if anything, lead to an even stronger egocentric discount, rather than reducing it.

*5. Do you think that the Bayesian model may have assumptions that are overoptimistic about human cognition, e.g., perfect estimation of sensory noise, perfect memory, unbiased integration of trial-by-trial confidence and accuracy, internal representation of noise and so on? There are currently works on resource-rational models of bounded computational cognitive capacity which may indicate some reasons for non-optimality here.*

Our Bayesian model was designed to compute a normative influence based solely on the evidence available to participants on each trial. It was therefore not intended as a psychologically realistic model of human cognition, but rather as an upper bound on potential improvements in advice integration and on the optimal weighting of the relevant dimensions. Comparing these optimal weights to participants' actual weights revealed two possible mechanisms underlying deviations from optimality: (i) a systematic overweighting of one's own opinion and confidence signals, and (ii) an inaccurate translation of global traits into local, trial-level influence. While the first mechanism seems more related to attentional or saliency biases than to computational limits, the second may reflect the accumulation of computational errors that effectively flatten the relationship between global variables and local decisions. Future work quantifying the relative contribution of these mechanisms would help clarify the cognitive mechanisms of suboptimality.
A clearer understanding of these cognitive determinants will also allow us to estimate whether, and to what extent, interventions could enhance participants' performance. In this regard, we see particular promise in our finding that participants considered and integrated all the dimensions prescribed by the Bayesian model, even if their integration was quantitatively suboptimal.

*6. Technical point – was there ever a negative influence? How do you measure this?*

Details about the calculation of the influence variable are reported in the Methods ("Main variables" section). To clarify in response to the reviewer's question, influence was intended to capture the proportional weight given to advice, ranging from 0 (no influence) to 1 (full influence). Because participants could place their final decision anywhere, even beyond the smallest angle between their initial and the adviser's response, we applied the following procedure.
If a participant's final decision fell outside the range α (i.e., the angular span between the two initial responses), that trial was normally discarded. To avoid unnecessarily excluding decisions that were very close to one of the initial judgments, we introduced a tolerance angle $\gamma=10°$. Within this tolerance, if the final decision was closer to the participant's initial estimate, it was treated as an influence of 0; if closer to the adviser's estimate, it was treated as an influence of 1. This threshold was chosen to be coherent with the accuracy range and to ensure that small deviations did not artificially remove trials that were effectively aligned with one of the two reference points.

*7. Accuracy effects – when an adviser is more accurate, it also means less distance from participant, compared with less accurate advisors. This leaves less space for improvement and change after viewing the advice. Did it affect influence?*

Influence is a relative index of advice weighting rather than an absolute angular deviation. By construction, it is normalized by the distance between the participant's initial response and the agent's response; therefore, smaller distances do not imply reduced room for adjustment, provided that a minimal discrepancy is present (a condition ensured by discarding trials with distances smaller than 5°).
Consistent with previous evidence (Zonca et al., 2023), we observed an overall negative relationship between distance and influence across adviser types, such that lower distance was associated with higher influence ($\beta = -0.01$, $p < 0.001$). However, when including the interaction with adviser type, this relationship was significantly modulated (omnibus interaction effect: $F = 15.57$, $p < 0.001$). Specifically, a negative distance–influence relationship was observed for agents Acc− ($\beta = -0.04$, $p < 0.001$), Conf− ($\beta = -0.02$, $p < 0.001$), and Pre+ ($\beta = -0.02$, $p < 0.001$).
In contrast, the only significant effect in the opposite direction was observed for the high-accuracy adviser (Acc+): when the adviser was more accurate, lower consensus (i.e., greater distance) was associated with increased reliance on advice ($\beta = 0.02$, $p < 0.001$). Together, these results indicate that response distance influences advice weighting not because of mechanical constraints on adjustment opportunities, but because distance itself acts as an informational signal whose interpretation depends on the relative accuracy of the participant and the adviser.
These additional analyses are now reported in the Supplementary Information (section "Effect of decision maker-adviser consensus on advice use").

References
Zonca, J., Folsø, A., & Sciutti, A. (2023). Social influence under uncertainty in interaction with peers, robots and computers. International Journal of Social Robotics, 15(2), 249-268

*8. In model 1.4 you report mean influence of 0.36 – why is this an egocentric bias? What would be the expected influence for no bias? I guess it should be 0.5, right? So is 0.36 really lower than 0.5? you compare it in the model to 0 in the model, right?*

We agree with the Reviewer that the intercept in Model 1.3 tests for a difference from zero rather than the theoretical optimal benchmark of 0.5; therefore, it does not constitute a formal statistical test for egocentric bias. In our original manuscript, the interpretation of egocentric bias in this context was based on a qualitative comparison with 0.5, with the formal test reserved for Model 1.4 (comparison against the Bayesian model's optimal benchmark). To address this and provide a more rigorous basis for our interpretation, we have added a complementary analysis to Model 1.3 (and Model 2.3 for Experiment 2). Specifically, we conducted a one-sample Wilcoxon signed-rank test to directly compare participants' mean influence against the 0.5 benchmark. In both experiments, the distribution of participants' mean influence is statistically different from 0.5 (one-sample Wilcoxon signed-rank test, Experiment 1: $z=-5.47$, $p < 0.001$, Experiment 2: $z=-5.58$, $p < 0.001$). These analyses have been added to the main text when presenting the results of Models 1.3 and 2.3 (Results).

*9. How was 'initial mean accuracy' defined? I could not find the definition.*

We thank the reviewer for pointing this out. In several instances, we had referred to "initial mean accuracy" when describing participants' mean judgment accuracy (i.e., the mean accuracy of participants' initial perceptual judgments in the ADT). We have now corrected the main text to consistently refer to this measure as *judgment accuracy*, as defined in the "Main variables" section of the Methods.

*10. Estimation of learning – did you fit a mode estimating participants trial-by-trial learning about the advisers' accuracy, confidence and calibration? Can participant learn about these? Experience with variance in advisors accuracy and confidence may lead to egocentric bias. While explicit information mitigates some of the learning issue, it may also be that participants find it hard to understand some features (confidence, calibration) while accuracy is easier to understand.*

We thank the reviewer for this important question. We did not fit an explicit trial-by-trial learning model estimating participants' beliefs about advisers' accuracy, confidence, or confidence–accuracy correlation, because these latent quantities were not directly elicited at the trial level. As a result, trial-wise belief updating about specific adviser attributes cannot be unambiguously reconstructed from behavioral data alone.
Instead, to assess whether and how participants learned about advisers over repeated interactions, we examined the temporal evolution of trial-by-trial optimality within each experimental block. This approach captures the behavioral consequences of learning or knowledge acquisition, regardless of which specific adviser attributes drive it. We have now included a dedicated section in the Supplementary Information ("Temporal and learning effects").
Results show that in Experiment 1, optimality increased from the first to the second third of trials and then stabilized during the final sub-block, indicating a relatively rapid learning process that did not continue to improve toward the end of the block. By contrast, in Experiment 2, no comparable early learning phase was observed: optimality was already high in the first third of trials. Together, these findings suggest that trial-by-trial learning was present and

relatively fast in Experiment 1, whereas the explicit information provided in Experiment 2 allowed participants to largely bypass this learning phase.

To further address concerns about participants' understanding and acquisition of adviser attributes, we added additional analyses in the revised Supplementary Information. Specifically, in the section "Inter-block performance ratings – Experiment 2", we show that participants discriminated agents along all relevant dimensions when explicit information was provided. This supports the conclusion that the disclosure manipulation in Experiment 2 substantially reduced uncertainty about adviser attributes and mitigated potential limitations in learning and comprehension.

Finally, we emphasize that particular care was taken during the instructions to clarify the more complex dimensions. In Experiment 2, the graph depicting confidence–accuracy point-biserial correlation during the attribute disclosure phase was explicitly explained in terms of its core components (x- and y-axes, bars, and colors), with illustrative examples to ensure correct interpretation. Importantly, participants were instructed on how to read this information but were not given prescriptive guidance on how to use it in advice taking (e.g., relying more on confidence when calibration is high). In addition, a basic definition and interpretation of this correlation (i.e., higher confidence for correct judgments and lower confidence for incorrect ones) was provided during the instructions, as this parameter directly influenced performance and monetary incentives. We have added this information in the revised manuscript (Methods, Monetary incentives; Disclosure of participants' and agents' behavior in Experiment 2).

*Anlló, Hernán, Gil Salamander, Nichola Raihani, Stefano Palminteri, and Uri Hertz. 2024. "Experience and Advice Consequences Shape Information Sharing Strategies." Communications Psychology 2 (1): 1–13.*

*Hertz, Uri, Vaughan Bell, and Nichola Raihani. 2021. "Trusting and Learning from Others: Immediate and Long-Term Effects of Learning from Observation and Advice." Proceedings. Biological Sciences / The Royal Society 288 (1961): 20211414.*

*Hertz, Uri, Margaux Romand-Monnier, Konstantina Kyriakopoulou, and Bahador Bahrami. 2016. "Social Influence Protects Collective Decision Making from Equality Bias." Journal of Experimental Psychology. Human Perception and Performance 42 (2): 164–72.*

*Mahmoodi, Ali, Dan Bang, Karsten Olsen, Yuanyuan Aimee Zhao, Zhenhao Shi, Kristina Broberg, Shervin Safavi, et al. 2015. "Equality Bias Impairs Collective Decision-Making across Cultures." Proceedings of the National Academy of Sciences 112 (12): 3835–40.*

*Mahmoodi, Ali, Hamed Nili, Dan Bang, Carsten Mehring, and Bahador Bahrami. 2022. "Distinct Neurocomputational Mechanisms Support Informational and Socially Normative Conformity." PLoS Biology 20 (3): e3001565.*

*Nafcha, Orit, and Uri Hertz. 2022. "The Devil Is in the Task Structure." ELife 11 (April): e78930.*

*Najar, Anis, Emmanuelle Bonnet, Bahador Bahrami, and Stefano Palminteri. 2020. "The Actions of Others Act as a Pseudo-Reward to Drive Imitation in the Context of Social Reinforcement Learning." Edited by Matthew F. S. Rushworth. PLoS Biology 18 (12): e3001028.*

*Pereg, Maayan, Uri Hertz, Ido Ben-Artzi, and Nitzan Shahar. 2024. "Disentangling the Contribution of Individual and Social Learning Processes in Human Advice-Taking Behavior." NPJ Science of Learning 9 (1): 4.*

*Yonah, Merav, and Yoav Kessler. 2021. "'they Don't Know Better than I Do': People Prefer Seeing for Themselves over Using the Wisdom of Crowds in Perceptual Decision Making." Journal of Cognition 4 (1): 30.*

***Reviewer #3 (Remarks to the Author):***

*I co-reviewed this manuscript with one of the reviewers who provided the listed reports. This is part of the Communications Psychology initiative to facilitate training in peer review and to provide appropriate recognition for Early Career Researchers who co-review manuscripts.*

We thank Reviewer #3 for the time devoted to reviewing our manuscript and for any comments provided.

***Reviewer #4 (Remarks to the Author):***

*I greatly enjoyed reading and reviewing this manuscript by Zonca and colleagues. This is really interesting and well-written work. I also appreciate that the study was pre-registered.*
*Overall, the manuscript tackles an important question: how people integrate external advice, how their own metacognitive ability influences this integration, and whether they take into account another advisor's accuracy and metacognition. The study design is thoughtful, and the attempt to benchmark human performance against an optimal model is commendable. However, substantial clarifications, deeper engagement with the existing literature, and some additional analyses are required before the manuscript is suitable for publication. I provide these comments in a constructive spirit: with revisions, the paper has the potential to make a really valuable contribution to the field.*

We thank Reviewer #4 for the detailed and insightful review of our work. Following the reviewer's suggestions, we have implemented several changes and additions to the manuscript.
First, we have better integrated our work within the literature on the measurement of metacognitive ability. In this context, we have more clearly situated our analyses within this methodological framework and conducted several additional analyses using meta-$I$ and meta-$I^{r}_{2}$ (Dayan, 2023) to assess the validity of our correlational measure and to control for effects related to first-order performance.
Second, the revised Supplementary Information includes several additional analyses examining (1) the temporal and learning dynamics of advice use within blocks; (2) participants' comprehension of the communicated information in Experiment 2; and (3) the accuracy of the artificial agent generation process.
Third, we have added analyses that were included in the preregistrations but were not reported in the original manuscript, as correctly noted by the reviewer.
Fourth, we have better situated our work within the broader literature on advice taking and metacognition.
Finally, we have clarified several methodological aspects highlighted by the reviewer, including the rationale behind our Bayesian model and technical details of the task implementation. We believe that these additions and revisions have substantially improved the manuscript. A detailed, point-by-point response to each comment is provided below

*Major Comments*

*1. Confidence Predictivity: definition, timing of introduction, and conceptual alignment*

*On page 3, the manuscript refers to "confidence predictivity" without explaining what this is until much later. This explanation should come earlier.*
*More importantly, confidence predictivity—operationalised as a correlation between error and confidence—is no longer the standard way the metacognition literature captures insight or metacognitive sensitivity. While I can see why this measure may have seemed practical in this task (particularly given the continuous, angular error measures), it is well established that such correlations are sensitive to first-order performance (e.g., Maniscalco & Lau, 2012; Fleming & Lau, 2014). This issue requires explicit discussion.*
*I appreciate that the advisors were already constructed based on this measure, and I am not suggesting the authors rewrite the entire experimental logic or run any additional experiments. However, additional post-hoc analyses using standard metacognitive metrics would greatly strengthen the manuscript. Meta-I(-ratio) seems most appropriate given varying difficulty; this can be easily calculated using the R toolbox statConfR (c.f. Dayan, 2023). These analyses would help demonstrate that the effects do not hinge solely on this potentially biased operationalisation.*

We thank the Reviewer for pointing out to us the rich methodological discussion on the appropriate measurement of metacognitive constructs. We are aware of and appreciate it. In the following, we detail the reasons we deemed the point biserial correlation ($r_{pb}$) an adequate choice for our specific needs.
From a normative point of view, appropriately weighting a confidence signal requires a measure of how well that signal predicts accuracy. In metacognitive research, this is labeled metacognitive sensitivity (and sometimes resolution), for which meta-d' (or meta-*I*) is a possible measure. By contrast, the M-ratio (or a normalized version of meta-*I*) indexes metacognitive efficiency, which in our context is relevant only as an individual trait, useful, for instance, when exploring potential relations with optimality.
We are aware that $r_{pb}$ may incorporate some dependence on task performance, even though this is admittedly also the case for other metacognitive sensitivity measures like meta-d' (see Fig. 7 in Rahnev, 2025; note that task dependency is even stronger for meta-d'). This dependency must therefore be taken into consideration when interpreting results involving such indicators, for example, when discussing the between-subject association between metacognitive sensitivity and initial accuracy. However, it is important to notice that this dependency has no impact on the interpretation of the main analyses of this study, i.e., on models based on optimality.
First, the manipulation of confidence predictivity (agents Pre+ and Pre–) is already controlled by design for task performance, as the two agents are generated with identical accuracy levels, which are also matched to the participants' accuracy. Consequently, any differences in behavior attributable to the agent manipulation cannot be explained by differences in first-order performance. Second, our main regression analyses include confidence predictivity (i.e., $\Delta$ confidence predictivity) as a predictor while explicitly controlling for task performance by including $\Delta$ accuracy as a covariate. As a result, the estimated effects of $\Delta$ confidence predictivity reflect variance in advice use that is independent of accuracy-related effects.
On the other hand, $r_{pb}$ is well-suited for continuous variables, while measures like meta-d' and Meta-I require preliminary discretisation, which entails some information loss (Rahnev, 2025). This is why we considered $r_{pb}$ as an appropriate choice in this context.

Nevertheless, we share the Reviewer's interest in exploring additional dimensions, such as metacognitive efficiency, in assessing alternative indices of metacognitive sensitivity (e.g., meta-I), and in better controlling for type 1 performance dependence.

We believe that concerns regarding dependence on task performance are most relevant for the analysis of inter-individual variability, where we observe a relationship between confidence predictivity and accuracy that could, in principle, arise from their shared correlation at the individual level. To address this issue, we added additional analyses in the revised Results section ("Metacognitive skills predict optimality in advice integration") that explicitly control for task performance.
First, we estimated an additional regression model predicting optimality that included accuracy and all corresponding interaction terms alongside confidence predictivity and treatment. Confidence predictivity remained a significant predictor of optimality after controlling for accuracy ($B = 0.51$, $p = 0.017$), while the interaction with experiment remained non-significant ($B = -0.26$, $p = 0.529$).
Second, following the reviewer's suggestion, we assessed metacognitive ability using meta-$I$ and meta-$I^r_2$(Dayan, 2023), which provide measures of metacognitive sensitivity and absolute metacognitive efficiency, which is intrinsically independent of first-order performance. These additional analyses revealed strong correlations between Meta-I, meta-$I^r_2$, and our confidence predictivity index, and further confirmed that both metacognitive sensitivity (meta-$I$) and efficiency (meta-$I^r_2$) significantly predict optimality in advice integration. Importantly, we also show that the agent manipulation based on confidence predictivity (Pre+ vs. Pre−) corresponds to systematic differences in both meta-$I$ and meta-$I^r_2$.
We added a dedicated section in the Supplementary Information ("Alternative measures of metacognitive ability") that provides details on the computation of meta-$I$ and meta-$I^r_2$.

Finally, we cited literature on metacognition and relevant measures in the Introduction.

*Furthermore, the term "confidence predictivity" is rare in the literature. It would be more consistent to refer to it as something similar to a phi coefficient to avoid proliferating new terminology or "toothbrushes"—keeping with the tongue-in-cheek metaphor by Elson et al. (2023).*

Phi, $r_{pb}$, or even meta-d' denote specific statistical indices used to quantify the same underlying construct, i.e., the relationship between confidence and accuracy. Different names have been used for this, e.g., "metacognitive sensitivity" or "resolution". We share the Reviewer's concern about introducing additional "toothbrush terms" and appreciate the incisive quote. At the same time, we believe that the label "confidence predictivity" more transparently conveys the measure's meaning, particularly to readers outside the metacognition subfield (e.g., those working in Human–AI collaboration, another target audience for this paper). We thought that the potential communication advantage would supersede the downsides of "label multiplication". To address the Reviewer's concern directly, we now explicitly state in the Introduction that confidence predictivity is equivalent to metacognitive sensitivity/resolution, and we relate it to the corresponding standard indices.

*2. Missing discussion of trust and suspected deception*
*The manuscript only briefly mentions trust, which is a central variable in advice-taking research (e.g., Pescetelli & Yeung, 2020).*

Pescetelli and Yeung (2021) examine trust as an evaluation of an advisor's accuracy and competence. They operationalize this construct by having participants rate the advisor on four dimensions: accuracy, confidence, influence, and trustworthiness.

Their findings are relevant and align with ours. Pescetelli and Yeung show that individuals can reliably estimate an advisor's trustworthiness after repeated interactions, even without feedback. Biases in trust judgments emerge only when advisors' errors are correlated. Furthermore, our model of optimal trial-by-trial integration considers for computing optimal weighting both accuracy and confidence, two critical components of the operationalization of trust in Pescetelli and Yeung. To these two, our model also adds confidence predictivity, critical if advisors differ under this parameter.

*People might distrust computer-generated or experimenter-generated advice, especially when advice is personalised or appears "too good." From my own anecdotal experience, participants often suspect deception throughout advice studies.*
*My concern is that the egocentric advice discounting observed here may partly reflect participants hedging against potentially malicious or unreliable advice, rather than a principled integration deficit. This should be explicitly discussed.*

The majority of participants were recruited from the Department of Economics subject pool, where deception is explicitly prohibited. This policy is clearly stated in the experiment-participant agreement. Consequently, participants typically expect full transparency regarding experimental procedures and incentive structures. No differences were observed in the subset of participants recruited from other sources.
Moreover, the task design itself leaves little room for perceived deception: truthful feedback was provided after every trial, enabling participants to verify the reliability of the advice. Even if some initial skepticism had been present, it would likely have dissipated once consistent feedback was observed. Also, skepticism here came at a monetary cost: if subjects had fully trusted and optimally integrated the advice, they would have increased their monetary outcome. Repeatedly observing the feedback and seeing that, by not trusting, the subjects were losing money, should have further discouraged the costly temptation to hedge against the possibility that the advice might be malicious.
Finally, our results show that participants' estimates of agent accuracy and. These results further suggest that participants were sensitive to differences in advisor quality and even tended to overestimate it. Additional results on participants' learning and performance ratings in both experiments (SI, sections "Temporal and learning effects" and "Inter-block performance ratings - Experiment 2") confirm that participants are sensitive to both trial-by-trial feedback and additional information provided in Experiment 2.
Overall, there is no indication that suspicion of deception contributed to the egocentric advice discounting observed in our study.

*Relatedly: Were participants debriefed? Did any report suspicion about the advisors? This information is essential for interpreting the findings.*

The task structure was faithfully explained to the participants; in Exp2, the instructions also included reliable estimates of the participants' and the agent's features. None of the participants reported feeling deceived.

*Moreover, the task environment is highly artificial. This is not a criticism per se—such paradigms are extremely useful precisely because they isolate integration mechanisms from real-world social*

*influences—but the limitations for generalising to naturalistic advice-taking require deeper discussion.*

We thank the Reviewer for this important observation regarding the task environment. We agree that our paradigm represents a deliberate simplification of complex, real-world social dynamics. As the Reviewer correctly notes, this simplification was a strategic choice: our primary goal was to isolate specific cognitive mechanisms of advice integration by minimizing the "noise" of confounding social factors. We believe that to fully understand the complexity of human-AI collaboration, it is first necessary to characterize these fundamental components in a controlled setting. By focusing on these core integration mechanisms, we provide a foundation for layering additional ecological social influences in future research.

Regarding the perceived "artificiality" of the task, we have revised the manuscript to better clarify the relationship between our lab setting and naturalistic environments. Specifically, while the paradigm is a simplification of human–human interaction, we argue that it maintains high ecological validity for many human–AI contexts (e.g., AI-assisted medical diagnosis; see Reverberi et al., 2022). In such professional settings, interaction is often restricted to the exchange of discrete advice (and related confidence), making our setup a faithful representation of that specific real-world niche.
We have expanded on these points and explicitly addressed the considerations for generalizing these findings to different decision contexts in both the Introduction and the Discussion.

*3. Benchmarking against an optimal model*
*A positive and valuable aspect of the manuscript is the explicit benchmarking of human performance against an optimal solution. This is useful for the field and the authors should keep this element prominent. However, this paper is by no means the first or only one to advocate such an approach, and additional relevant research should be cited and discussed. The authors already cite one such study (Bahrami, Olsen, Latham, Roepstorff, Rees, & Frith, 2010). I did a quick search and found Pescetelli, Hauperich, & Yeung (2021), letting me suspect there are likely other studies that have attempted this.*

We thank the Reviewer for this comment. We did not intend to claim to be the first to use optimal benchmarking or Bayesian models in advice-based decision-making; we agree that such a claim would be easily disproved given the existing literature. Nevertheless, we believe that the explicit consideration of an appropriate optimality benchmark remains relatively uncommon in the field, despite its importance for a rigorous interpretation of behavioral results.

We have revised the manuscript to more clearly situate our study within this "benchmarking" tradition, adding citations to several key studies, including Pescetelli et al. (2021) and Mahmoodi et al. (2015). We have also clarified how our implementation of an optimal Bayesian benchmark differs from previous work to address our research questions. In summary, our Bayesian approach differs from existing models in three key respects:

- Information Constraints: Many benchmarks assume "perfect knowledge" of agent statistics or "godlike" knowledge of probability distributions (e.g., Steyvers et al., 2022). In contrast, our model is conditioned strictly on the information available to the participant at each trial. This

makes it a true "normative" benchmark for human behavior rather than an idealized mathematical ceiling.

- Continuous vs. Dichotomic Estimation: Unlike the majority of previous work (e.g., Bahrami et al., 2010; Pescetelli & Yeung, 2021), which focuses on binary choices, our model operates on a continuous response variable. This allows us to assess trial-by-trial optimal influence with much finer granularity, capturing how much a participant should shift their judgment, rather than just whether they should switch categories.

- Dynamic Punctual Revision: While some studies use qualitative Bayesian expectations (Pescetelli & Yeung, 2021; Mahmoodi et al., 2015), our model calculates the optimal weight of advice dynamically on a trial-by-trial basis. This allows for a direct, quantitative comparison between actual human revision and the theoretical optimum.

Collectively, these features were critical for addressing our main research questions and, we believe, provide a valuable methodological framework for future research in the field.

*4. Learning over time*
*Was the task long enough for participants to actually learn or infer advisor quality? See, for instance, Carlebach & Yeung (2023) for work showing how confidence plays a key role in this gradual process.*
*One way to test this directly is to split the data into first versus second halves of the experiment and examine whether advice integration increases over time. This may also relate to trust-building (see above). This would of course be an additional, exploratory (and potentially slightly underpowered) analysis that would go beyond your preregistered analysis plan. I do believe, however, that it will be highly useful for readers to understand any such potential trends in your data.*
*This point is also linked to the phenomenon described as a global-to-local integration deficit, which is fascinating but appears a little underdeveloped. Page 22 would benefit from unpacking this idea in more detail. Have there been parallel findings in other research domains? How should a participant know the global characteristics of an advisor in Experiment 1 at the beginning of a block? Is this truly an integration deficit or simply learning in progress?*

We thank the Reviewer for this suggestion to explore the temporal dynamics of learning. We agree that distinguishing between "learning in progress" and "stable integration biases" is critical for interpreting our findings.
Following the Reviewer's suggestion, we performed an analysis of learning within blocks, now detailed in the Supplementary Information (SI, "Temporal and learning effects"). In brief:

- Experiment 1: We observed that optimality in advice use increased significantly from the first third to the second third of trials, after which it remained stable. This confirms that while an early learning phase occurs, participants quickly reached a plateau in the second third of the block. This also validates our primary analytical approach, which excluded the first third of trials to ensure we were measuring stable integration behavior.

- Experiment 2: No such early learning phase was observed; optimality was stable from the very first sub-block. This suggests that the additional information provided in Experiment 2 allowed participants to effectively "bypass" the inference process.

These results provide strong evidence that the observed suboptimality is not a result of "insufficient trials" or "learning in progress." Instead, it points to a persistent integration deficit: even when participants have effectively learned the advisor's characteristics (Exp 1) or were explicitly informed of them (Exp 2), they consistently failed to integrate that information optimally.

*5. Missing literature*
*As pointed out above, several important strands of literature are absent and should be integrated to ground the manuscript more firmly. In addition to papers mentioned above, one relevant missing study is Hertz, Palminteri, Brunetti, Olesen, Frith, & Bahrami (2017). Other relevant pockets of literature include work on information search and confidence (e.g., Steve Fleming's lab; Kobe Desender; Nick Yeung). This is intended as constructive criticism to further anchor the study in the existing literature.*

We thank the reviewer for the useful suggestions. We have now extended the cited literature both from the advice-taking-related fields (e.g., Hertz et al., 2017) and the metacognitive one (e.g., Dayan, 2023; Fleming & Lau, 2014; Fleming, 2023; Fitousi, 2025; Maniscalco & Lau, 2012, 2014; Rahnev, 2025).

*6. Preregistration inconsistencies*
*It is excellent that both studies were carefully preregistered. However, the section in the supplement where the authors list deviations from the preregistrations requires considerably more detail.*
*Relatedly, after briefly skimming the preregistrations, I noticed several preregistered tests missing from the manuscript (e.g., RT analyses, control analyses). On page 19 of the supplement, the authors state that "a few exploratory analyses were not reported in the final manuscript." But these analyses appear to have been preregistered and thus cannot be omitted.*

We thank the reviewer for pointing this out. In the SI (section "Secondary/exploratory endpoints), we have now added several additional analyses that were preregistrered but not included in the original manuscript. Many of these analyses were actually conducted to answer comments and concerns from the reviewers and were therefore coherently integrated in the main manuscript. These analyses include 1) temporal and learning effects; 2) effects of decision maker-adviser consensus on advice use; 3) inter-block performance ratings (Experiment 1 and 2); 4) accuracy of agent parameter generation; 5) interaction with an identical agent (AE); 6) Additional analyses on Reaction times.
The sentence "a few exploratory analyses were not reported in the final manuscript." has been removed from the section "Deviations from preregistered methods and analyses". This section was maintained to highlight the few/minor deviations from the preregistrations.

*7. Bayesian model fitting*
*I am not familiar with the GeNIe toolbox, so apologies if I am overlooking something. That said, I found myself wondering how we can be confident that the Bayesian model used to generate the optimal final decisions was indeed the most appropriate model for this task. At present, the manuscript does not report any formal model comparison, fitted parameters, parameter recovery, or model confusion analyses. These checks are typically essential for demonstrating that a model both fits the data well and is distinguishable from plausible alternatives. If these analyses were*

*conducted, it is necessary to include them; if not, I think it would be important to acknowledge this as a limitation.*

GeNIe Modeler is a graphical interface for the SMILE Engine, which provides both an inferential engine and a statistical library for Bayesian modelling. We used GeNIe and SMILE for practical reasons: they are freely available for academic research, widely used in published work employing Bayesian models, and they allow us to apply a GeNIe-built model iteratively to a long and complex sequence of inputs (i.e., one update per trial, with trial-by-trial updating within blocks and a reset to the agent's priors between blocks). However, the choice of GeNIe is not critical for the study. The same modelling approach could have been implemented by programming the Bayesian network from scratch; this would simply have required considerably more time.

The second part of the Reviewer's comment is especially helpful, as it highlights a potential lack of clarity in our use of the term optimal model. This expression has two distinct meanings. The first, which is the one we intend, is the normatively optimal or rational model, i.e., the model that specifies what an ideal reasoner *should* compute. The second meaning, which the Reviewer understandably inferred, is the model that best fits the empirical data among several candidates. These two meanings are related but not equivalent.

Following the principles of rational analysis articulated by Anderson (1991), one first specifies a normative model of the cognitive process of interest: what computations would a perfectly rational agent perform in an ideal environment? This model is logical, *not* empirical. One then compares this normative model to observed behaviour. If the two diverge, one progressively introduces constraints and assumptions (departures from perfect rationality) until the model closely matches the empirical data. At that point, one obtains an optimal model of the behavioural data, but this model is no longer normatively optimal. It is a constrained derivative of the normative model, designed to capture how real cognitive systems depart from ideal computation.

Our optimal model corresponds to the first meaning: it is the normative model of belief updating in a noisy signal-detection environment. Its structure follows standard information-theoretic principles: responses depend on signal plus noise; noise reflects both environmental uncertainty (dot coherence) and receiver noise (detector accuracy). Because neither stimulus difficulty nor detector accuracy is known, the model assigns maximal initial uncertainty to both. The same logic applies to confidence: in a perfectly calibrated receiver, confidence should be inversely proportional to noise, but if calibration is imperfect, confidence depends on both the noise and the receiver's estimate of that noise. The degree of calibration is also initially unknown, and again, the model assigns maximal uncertainty to all possible values. The only additional parameter is the non-informative "mean confidence level," which does not affect performance but is required for generating explicit confidence ratings.

The model, therefore, begins in a state of maximal uncertainty (uniform priors over accuracies, calibrations, stimulus difficulty, and confidence anchors). On each trial, it receives the available data (initial subject response, adviser response, final subject response, and ground-truth feedback) and updates all probability distributions using Bayes' rule, the normative updating

rule. Crucially, the model's purpose is not to fit participants' behavior; it is to provide a logical benchmark against which such behavior can be compared.

That said, the Reviewer's suggestion regarding model comparison and model selection points to a valuable direction for future work. One may indeed ask whether the normative Bayesian model, once endowed with different sets of priors or additional constraints, could become the model that best captures participants' behaviour—i.e., the second meaning of "optimal model." This is outside the scope of the present paper, but we are conducting a separate study that explicitly tackles it.

We have now amended the main text (Introduction) to clarify that the "optimal model" referred to in this study is the normatively optimal model, not a model optimized to fit the behavioural data.

*Minor Comments*
*1. The finding that participants weighted advisor confidence more heavily than advisor accuracy may have a simple explanation: advisor confidence is presented before feedback, whereas accuracy only becomes clear after feedback. In a complex task with many information streams, primacy effects are highly plausible. Please discuss this alternative interpretation.*

We consider two main types of confidence signals: mean confidence and trial-by-trial confidence.
The first is a trait of a participant or an agent. It tells us how accurate partners think they are, on average, across all trials. When compared with real average accuracy, it tells us whether partners are calibrated, over, or underconfident. The second is a trial-by-trial estimate of correctness.
Trial-by-trial confidence should generally have a strong impact on advice influence (even though modulated by mean confidence and confidence predictivity). The participants rightfully consider it, although not as much as they should, i.e., its impact is too weak, not too strong, when compared to an optimal benchmark.
Possibly the Reviewer is referring to the impact of mean confidence: in this case, we do report an excessive use of advice from the overconfident agent Conf+ (i.e., the agent whose mean confidence is largely above the real accuracy level) and overeliance on the mean confidence signal, when controlling for accuracy (Model 1.4). In fact, participants did rescale confidence based on the agent's mean value, but the adjustment was markedly weaker than optimal (Model 1.4b). However, we highlight that the observed reliance on the (mean) confidence signal is not stronger than reliance on the accuracy signal (similar beta estimates) in Experiment 1 (Model 1.3), and it is weaker in Experiment 2 (Model 2.3, $\Delta$ mean accuracy: B=0.3; $\Delta$ mean confidence: B=0.17). Given all that, primacy effects do not seem to have any explanatory role.

*2. Please clarify that Model 1.4 refers to a mixed-effects model and briefly outline what this model predicts and which predictors it includes. It took me until I read the supplement to understand that this was not a variant of the Bayesian ideal observer model.*

We thank the reviewer for pointing this out. We introduced Model 1.4 (and other mixed-effects models, when relevant) more clearly in the revised manuscript.

*3. I may have missed this, but I am still unsure why the Pre+ / Pre– agents were not defined relative to each participant.*

Agents Pre+ and Pre+ manipulate confidence predictivity, namely, the correlation between trial-by-trial confidence and accuracy. Because this metric is inherently less salient and harder to monitor than average accuracy or average confidence, we chose to maximise distinctiveness by creating more extreme agents: one with no predictivity at all and one with exceptionally high confidence predictivity. This feature is described and justified methodologically, in addition to the preregistrations, in the manuscript Methods section ("Designing agents' behavior").

*4. In Figure 1, the small number shown on screen (e.g., blue "49", red "76") is ambiguous. Is this the stimulus angle or the adviser's confidence rating? This could be more clearly explained in the caption and main text.*

The numbers always refer to confidence ratings, either from the adviser or from the decision maker. We have now stated this explicitly in the figure caption.

*5. When constructing personalised advisors based on participants' own accuracy and metacognition, how did the authors address cases where participants were at ceiling or bottom for accuracy/confidence/resolution? Some descriptive statistics on the distributions of variables such as sMA/sMC/sCR and aMA/aMC/aCR would be very helpful. Apologies if I missed this information somehow.*

During agent generation, we imposed minimum and maximum bounds of 0.1 and 0.9, and 10 and 90 for mean accuracy and mean confidence, respectively. This was meant to ensure a certain degree of variability even under extreme parameter values. In principle, when a participant has extreme parameter values, the adviser will have similarly extreme parameters, bounded at the minimum and maximum values. Agents' confidence predictivity was intrinsically fixed and thus independent of participants' confidence predictivity. Although we understand that, in principle, this may represent a problem for the agent manipulation (i.e., +-20% on the participants' parameters), for instance, when trying to create an agent with higher accuracy than a participant with extremely high accuracy, in fact, these hardly represented an issue, for several reasons.
First, extreme values were rare: no participants had a mean accuracy below 0.1 (participants with an accuracy below 0.15 were excluded, following the preregistered data-exclusion criteria), and no one exceeded 0.9 (max. accuracy: 0.73). No participants had a mean confidence below 10 (min. mean confidence: 26), and only one participant exceeded 90 (i.e., 94). Moreover, no participants had a confidence predictivity above 0.46, and predictivity levels close to 0 were rare (e.g., 4% of participants with $r < 0.05$).

We also added a section in the SI ("Agent generation - parameter accuracy") describing the accuracy of our agent generation approach.
Overall, discrepancies between target and actual agents' parameters were small, reassuring that ceiling effects had a marginal impact on the generation of artificial agents.

Furthermore, we emphasize that our main analyses (i.e., Models 1.3, 1.4, 2.3, 2.4, and relevant variants) included the *actual* relative differences between each generated agent's parameters

and the participant's corresponding parameters as predictors, thereby explicitly controlling for any residual deviation between target and implemented agent behavior. Figure S1 (SI) shows the actual distributions of relevant predictors for these statistical models (Δ accuracy, Δ mean confidence, Δ confidence predictivity), illustrating the distribution of our parameters of interest across participants and agents.

*6. Please include which software was used to present the stimuli. Was testing carried out in-person or online?*

The software used for stimulus presentation was Psychtoolbox for Windows version 3.0.16, a toolbox running under Matlab version R2020a. Testing was carried out in person. We added this information to the manuscript (Experimental Procedure).

*7. Figure 12 is intriguing, but one key point requires clarification: are the tertiles created across all conditions and kept constant? In other words, are the participants included in the green bar (ACT+) the same participants included in the green bar (ACT–)? Or are these averages computed across differing subsets? This affects how optimality should be interpreted.*

Tertiles were calculated based on ADT mean optimality, averaging across all experimental trials independently of agent type. Then we calculated the accuracy enhancement separately for the seven agent types. Thus, a specific tertile (e.g., green bars) always contains the same participants across all agent-type conditions. We clarified this methodological detail in the caption of Figure 12.
The figure is meant to represent the relationship between participants' *overall mean optimality* and the relative accuracy enhancement in interaction with different agents.

*8. The way accuracy and confidence were presented in Experiment 2 may not be ecologically valid. Prior work (e.g., Gigerenzer and colleagues) suggests that format strongly affects cognitive processes, in this case information integration. The authors should discuss whether the presentation format may explain the weaker advice integration in Experiment 2.*

An important contextual point is that our instructions were meant to convey only the relevant features of the agents (and of participants themselves), not to prescribe how these features should be used. What to do with the provided information had to rest on participants' intuitive understanding of the problem situation. We fully agree with the Reviewer that such information must be presented as clearly as possible.
Our participant pool consisted primarily of university students enrolled in bachelor's programs in economics and psychology. In both curricula, students routinely work with proportions, percentages, and basic distributional concepts. Presenting accuracy and confidence information as percentages was therefore consistent with their background knowledge and with the response format used during the task (participants reported their confidence as a 0–100% chance of being correct).
For the same reason, we opted to convey metacognitive sensitivity using mean confidence for correct and incorrect answers rather than a correlation-based metric. The simple difference between these two percentage values (or, graphically, the degree of overlap between the corresponding distributions) captures the essential information in a format that aligns with participants' own confidence reports, while avoiding a potentially more abstract statistical

measure. Moreover, during the instructions, the presented graph was carefully described to participants in terms of its core components (x- and y-axes, bars, and colors). Participants were therefore explicitly instructed on how to read the graph, with illustrative examples. However, they were not instructed on how to use this information in the process of advice taking (e.g., relying more on confidence signals when the correlation is high). Finally, we higlight that a basic definition and interpretation of the point-biserial correlation between confidence and accuracy (i.e., higher confidence for accurate judgments and lower confidence for inaccurate ones) was provided in the instructions when explaining the monetary incentives, as confidence-accuracy correlation was one of the parameters influencing performance and monetary reward.
On a more general note, an important body of research shows that individuals tend to exhibit more "Bayesian-like" behavior when learning from experience (sampling) than when using explicit probabilistic descriptions (Lejarraga & Hertwig, 2021). Any potential misunderstanding in the communicated agent features could have been rapidly fixed in the first trials, thanks to feedback. Moreover, we highlight that the summary feedback itself was conveyed in a format that was intentionally designed to be 'frequentist' in nature; for instance, confidence predictivity was illustrated through a distribution of actual past responses rather than abstract correlation coefficients.

We highlight that during the final debriefings in both pilot studies and the main experiments, no participants reported difficulties in understanding the instructions.

To further address concerns about participants' understanding and acquisition of adviser attributes (e.g., mean accuracy, mean confidence), we added additional analyses in the revised Supplementary Information. Specifically, in the section "Inter-block performance ratings – Experiment 2", we show that participants discriminated agents along all relevant dimensions when explicit information was provided. This supports the conclusion that the disclosure manipulation in Experiment 2 substantially reduced uncertainty about adviser attributes and mitigated potential limitations in learning and comprehension.

References
Lejarraga, T., & Hertwig, R. (2021). How experimental methods shaped views on human competence and rationality. Psychological Bulletin, 147(6), 535–564. https://doi.org/10.1037/bul0000324

*9. At the bottom of page 2, the phrasing can be misunderstood to mean that relative differences between advisors' cognitive/metacognitive skills were manipulated. In reality, this is the delta between participant and agent—please clarify.*

We thank the Reviewer for pointing this out, we clarified the above.mentioned sentence.

*10. On page 4 the minus sign is missing for the Pre– agent.*

Amended, thanks!

*Referenced Literature*
*Carlebach, N., & Yeung, N. (2023). Flexible use of confidence to guide advice requests. Cognition, 230, 105264. https://doi.org/10.1016/j.cognition.2022.105264*

*Dayan, P. (2023). Metacognitive Information Theory. Open Mind, 7, 392–411. https://doi.org/10.1162/opmi_a_00091*

*Elson, M., Hussey, I., Alsalti, T., & Arslan, R. C. (2023). Psychological measures aren't toothbrushes. Communications Psychology, 1(1). https://doi.org/10.1038/s44271-023-00026-9*

*Fleming, S. M., & Lau, H. C. (2014). How to measure metacognition. Frontiers in Human Neuroscience, 8(July), 443. https://doi.org/10.3389/fnhum.2014.00443*

*Hertz, U., Palminteri, S., Brunetti, S., Olesen, C., Frith, C. D., & Bahrami, B. (2017). Neural computations underpinning the strategic management of influence in advice giving. Nature Communications, 8(1). https://doi.org/10.1038/s41467-017-02314-5*

*Maniscalco, B., & Lau, H. (2012). A signal detection theoretic approach for estimating metacognitive sensitivity from confidence ratings. Consciousness and Cognition, 21(1), 422–430. https://doi.org/10.1016/j.concog.2011.09.021*

*Pescetelli, N., & Yeung, N. (2021). The role of decision confidence in advice-taking and trust formation. Journal of Experimental Psychology: General, 150(3), 507–526. https://doi.org/10.1037/xge0000960*

*Pescetelli, N., Hauperich, A. K., & Yeung, N. (2021). Confidence, advice seeking and changes of mind in decision making. Cognition, 215(October 2020). https://doi.org/10.1016/j.cognition.2021.104810*

## Point-by-point response to the referees' comments

REVIEWERS' COMMENTS:

*Reviewer #1 (Remarks to the Author):*

*I thank the authors for the thorough revisions. My concerns have all been addressed and the manuscript has improved substantially. I have only one remaining minor comment: in the rebuttal the authors explain that the ±10° accuracy window was chosen based on pilot studies, but I wasn't able to find that in the Methods. I suggest adding this detail to the Methods explicitly.*

We thank the reviewer for the positive evaluation of the revisions and are glad that the previous concerns have been addressed. Regarding the remaining comment, the manuscript specifies that the ±10° accuracy window was chosen based on pilot studies in the Methods section ("Individual decision task (IDT)" paragraph), where the accuracy range is introduced.

*Reviewer #2 (Remarks to the Author):*

*I appreciate the authors replies and additional analyses addressing my comments and questions. I have no further comments.*

We are pleased that the reviewer found our responses and the additional analyses helpful in addressing the comments and questions raised. We are grateful for the constructive feedback, which helped improve the manuscript.

*Reviewer #4 (Remarks to the Author):*

*The authors provided thorough and thoughtful revisions of their manuscript. Overall, I believe the manuscript has improved significantly.*

*In particular, I think the revisions regarding the measurement of metacognition have been handled well. The authors now define their central measure of 'confidence predictivity' early in the manuscript, clearly link it to established metacognitive measures, and provide additional analyses in the supplement using more standard indices. The manuscript is now much more firmly embedded in the metacognition literature (and I appreciate that it sits in-between different fields). This was a central concern in my previous review, and I think the authors have done a good job addressing it.*

We are pleased that the reviewers appreciated the amendments and additional analyses incorporated into the manuscript. As noted in our previous response, we found the feedback to be extremely helpful in improving the submission.

*I still feel a bit uneasy about the decision to introduce the term 'confidence predictivity', as this is not the standard label for this type of measure. My concern is primarily about potential label proliferation in a literature that already contains many overlapping constructs. That said, I recognise that the construct is now clearly defined and linked to established measures, and I do not*

*wish to insist further on this point, especially as it does not appear to have concerned the other reviewers to the same extent.*

Our previous manuscript served as a test case: we intended to keep the term "confidence predictivity" if our explanations of its rationale fully satisfied the reviewers. Otherwise, we planned to revert to the domain-specific "metacognitive sensitivity," clearly defining it for a broader audience in the introduction. Given your mention of "unease feelings," we have decided to adopt the latter approach!

*1) On claims regarding optimality*
*I still disagree with the sentence stating that "few studies have attempted to formally quantify (sub)optimality." As the authors themselves acknowledge, there is already a substantial body of work benchmarking behaviour against normative or optimal standards in this line of research. While I appreciate that additional relevant studies have now been cited, I would encourage the authors to further tone down this statement to avoid overstating the novelty of this aspect of their approach.*

We have addressed the reviewer's concern by removing the specified sentence. In the revised manuscript, we clarify the distinctions between our approach and previous studies to emphasise our novel contributions without diminishing the significance of foundational research.

*2) On the new learning analysis and the global-to-local integration deficit*
*I think the newly added learning analysis is a valuable addition to the manuscript.*
*However, I believe the second part of my previous comment regarding the global-to-local integration deficit has not been fully addressed. I would still encourage the authors to unpack this idea in more detail, please see my previous comments.*

We have addressed the reviewers' advice by providing a more thorough explanation of the "global-to-local integration deficit." Importantly, we have clarified that this concept can be understood as a specific instance of a much broader cognitive bias. The relevant section has been updated accordingly.

Nevertheless, egocentric discounting alone cannot explain all observed deviations. We identify a second source of suboptimality: a global-to-local integration deficit, defined as the difficulty of translating global knowledge of an adviser's competence into consistent, trial-level adjustments. While egocentric discounting establishes a default influence below the rational 50% benchmark with symmetric partners, the global-to-local deficit prevents decision-makers from fully acting upon the trial-by-trial implications of a partner's competence. This leads to a systematic under-modulation of advice weighting, a pattern that aligns with the classical anchoring-and-adjustment bias[55] or, more recently, with behavioral attenuation[56]. The latter is a widespread phenomenon where the responsiveness of decisions to relevant evidence is insufficient due to information-processing constraints. Thus, while egocentric discounting acts as an intercept bias, behavioral attenuation blunts the slope of the response curve to diagnostic traits, causing decisions to regress toward a simplified, middle-ground default.
In sum, suboptimality did not result from a lack of information, selective attention, or failed adaptation. Instead, it originated from two integration errors: a default overweighting of

self-generated judgments and a global-to-local deficit (a specific case of behavioral attenuation) in which diagnostic traits were weakly or inconsistently applied at the moment of decision.

*3) On alternative explanations for egocentric advice discounting*
*Finally, I still think the discussion would benefit from a more detailed consideration of alternative explanations for egocentric advice discounting. The authors provide a convincing argument that distrust may not fully explain the observed effects in their response to me. However, readers are likely to similarly consider distrust as one plausible account, and aspects of your response to my previous comment could therefore be incorporated into the manuscript to expand the discussion of alternative explanations further (alongside other explanations such as perfectionism/self-reliance motives or reputational concerns).*

We have addressed the reviewer's comment by expanding the Discussion to more explicitly consider distrust as a potential alternative explanation for egocentric advice discounting. In particular, we now note that participants received explicit trial-by-trial outcome feedback, which allowed them to evaluate and potentially revise initial assumptions about the adviser's reliability, thereby reducing the likelihood that persistent distrust accounted for the observed effects.

*Overall, the manuscript has improved considerably, particularly in its treatment of metacognitive measures and learning dynamics. The remaining points mostly concern framing and clarification and should be straightforward to address.*
