# Supplementary material for: Adaptive yet suboptimal integration of advice in decision-making
Source: Commun Psychol. 2026 May 16;4:123. doi: 10.1038/s44271-026-00456-1 (PMC13547347; doi:10.1038/s44271-026-00456-1)
Supplement: Supplementary file 2 [file 44271_2026_456_MOESM2_ESM.pdf]

Adaptive yet suboptimal integration of advice in decision making
- Supplementary Information -

Joshua Zonca [1,2], Alice Giampino [3], Paolo Cherubini [4], Carlo Reverberi [1,2]

Affiliations

1. Department of Psychology, Università degli Studi di Milano-Bicocca, Milan, Italy

2. Milan Center for Neuroscience, Università degli Studi di Milano-Bicocca, Milan, Italy

3. Department of Economics, Management and Statistics, Università degli Studi di Milano-Bicocca, Milan, Italy

4. Department of Brain and Behavioral Sciences, Università Statale di Pavia, Pavia, Italy

Correspondence

Joshua Zonca, joshua.zonca@unimib.it

Carlo Reverberi, carlo.reverberi@unimib.it

Table of contents

Supplementary Methods 3
Generating agents' behavior 3
Supplementary Results 5
Supplementary Figures 5
Full descriptive and statistical analyses - Primary endpoints 8
Experiment 1 8
Experiment 2 16
Secondary/exploratory endpoints 24
Alternative measures of metacognitive ability 24
Temporal and learning effects 25
Effect of decision maker-adviser consensus on advice use 26
Inter-block performance ratings - Experiment 2 27
Agent generation - parameter accuracy 28
Interaction with an identical agent 28
Additional analyses on Reaction times 28
Deviations from preregistered methods and analyses 29
References 29

Supplementary Methods

Generating agents' behavior

At the end of the IDT and the end of each block of the ADT, participants' perceptual judgments and confidence ratings were collected to compute three behavioral parameters:

1. **Participant Mean Accuracy (sMA):** This variable represents participants' average accuracy in their initial perceptual judgments, expressed as the proportion of accurate trials out of the total number of trials.
2. **Participant Mean Confidence (sMC):** The mean value of the distribution of participants' confidence ratings.
3. **Participant Metacognitive Sensitivity (sMS):** This metric entails two correlations: the Point-biserial correlation between trial-by-trial confidence and accuracy and the Pearson correlation between trial-by-trial confidence and absolute angular error.

Then, in each ADT block, these values were used as inputs to the algorithm that generated the virtual agent's behavior, randomly selected for the current block, as reported in Figure 3. After applying the parameter modulation based on the current agent type, the algorithm used the agents' parameters, such as aMA, aMC, and aMS, to generate perceptual judgments and confidence ratings.

Distribution of the agent's perceptual judgments

The aMA parameter, expressing the agent's expected mean accuracy, was used to derive the standard deviation (SD) of a Gaussian distribution of angular errors (i.e., deviations from the correct motion direction of the RDKs). Because the relationship between SD and expected accuracy is defined implicitly by the probability that angular errors fall within the accuracy tolerance (±10°) and involves a truncated Gaussian distribution (bounded between –90° and 90°), this mapping does not admit a closed-form inverse.

To obtain the inverse mapping from mean accuracy to SD, we used a simulation-based procedure. For a range of SD values, we generated truncated Gaussian angular error distributions, computed the corresponding expected accuracy as the proportion of samples within the accuracy tolerance, and averaged this relationship across repeated simulation runs. This yielded a numerical SD → accuracy function reflecting the normative relationship between perceptual variability and performance under the task constraints.

We then approximated the inverse of this empirical relationship (accuracy → SD) by fitting a third-order polynomial over the relevant range of accuracy values. The resulting polynomial coefficients were [–358.52, 622.00, –361.16, 81.39] and were used to compute SD values given target mean accuracy levels in the agent. We constrained mean accuracy to lie between 0.1 and 0.9 to allow variability in perceptual judgment responses while avoiding degenerate values at ceiling or floor performance.

Once the SD value was estimated for a specific agent, it was used as the standard deviation of a Gaussian distribution (centered on 0 and truncated at [–90, 90]) of the agent's angular errors. The Gaussian distribution was combined with a uniform distribution over [–90, 90]. The combination of these two distributions resembled the most typical distribution of angular errors among human participants, as estimated in a pilot study. Indeed, when extremely uncertain, human participants tended to produce uniformly distributed errors (due to random responses). These random errors occurred more often for participants with higher standard deviations in their angular error distributions. Therefore, we manipulated the relative proportions of observations from the Gaussian and uniform distributions based on the standard deviation of the participant's error distribution. Starting from a total number of 60,000 observations, the number of observations composing the uniform distribution was ⅙ for SD < 16, ⅓ for SD in the range [16, 34], and ½ for SD > 34. Finally, the 60,000 angular error values were converted to their corresponding absolute values for subsequent steps.

Distribution of the agent's confidence ratings

The aMC was used to generate a distribution of the agent's confidence judgments, using mean = aMC. We set a minimum mean confidence of 10 and a maximum mean confidence of 90. These bounds were intended to allow sufficient variability in confidence responses, even when average confidence is extreme. The standard deviation of the confidence distribution was set to be mathematically dependent on the mean confidence. This was necessary to generate a distribution with Gaussian properties, even for extreme aMC values. For this reason, we associated relatively low and high aMC values (close to 10 or 90) with a lower standard deviation. At the same time, SD was higher for an aMC of 50 (the mean value of the potential confidence ranges). The SD was set to 6, yielding a mean confidence value of 10. Then, it increased by 3 units for an increase of 5 units of mean confidence, reaching a maximum of 30 at aMC = 50. Then, it decreased again by 3 units, increasing the mean confidence by 5 units, reaching a minimum of 6 at aMC = 90. Similarly, the skewness of the distribution was based on the aMC value (larger absolute skewness for extreme aMC values, range [–1.1, 1.1]). The same applied to the distribution kurtosis (larger kurtosis (3.2) for extreme aMC values, and kurtosis decreasing with lowest kurtosis (2.4) for aMC = 50). Also, in this case, the Gaussian distribution contained 60,000 values and was truncated to values [0,100]. This truncation followed the actual confidence scale from 0% to 100%.

Manipulation of the agent's metacognitive sensitivity

The two vectors of absolute angular errors and confidence ratings were manipulated to match the predicted agent's metacognitive sensitivity (aMS). Therefore, the order of the values in the two distributions was reshuffled to match the desired (point-biserial) correlation, yielding two ordered vectors of absolute angular error and confidence ratings, each showing a specific negative correlation. Moreover, absolute angular errors below 10° were considered accurate (1), and higher errors were considered inaccurate (0), resulting in a third vector of 0s and 1s indicating the agents' accuracy.

Selection of trial-by-trial agent's behavior

In each block (within the same virtual agent), we generated perceptual and confidence judgments. This trial-by-trial selection had to lead to perfect alignment with the final agent behavior we aimed to achieve. However, a random selection of 54 perceptual judgments and confidence values corresponding to the 54 trials of the experimental block would likely have yielded statistical properties poorly aligned with expectations.

Considering the two ordered vectors of 60,000 values of absolute angular error, accuracy, and confidence, a set of 54 pairs was selected by iterative shuffling. In each iteration, the algorithm computed the mean accuracy, mean confidence, Pearson's correlation between confidence and absolute angular error, and the point-biserial correlation between confidence and accuracy, and compared them with the expected values aMA, aMC, aMS for the current agent. In the first 15,000 iterations, the algorithm stopped shuffling if all parameters were within 0.025 of the expected parameters (for accuracy, correlation between confidence and accuracy, and correlation between confidence and absolute angular error) or within 2.5 of the expected parameters (for mean confidence and SD of angular error). After 30,000 iterations, this tolerance was increased to 0.05 (5). After 20,000 iterations, the tolerance was further increased to 0.10 (10). Finally, after 45,000 iterations, the tolerance value was set at 0.20 (20). This procedure aimed to construct agents as close as possible to the theoretical behavior specified by the experimental manipulation.

Once the 54 pairs were selected, each of the 54 error values was randomly assigned a positive or negative sign (+ or –). This returned 54 trial perceptual errors and 54 confidence judgments with an underlying correlation (aMS).

In the Supplementary Information ("Agent generation - parameter accuracy" section), we illustrate the final accuracy of our agent generation approach.

Correlation with dots' coherence

The two ordered vectors of perceptual errors and confidence judgments were associated with a vector containing the three different coherence values (16 items per coherence level) in a randomized order. The

coherence vector was shuffled until a Pearson's correlation of r = 0.15 (± 0.03) with the perceptual error vectors was found. In this way, the agent's perceptual errors followed a typical human pattern, underpinned by a correlation between sensory uncertainty and judgment accuracy. The value of r = 0.15 matched the average correlation of a human pool collected in a pilot study.

Supplementary Results

Supplementary Figures

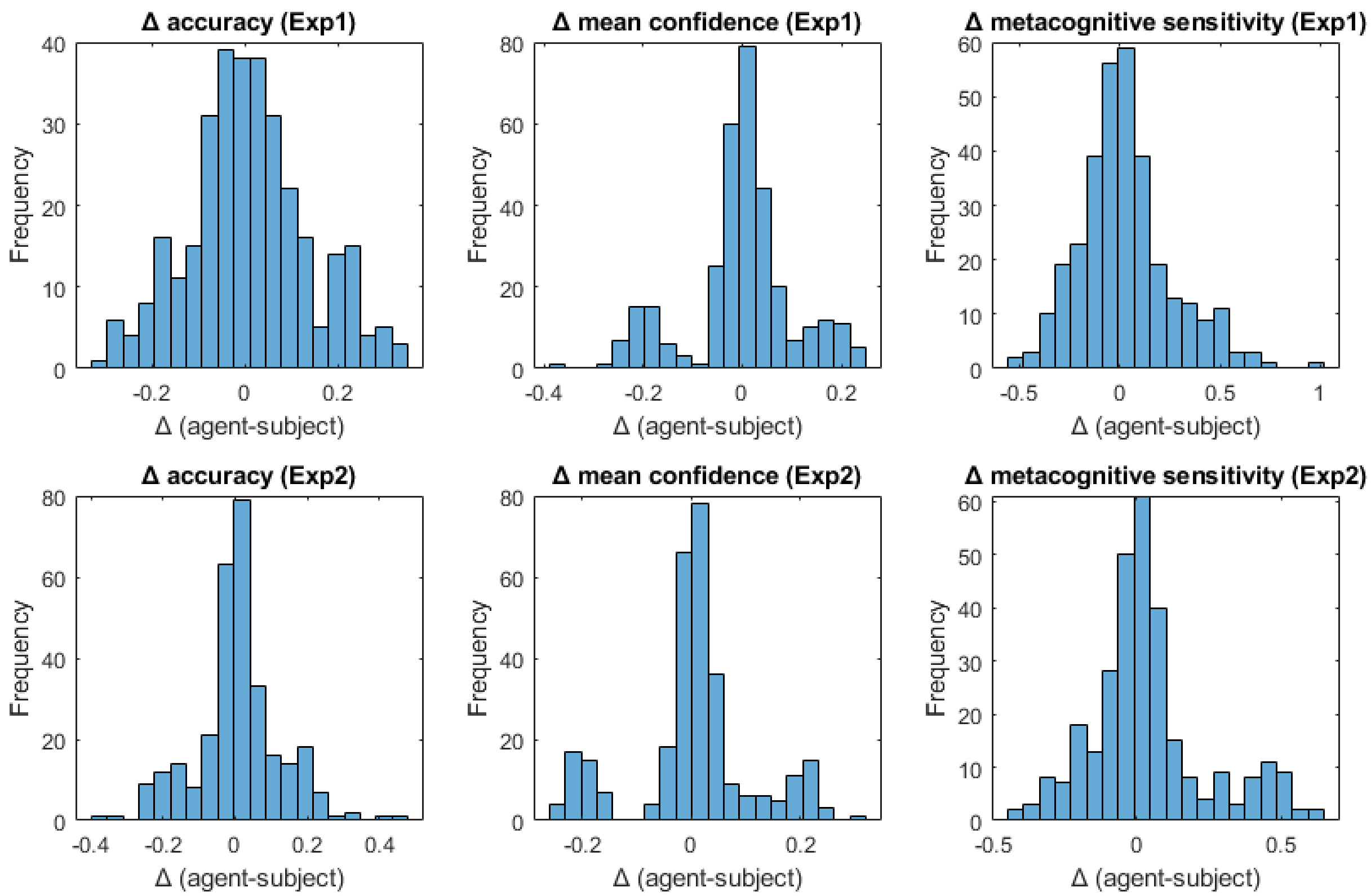

Figure S1. Distribution of relevant predictors for statistical models (Δ accuracy, Δ mean confidence, Δ metacognitive sensitivity). Distribution of relevant variables expressing the relative difference between the agent's and the subject's attributes, considering all seven blocks of the experiments. The upper panels refer to Experiment 1, the lower panels to Experiment 2.

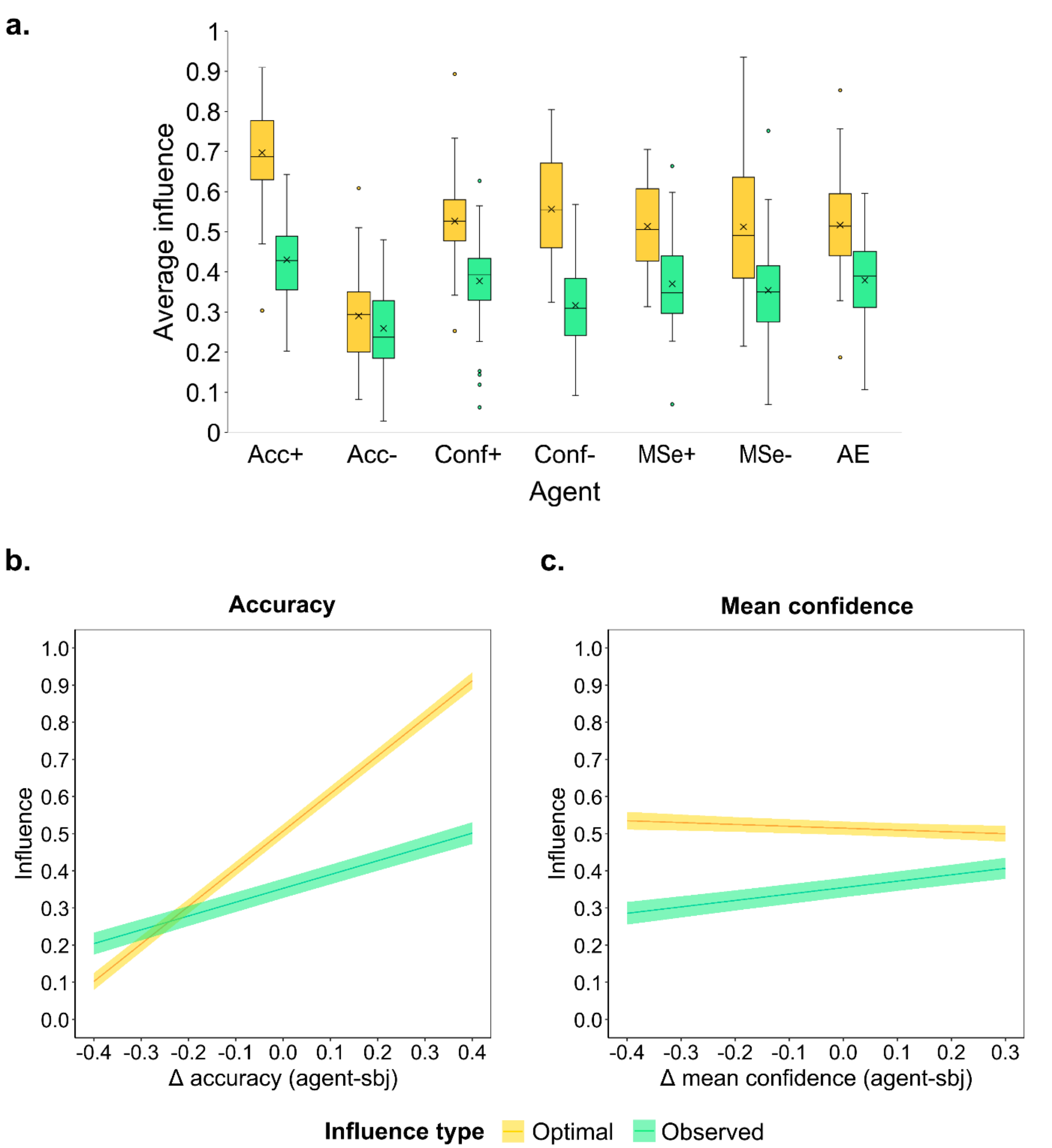

Figure S2. **Modulation of advice use (Exp 2). a)** Boxplots of participants' mean influence (observed in green, optimal in gold) across the seven adviser conditions. The box encompasses the interquartile range (IQR) with the internal horizontal line representing the median and the 'X' marker indicating the arithmetic mean. Whiskers extend to the maximum and minimum values within 1.5*IQR, while individual points denote statistical outliers. **b)** Model 2.4 estimates (unstandardized betas) of participants' influence as a function of *Δ accuracy* (agent - subject) by influence type (observed in green, optimal in gold). Ribbons express 95% confidence intervals of the regression estimates. **c)** Model 2.4 estimates (unstandardized betas) of participants' influence as a function of *Δ mean confidence* (agent - subject) by influence type (observed in green, optimal in gold). Ribbons express 95% confidence intervals of the regression estimates.

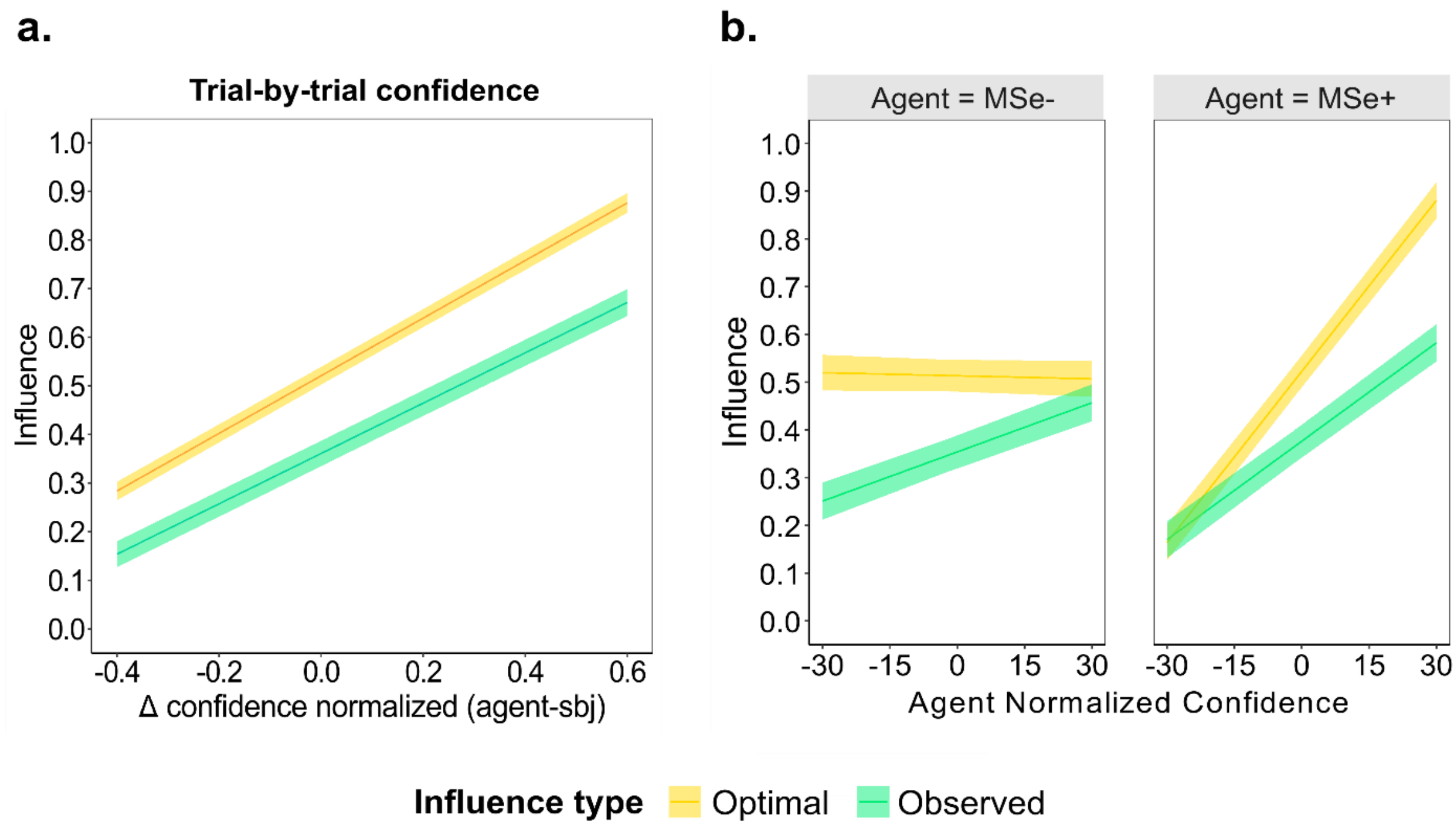

Figure S3. **Use of trial-by-trial confidence (Exp 2). a)** Model 2.4 estimates (unstandardized betas) of participants' influence as a function of Δ normalized trial-by-trial confidence (agent - subject) by influence type (observed in green, optimal in gold). Ribbons express 95% confidence intervals of the regression estimates. **b)** Model 2.4 estimates (unstandardized betas) of participants' influence as a function of the agent's normalized trial-by-trial confidence by influence type (observed in green, optimal in gold). The left panel refers to interactions with the agent MSe-, the right panel to interactions with the agent MSe+. Ribbons express 95% confidence intervals of the regression estimates.

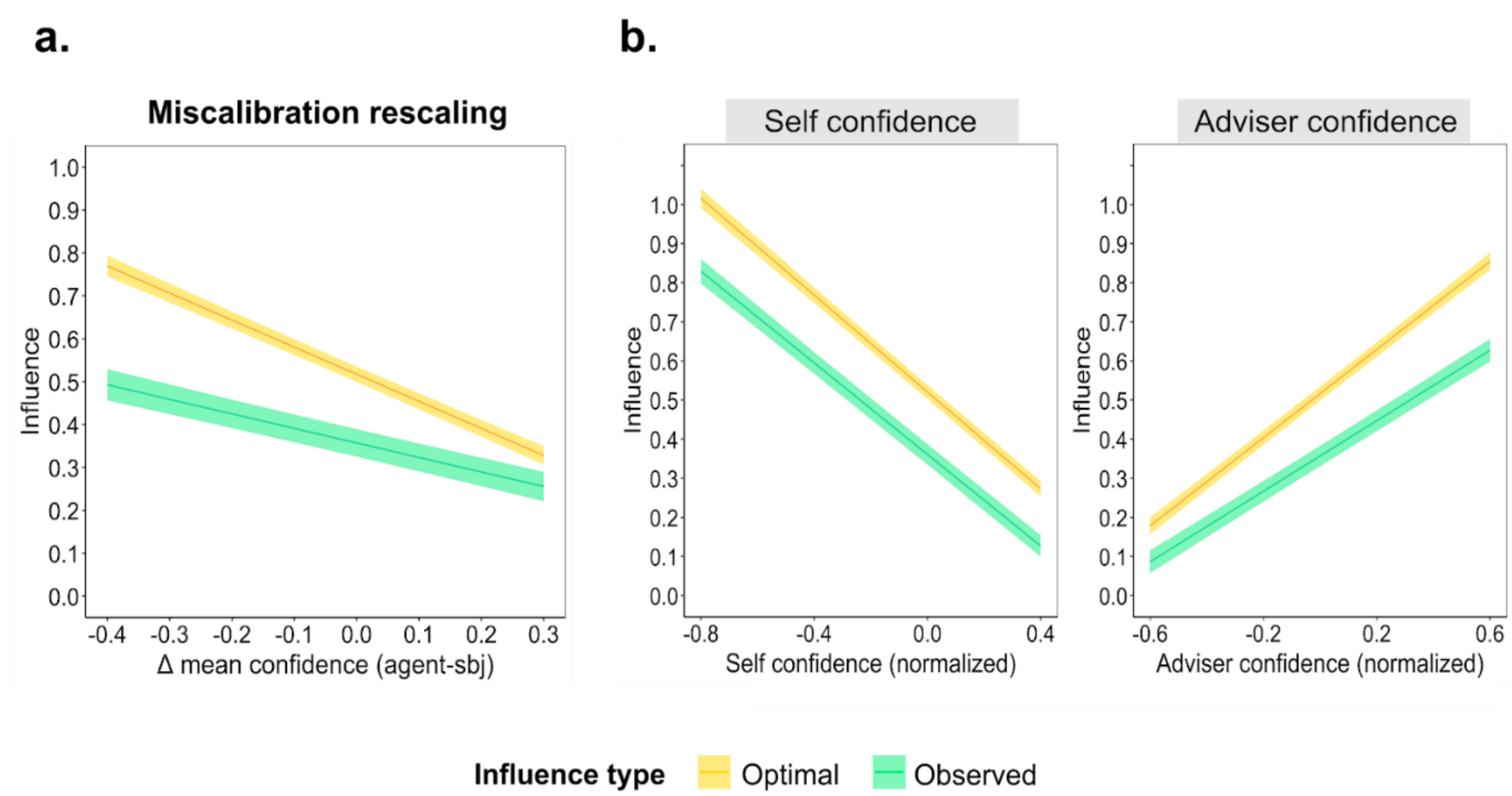

Figure S4. **Use of trial-by-trial confidence (Exp 2). a)** Model 2.4b estimates (unstandardized betas) of participants' influence as a function of *Δ mean confidence* (agent - subject) by influence type (observed in green, optimal in gold). Ribbons express 95% confidence intervals of the regression estimates. **b)** Model 2.4c estimates (unstandardized betas) of participants' influence as a function of the agent's normalized trial-by-trial confidence by influence type (observed in green, optimal in gold). The left panel refers to the *self confidence* regressor, the right panel to the *adviser confidence* regressor. Ribbons express 95% confidence intervals of the regression estimates.

Full descriptive and statistical analyses - Primary endpoints

Experiment 1

Adviser	Initial accuracy (subjects)	Final accuracy (subjects)	Optimal accuracy (Bayesian model)
**Acc+**	0.37 (0.16)	0.54 (0.17)	0.65 (0.15)
**Acc-**	0.45 (0.17)	0.46 (0.21)	0.47 (0.21)
**Conf+**	0.43 (0.15)	0.50 (0.20)	0.53 (0.18)
**Conf-**	0.43 (0.15)	0.50 (0.17)	0.54 (0.19)
**MSe+**	0.42 (0.15)	0.53 (0.17)	0.61 (0.17)
**MSe-**	0.43 (0.15)	0.51 (0.19)	0.51 (0.17)
**AE**	0.43 (0.16)	0.50 (0.19)	0.54 (0.20)
**All**	0.42 (0.14)	0.51 (0.17)	0.55 (0.17)

Supplementary Table S1. Descriptive statistics reporting the average accuracy of participants' initial judgment, final decision, and accuracy of Bayesian model optimal decision, by adviser type. Between-subject standard deviations in brackets. These descriptive statistics exclude trials in which the distance between the participant's and the agent's responses was less than 5°, consistent with the exclusion criteria used for computing influence and optimal responses. In this table, however, we did not exclude the first third of trials in each block to illustrate overall accuracy outcomes in the presence of trial-by-trial learning. Anyway, the results of models with and without excluding the first third of trials are fully comparable (see Model 1.1 and Model 1.1b below).

Adviser	Observed influence (subjects)	Optimal influence (Bayesian model)
**Acc+**	0.42 (0.14)	0.70 (0.13)
**Acc-**	0.30 (0.12)	0.35 (0.12)
**Conf+**	0.40 (0.13)	0.60 (0.13)
**Conf-**	0.30 (0.11)	0.55 (0.14)
**MSe+**	0.39 (0.11)	0.51 (0.13)
**MSe-**	0.36 (0.12)	0.57 (0.16)
**AE**	0.37 (0.13)	0.54 (015)
**All**	0.36 (0.10)	0.55 (0.11)

Supplementary Table S2. Descriptive statistics reporting the average influence of participants (observed influence) and average influence suggested by the Bayesian model optimal decision (optimal influence), by adviser type. Between-subject standard deviations in brackets. The first third of trials in each block was excluded from the analysis.

Dv: Accuracy	df	X^2	p
Decision type	2	352	< 0.001
Adviser type	6	224	< 0.001
Decision type *Adviser type	12	485	< 0.001
N. obs	44019		
N. groups	46		

Supplementary Table S3. Omnibus results of Model 1.1 (all trials).

Dv: Accuracy	B	Std. Err.	z	p	95% Conf. Inter.	
Acc+						
Subject - Final	-0.7182	0.0665	-10.798	<.001	-0.849	-0.5879
Agent - Final	0.2993	0.0658	4.551	<.001	0.170	0.4282
Acc-						
Subject - Final	-0.0461	0.0632	-0.728	.466	-0.170	0.0779
Agent - Final	-1.0985	0.0689	-15.943	<.001	-1.234	-0.9635
Conf+						
Subject - Final	-0.3442	0.0651	-5.290	<.001	-0.472	-0.2167
Agent - Final	-0.4309	0.0653	-6.594	<.001	-0.559	-0.3028
Conf-						
Subject - Final	-0.3102	0.0646	-4.802	<.001	-0.437	-0.1836
Agent - Final	-0.4189	0.0649	-6.458	<.001	-0.546	-0.2918
MSe+						
Subject - Final	-0.4879	0.0646	-7.548	<.001	-0.615	-0.3612
Agent - Final	-0.5178	0.0648	-7.997	<.001	-0.645	-0.3909
MSe-						
Subject - Final	-0.3404	0.0649	-5.243	<.001	-0.468	-0.2131
Agent - Final	-0.4090	0.0651	-6.284	<.001	-0.537	-0.2815
AE						
Subject - Final	-0.3280	0.0649	-5.054	<.001	-0.455	-0.2008
Agent - Final	-0.4141	0.0652	-6.357	<.001	-0.542	0.2864
N. obs	44019					
N. groups	46					

Supplementary Table S4. Simple effects of Model 1.1 (all trials).

Dv: Accuracy	df	X²	p
Decision type	2	264	< 0.001
Adviser type	6	175	< 0.001
Decision type *Adviser type	12	306	< 0.001
N. obs	29346		
N. groups	46		

Supplementary Table S3b. Omnibus results of Model 1.1b (first third of block trials discarded). This is a version of model 1.1 in which we discard the first third of trials in each block, to assess the accuracy effect of the final decision, controlling for the initial learning phase.

Dv: Accuracy	B	Std. Err.	z	p	95% Conf. Inter.	
Acc+						
Subject - Final	-0.7532	0.0811	-9.284	<.001	-0.9122	-0.594
Agent - Final	0.2062	0.0801	2.573	0.010	0.0491	0.363
Acc-						
Subject - Final	-0.0300	0.0774	-0.388	0.698	-0.1818	0.122
Agent - Final	-1.0788	0.0846	-12.757	<.001	-1.2446	-0.913
Conf+						
Subject - Final	-0.3548	0.0797	-4.449	<.001	-0.5111	-0.198
Agent - Final	-0.4264	0.0800	-5.331	<.001	-0.5832	-0.270
Conf-						
Subject - Final	-0.2957	0.0790	-3.744	<.001	-0.4505	-0.141
Agent - Final	-0.4842	0.0796	-6.084	<.001	-0.6401	-0.328
MSe+						
Subject - Final	-0.4831	0.0792	-6.097	<.001	-0.6384	-0.328
Agent - Final	-0.5921	0.0796	-7.438	<.001	-0.7481	-0.436
MSe-						
Subject - Final	-0.3324	0.0793	-4.192	<.001	-0.4879	-0.177
Agent - Final	-0.4990	0.0798	-6.254	<.001	-0.6554	-0.343
AE						
Subject - Final	-0.2859	0.0793	-3.603	<.001	-0.4414	-0.130
Agent - Final	-0.4045	0.0797	-5.077	<.001	-0.5607	-0.248
N. obs	29346					
N. groups	46					

Supplementary Table S4b. Simple effects of Model 1.1b (first third of block trials discarded).

Dv: Accuracy	B	Std. Err.	z	p	95% Conf. Inter.	
Acc+						
Final - Optimal	-0.5337	0.0677	-7.887	<.001	-0.666	-0.40110
Acc-						
Final - Optimal	-0.0270	0.0644	-0.419	0.675	-0.153	0.09919
Conf+						
Final - Optimal	-0.1191	0.0658	-1.811	0.070	-0.248	0.00980
Conf-						
Final - Optimal	-0.1950	0.0655	-2.978	0.003	-0.323	-0.06667
MSe+						
Final - Optimal	-0.3850	0.0659	-5.842	<.001	-0.514	-0.25582
MSe-						
Final - Optimal	0.0108	0.0657	0.165	0.869	-0.118	0.13963
AE						
Final - Optimal	-0.1643	0.0658	-2.499	0.012	-0.293	-0.03545
N. obs	29346					
N. groups	46					

Supplementary Table S5. Simple effects of Model 1.2 (all trials).

Dv: Accuracy	B	Std. Err.	z	p	95% Conf. Inter.	
Acc+						
Final - Optimal	0.4650	0.0825	5.633	<.001	0.3032	0.627
Acc-						
Final - Optimal	0.0373	0.0788	0.473	0.636	-0.1171	0.192
Conf+						
Final - Optimal	0.1297	0.0805	1.611	0.107	-0.0281	0.288
Conf-						
Final - Optimal	0.1737	0.0802	2.165	0.030	0.0165	0.331
MSe+						
Final - Optimal	0.3797	0.0811	4.683	<.001	0.2208	0.539
MSe-						
Final - Optimal	-0.0260	0.0805	-0.322	0.747	-0.1838	0.132
AE						
Final - Optimal	0.1946	0.0806	2.416	0.016	0.0367	0.353
N. obs	19652					
N. groups	46					

Supplementary Table S5b. Simple effects of Model 1.2b (first third of block trials discarded).

Dv: Influence	B	Std. Err.	t	p	95% Conf. Inter.	
Intercept	0.3732	0.0142	26.35	<.001	0.3454	0.4010
Δ mean accuracy	0.2922	0.0204	14.30	<.001	0.2521	0.3322
Δ mean confidence	0.2750	0.0260	10.59	<.001	0.2241	0.3259
Δ metacognitive sensitivity	0.0463	0.0114	4.06	<.001	0.0239	0.0686
Δ confidence normalized	0.5051	0.0102	49.73	<.001	0.4852	0.5250
Δ metacognitive sensitivity* Δ confidence normalized	0.1044	0.0403	2.59	0.010	0.0254	0.1835
N. obs	9192					
N. groups	46					

Supplementary Table S6. Fixed effects of Model 1.3.

Dv: Influence	B	Std. Err.	t	p	95% Conf. Inter.	
Intercept	0.5337	0.01055	50.60	<.001	0.5131	0.5544
Influence type (real - optimal)	-0.1642	0.01378	-11.92	<.001	-0.1912	-0.1372
Δ mean accuracy	0.8528	0.01940	43.96	<.001	0.8148	0.8908
Δ mean confidence	0.1250	0.02423	5.16	<.001	0.0775	0.1725
Δ metacognitive sensitivity	-0.0747	0.01080	-6.92	<.001	-0.0959	-0.0535
Δ confidence normalized	0.7180	0.00957	75.02	<.001	0.6993	0.7368
Δ metacognitive sensitivity* Δ confidence normalized	0.3020	0.03865	7.81	<.001	0.2263	0.3778
Δ mean accuracy*influence type	-0.5615	0.02765	-20.31	<.001	-0.6157	-0.5073
Δ mean confidence*influence type	0.1485	0.03487	4.26	<.001	0.0801	0.2168
Δ metacognitive sensitivity* influence type	0.1197	0.01540	7.78	<.001	0.0895	0.1499
Δ confidence normalized* influence type	-0.2102	0.01370	-15.35	<.001	-0.2371	-0.1834
Δ metacognitive sensitivity* Δ confidence normalized* influence type	-0.1974	0.05489	-3.60	<.001	-0.3050	-0.0898
N. obs	18760					
N. groups	46					

Supplementary Table S7. Fixed effects of Model 1.4. Influence type (real vs optimal) is treated as a dummy variable (reference level: optimal).

Dv: Influence	B	Std. Err.	t	p	95% Conf. Inter.	
Intercept	0.3685	0.01056	34.89	<.001	0.3478	0.3892
Influence type (optimal-real)	0.1659	0.00367	45.24	<.001	0.1587	0.1731
Δ mean accuracy	0.2865	0.01965	14.58	<.001	0.2480	0.3250
Δ mean confidence	-0.2318	0.02678	-8.65	<.001	-0.2843	-0.1793
Δ metacognitive sensitivity	0.0443	0.01089	4.06	<.001	0.0229	0.0656
Δ confidence	0.5086	0.00990	51.39	<.001	0.4892	0.5280
Δ metacognitive sensitivity* Δ confidence	0.1184	0.03816	3.10	0.002	0.0436	0.1932
Δ mean accuracy*influence type	0.5726	0.02696	21.24	<.001	0.5198	0.6255
Δ mean confidence*influence type	-0.3487	0.03688	-9.45	<.001	-0.4209	-0.2764
Δ metacognitive sensitivity* influence type	-0.1222	0.01490	-8.20	<.001	-0.1514	-0.0930
Δ confidence * influence type	0.2092	0.01375	15.21	<.001	0.1823	0.2362
Δ metacognitive sensitivity* Δ confidence * influence type	0.1653	0.05355	3.09	0.002	0.0604	0.2703
N. obs	18760					
N. groups	46					

Supplementary Table S8. Fixed effects of Model 1.4b. Influence type (real vs optimal) is treated as a dummy variable (reference level: real).

Dv: Influence	B	Std. Err.	t	p	95% Conf. Inter.	
Intercept	0.3701	0.0150	24.686	<.001	0.3407	0.3995
Influence type (optimal-real)	0.1628	0.0137	11.911	<.001	0.1360	0.1896
Δ mean accuracy	0.2652	0.0196	13.502	<.001	0.2267	0.3037
Δ mean confidence	0.2566	0.0249	10.285	<.001	0.2077	0.3055
Δ metacognitive sensitivity	0.0456	0.0109	4.173	<.001	0.0242	0.0671
Norm. confidence subject	-0.5795	0.0141	-41.062	<.001	-0.6072	-0.5519
Norm. confidence agent	0.4350	0.0142	30.741	<.001	0.4073	0.4628
Δ metacognitive sensitivity* Norm. confidence subject	0.0303	0.0588	0.515	0.607	-0.0850	0.1455
Δ metacognitive sensitivity* Norm. confidence agent	0.2307	0.0563	4.097	<.001	0.1203	0.3411
Δ mean accuracy*influence type	0.5571	0.0275	20.254	<.001	0.5032	0.6110
Δ mean confidence*influence type	-0.1549	0.0347	-4.468	<.001	-0.2228	-0.0869
Δ metacognitive sensitivity* influence type	-0.1187	0.0153	-7.753	<.001	-0.1487	-0.0887
Norm. confidence subject * influence type	-0.0546	0.0197	-2.779	0.005	-0.0932	-0.0161
Norm. confidence agent* influence type	0.3683	0.0199	18.491	<.001	0.3292	0.4073
Δ metacognitive sensitivity* Norm. confidence subject * influence type	0.3282	0.0820	4.001	<.001	0.1674	0.4889
Δ metacognitive sensitivity* Norm. confidence agent * influence type	0.7319	0.0798	9.175	<.001	0.5755	0.8882
N. obs	18760					
N. groups	46					

Supplementary Table S9. Fixed effects of Model 1.4c. Influence type (real vs optimal) is treated as a dummy variable (reference level: real).

Dv: accuracy inter-block estimate	B	Std. Err.	t	p	95% Conf. Inter.	
Self	0.646	0.0578	11.17	<.001	0.533	0.760
Adviser	0.494	0.0511	9.67	<.001	0.394	0.595
N. obs	644					
N. groups	46					

Supplementary Table S10. Simple effects of Model 1.5.

Dv: accuracy inter-block estimate	B	Std. Err.	t	p	95% Conf. Inter.	
Acc+	9.96	2.56	3.886	<.001	4.92	14.989
Acc-	-5.96	2.56	-2.325	0.020	-10.99	-0.924
Conf+	3.22	2.56	1.256	0.210	-1.82	8.250
Conf-	-3.11	2.56	-1.213	0.226	-8.14	1.924
MSe+	6.70	2.56	2.613	0.009	1.66	11.728
MSe-	-4.20	2.56	-1.637	0.102	-9.23	0.837
AE	2.33	2.56	0.908	0.364	-2.71	7.359
N. obs	644					
N. groups	46					

Supplementary Table S11. Simple effects of Model 1.6.

Dv: mean confidence inter-block estimate	B	Std. Err.	t	p	95% Conf. Inter.	
Self	0.745	0.0568	13.1	<.001	0.633	0.856
Adviser	0.483	0.0460	10.5	<.001	0.392	0.573
N. obs	644					
N. groups	46					

Supplementary Table S12. Simple effects of Model 1.7.

Dv: mean confidence inter-block estimate	B	Std. Err.	t	p	95% Conf. Inter.	
Acc+	2.80	2.24	1.254	0.210	-1.59	7.195
Acc-	-5.54	2.24	-2.480	0.013	-9.93	-1.153
Conf+	3.17	2.24	1.420	0.156	-1.22	7.565
Conf-	-12.35	2.24	-5.523	<.001	-16.74	-7.957
MSe+	1.17	2.24	0.525	0.600	-3.22	5.565
MSe-	-5.26	2.24	-2.353	0.019	-9.65	-0.870
AE	-2.59	2.24	-1.157	0.248	-6.98	1.804
N. obs	644					
N. groups	46					

Supplementary Table S13. Simple effects of Model 1.8.

Dv: metacognitive sensitivity inter-block estimate	B	Std. Err.	t	p	95% Conf. Inter.	
Self	0.259	0.144	1.79	0.073	-0.0245	0.543
Adviser	0.338	0.105	3.23	0.001	0.1325	0.544
N. obs	644					
N. groups	46					

Supplementary Table S14. Simple effects of Model 1.9.

Dv: mean confidence inter-block estimate	B	Std. Err.	t	p	95% Conf. Inter.	
Acc+	-2.717	3.43	-0.7932	0.428	-9.45	4.011
Acc-	-4.630	3.43	-1.3517	0.177	-11.36	2.098
Conf+	1.391	3.43	0.4061	0.685	-5.34	8.119
Conf-	-6.413	3.43	-1.8721	0.062	-13.14	0.315
MSe+	4.065	3.43	1.1867	0.236	-2.66	10.793
MSe-	0.326	3.43	0.0952	0.924	-6.40	7.054
AE	4.130	3.43	1.2057	0.228	-2.60	10.859
N. obs	644					
N. groups	46					

Supplementary Table S15. Simple effects of Model 1.10.

Experiment 2

Adviser	Initial accuracy (subjects)	Final accuracy (subjects)	Optimal accuracy (Bayesian model)
**Acc+**	0.39 (0.14)	0.56 (0.16)	0.66 (0.14)
**Acc-**	0.45 (0.13)	0.47 (0.19)	0.47 (0.18)
**Conf+**	0.45 (0.15)	0.55 (0.18)	0.57 (0.16)
**Conf-**	0.45 (0.14)	0.53 (0.19)	0.56 (0.16)
**MSe+**	0.43 (0.15)	0.58 (0.18)	0.64 (0.19)
**MSe-**	0.43 (0.16)	0.52 (0.20)	0.55 (0.19)
**AE**	0.43 (0.14)	0.52 (0.19)	0.56 (0.18)
**All**	0.43 (0.12)	0.53 (0.17)	0.57 (0.15)

Supplementary Table S16. Descriptive statistics reporting the average accuracy of participants' initial judgment, final decision, and accuracy of Bayesian model optimal decision, by adviser type. Between-subject standard deviations in brackets.

Adviser	Observed influence (subjects)	Optimal influence (Bayesian model)
**Acc+**	0.43 (0.11)	0.70 (0.11)
**Acc-**	0.26 (0.11)	0.29 (0.11)
**Conf+**	0.38 (0.12)	0.53 (0.12)
**Conf-**	0.32 (0.10)	0.56 (0.12)
**MSe+**	0.37 (0.11)	0.51 (0.10)
**MSe-**	0.35 (0.13)	0.51 (0.16)
**AE**	0.38 (0.11)	0.52 (012)
**All**	0.35 (0.09)	0.51 (0.07)

Supplementary Table S17. Descriptive statistics reporting the average influence of participants (observed influence) and average influence suggested by the Bayesian model optimal decision (optimal influence), by adviser type. Between-subject standard deviations in brackets.

Dv: Accuracy	df	X^2	p
Decision type	2	483	< 0.001
Adviser type	6	284	< 0.001
Decision type *Adviser type	12	461	< 0.001
N. obs	40839		
N. groups	43		

Supplementary Table S18. Omnibus results of Model 2.1

Dv: Accuracy	B	Std. Err.	z	p	95% Conf. Inter.	
Acc+						
Subject - Final	-0.751	0.0691	-10.86	<.001	-0.8860	-0.6150
Agent - Final	0.183	0.0688	2.66	0.008	0.0482	0.3180
Acc-						
Subject - Final	-0.108	0.0643	-1.67	0.094	-0.2338	0.0184
Agent - Final	-1.244	0.0710	-17.52	<.001	-1.3835	-1.1052
Conf+						
Subject - Final	-0.422	0.0672	-6.28	<.001	-0.5532	-0.2899
Agent - Final	-0.579	0.0675	-8.57	<.001	-0.7111	-0.4465
Conf-						
Subject - Final	-0.328	0.0669	-4.90	<.001	-0.4591	-0.1968
Agent - Final	-0.427	0.0671	-6.37	<.001	-0.5591	-0.2959
MSe+						
Subject - Final	-0.632	0.0670	-9.43	<.001	-0.7638	-0.5010
Agent - Final	-0.680	0.0672	-10.12	<.001	-0.8115	-0.5482
MSe-						
Subject - Final	-0.393	0.0670	-5.87	<.001	-0.5242	-0.2617
Agent - Final	-0.477	0.0672	-7.10	<.001	-0.6092	-0.3457
AE						
Subject - Final	-0.386	0.0670	-5.77	<.001	-0.5174	-0.2549
Agent - Final	-0.464	0.0672	-6.90	<.001	-0.5953	-0.3320
N. obs	40839					
N. groups	43					

Supplementary Table S19. Simple effects of Model 2.1.

Dv: Accuracy	B	Std. Err.	z	p	95% Conf. Inter.	
Acc+						
Final - Optimal	-0.4658	0.0711	-6.554	<.001	-0.605	-0.32649
Acc-						
Final - Optimal	0.0278	0.0653	0.425	0.671	-0.100	0.15583
Conf+						
Final - Optimal	-0.0842	0.0684	-1.232	0.218	-0.218	0.04980
Conf-						
Final - Optimal	-0.1296	0.0680	-1.906	0.057	-0.263	0.00370
MSe+						
Final - Optimal	-0.2755	0.0690	-3.993	<.001	-0.411	-0.14028
MSe-						
Final - Optimal	-0.1082	0.0679	-1.595	0.111	-0.241	0.02474
AE						
Final - Optimal	-0.1591	0.0679	-2.343	0.019	-0.292	-0.02598
N. obs	27226					
N. groups	43					

Supplementary Table S20. Simple effects of Model 2.2.

Dv: Influence	B	Std. Err.	t	p	95% Conf. Inter.	
Intercept	0.3559	0.01326	26.84	<.001	0.3299	0.3819
Δ mean accuracy	0.3734	0.01823	20.48	<.001	0.3377	0.4091
Δ mean confidence	0.1746	0.02041	8.55	<.001	0.1345	0.2146
Δ metacognitive sensitivity	0.0348	0.01096	3.17	0.002	0.0133	0.0562
Δ confidence normalized	0.5125	0.00814	62.93	<.001	0.4965	0.5285
Δ metacognitive sensitivity* Δ confidence normalized	0.1475	0.03819	3.86	<.001	0.0727	0.2224
N. obs	12858					
N. groups	43					

Supplementary Table S21. Fixed effects of Model 2.3.

Dv: Influence	B	Std. Err.	t	p	95% Conf. Inter.	
Intercept	0.5135	0.00915	56.15	<.001	0.4955	0.53139
Influence type (real - optimal)	-0.1576	0.01409	-11.18	<.001	-0.1852	-0.12997
Δ mean accuracy	1.0029	0.01784	56.23	<.001	0.9680	1.03789
Δ mean confidence	-0.0553	0.01991	-2.78	0.005	-0.0943	-0.01630
Δ metacognitive sensitivity	-0.0177	0.01074	-1.65	0.100	-0.0387	0.00338
Δ confidence normalized	0.5755	0.00798	72.14	<.001	0.5599	0.59115
Δ metacognitive sensitivity* Δ confidence normalized	0.5216	0.03768	13.84	<.001	0.4477	0.59542
Δ mean accuracy*influence type	-0.6294	0.02541	-24.77	<.001	-0.6793	-0.57963
Δ mean confidence*influence type	0.2298	0.02842	8.08	<.001	0.1740	0.28546
Δ metacognitive sensitivity* influence type	0.0526	0.01529	3.44	<.001	0.0226	0.08255
Δ confidence normalized* influence type	-0.0631	0.01136	-5.55	<.001	-0.0853	-0.04078
Δ metacognitive sensitivity* Δ confidence normalized* influence type	-0.3740	0.05348	-6.99	<.001	-0.4788	-0.26919
N. obs	26176					
N. groups	43					

Supplementary Table S22. Fixed effects of Model 2.4. Influence type (real vs optimal) is treated as a dummy variable (reference level: optimal).

Dv: Influence	B	Std. Err.	t	p	95% Conf. Inter.	
Intercept	0.3553	0.01326	26.79	<.001	0.3293	0.3812
Influence type (optimal-real)	0.1580	0.01408	11.22	<.001	0.1304	0.1856
Δ mean accuracy	0.3734	0.01812	20.61	<.001	0.3379	0.4089
Δ mean confidence	-0.3387	0.02175	-15.57	<.001	-0.3813	-0.2961
Δ metacognitive sensitivity	0.0323	0.01090	2.96	0.003	0.0109	0.0536
Δ confidence	0.5179	0.00798	64.94	<.001	0.5022	0.5335
Δ metacognitive sensitivity* Δ confidence	0.1291	0.03745	3.45	<.001	0.0557	0.2025
Δ mean accuracy*influence type	0.6290	0.02542	24.74	<.001	0.5791	0.6788
Δ mean confidence*influence type	-0.2920	0.03049	-9.58	<.001	-0.3518	-0.2322
Δ metacognitive sensitivity* influence type	-0.0599	0.01531	-3.91	<.001	-0.0899	-0.0298
Δ confidence * influence type	0.0768	0.01120	6.86	<.001	0.0549	0.0988
Δ metacognitive sensitivity* Δ confidence * influence type	0.3783	0.05272	7.18	<.001	0.2750	0.4816
N. obs	26176					
N. groups	43					

Supplementary Table S23. Fixed effects of Model 2.3b. Influence type (real vs optimal) is treated as a dummy variable (reference level: real).

Dv: Influence	B	Std. Err.	t	p	95% Conf. Inter.	
Intercept	0.43551	0.00895	48.665	<.001	0.41797	0.45305
Influence type (optimal-real)	0.15847	0.01407	11.266	<.001	0.13090	0.18605
Δ mean accuracy	0.68552	0.01249	54.883	<.001	0.66104	0.71001
Δ mean confidence	0.06144	0.01396	4.400	<.001	0.03407	0.08881
Δ metacognitive sensitivity	0.00505	0.00752	0.672	0.502	-0.00968	0.01978
Norm. confidence subject	-0.60181	0.00785	-76.657	<.001	-0.61719	-0.58642
Norm. confidence agent	0.50878	0.00774	65.733	<.001	0.49361	0.52395
Δ metacognitive sensitivity* Norm. confidence subject	0.34309	0.03777	9.084	<.001	0.26906	0.41712
Δ metacognitive sensitivity* Norm. confidence agent	0.98262	0.03671	26.767	<.001	0.91067	1.05457
Δ mean accuracy*influence type	0.62636	0.02497	25.088	<.001	0.57742	0.67529
Δ mean confidence*influence type	-0.23501	0.02792	-8.417	<.001	-0.28973	-0.18028
Δ metacognitive sensitivity* influence type	-0.05551	0.01502	-3.696	<.001	-0.08495	-0.02607
Norm. confidence subject * influence type	-0.03294	0.01570	-2.098	0.036	-0.06371	-0.00217
Norm. confidence agent* influence type	0.11492	0.01548	7.424	<.001	0.08458	0.14527
Δ metacognitive sensitivity* Norm. confidence subject * influence type	0.48142	0.07553	6.374	<.001	0.33337	0.62946
Δ metacognitive sensitivity* Norm. confidence agent * influence type	1.18650	0.07342	16.161	<.001	1.04259	1.33040
N. obs	26176					
N. groups	43					

Supplementary Table S24. Fixed effects of Model 2.4c. Influence type (real vs optimal) is treated as a dummy variable (reference level: real).

Dv: Influence	B	Std. Err.	t	p	95% Conf. Inter.	
Intercept	0.66413	0.00950	69.924	<.001	0.6455	0.68275
Treatment (Exp2-Exp1)	0.01374	0.01900	0.723	0.471	-0.0235	0.05098
Δ mean accuracy	-0.23366	0.01479	-15.800	<.001	-0.2626	-0.20467
Δ mean confidence	0.10339	0.01760	5.875	<.001	0.0689	0.13788
Δ metacognitive sensitivity	0.04751	0.00855	5.557	<.001	0.0308	0.06426
Δ confidence normalized	-0.09554	0.00695	-13.739	<.001	-0.1092	-0.08191
Δ metacognitive sensitivity* Δ confidence	-0.06476	0.03026	-2.140	0.032	-0.1241	-0.00546
Δ mean accuracy*treatment	-0.06209	0.02958	-2.099	0.036	-0.1201	-0.00412
Δ mean confidence*treatment	-0.02688	0.03520	-0.764	0.445	-0.0959	0.04211
Δ metacognitive sensitivity*treatment	-0.01086	0.01710	-0.635	0.525	-0.0444	0.02265
Δ confidence normalized*treatment	0.04644	0.01391	3.339	<.001	0.0192	0.07370
Δ metacognitive sensitivity* Δ confidence normalized*treatment	0.00843	0.06051	0.139	0.889	-0.1102	0.12704
N. obs	23394					
N. groups	89					

Supplementary Table S25. Fixed effects of Model 3.1. Treatment (experiment 1 vs experiment 2) is treated as a dummy variable (reference level: experiment 1).

Dv: Influence	B	Std. Err.	t	p	95% Conf. Inter.	
Intercept	0.44325	0.00700	63.283	<.001	0.42953	0.45698
MSG*influence type	-0.01286	0.01975	-0.651	0.517	-0.05156	0.02585
Δ mean accuracy*MSG*influence type	-0.13099	0.03744	-3.498	<.001	-0.20438	-0.05760
Δ mean confidence*MSG*influence type	-0.04051	0.04413	-0.918	0.359	-0.12701	0.04599
Δ metacognitive sensitivity*MSG*influence type	-0.05849	0.02176	-2.688	0.007	-0.10114	-0.01583
Δ confidence normalized*MSG*influence type	-0.06288	0.01760	-3.572	<.001	-0.09738	-0.02838
Δ metacognitive sensitivity*Δ confidence normalized*MSG*influence type	0.11727	0.07781	1.507	0.132	-0.03525	0.26978
N. obs	44936					
N. groups	89					

Supplementary Table S26. Fixed effects of Model 3.2. We report only the interactions with the factor MSG*influence type. Influence type (real vs optimal) is treated as a dummy variable (reference level: optimal). Metacognitive sensitivity Group (MSG) is treated as a dummy variable (reference level: High MSG).

Dv: Influence	B	Std. Err.	t	p	95% Conf. Inter.	
Intercept	0.44297	0.00817	54.217	<.001	0.42696	0.45898
Influence type	-0.16276	0.00254	-64.164	<.001	-0.16773	-0.15779
MSG	-0.00577	0.01634	-0.353	0.725	-0.03780	0.02625
Norm. confidence subject	-0.61133	0.00676	-90.385	<.001	-0.62459	-0.59808
Norm. confidence agent	0.53660	0.00671	80.004	<.001	0.52346	0.54975
Influence type*MSG	-0.00395	0.00507	-0.779	0.436	-0.01390	0.00599
Influence type*Norm. confidence subject	0.07572	0.01343	5.636	<.001	0.04938	0.10205
Norm. confidence agent	-0.20446	0.01341	-15.251	<.001	-0.23074	-0.17819
MSG*Norm. confidence agent	-0.10856	0.01341	-8.093	<.001	-0.13485	-0.08226
MSG*Norm. confidence subject	0.10380	0.01353	7.673	<.001	0.07728	0.13031
Influence type*MSG*Norm. confidence agent	-0.11840	0.02681	-4.416	<.001	-0.17096	-0.06585
Influence type*MSG*Norm. confidence subject	0.05823	0.02687	2.167	0.030	0.00557	0.11090
N. obs	44936					
N. groups	89					

Supplementary Table S27. Fixed effects of Model 3.3. Influence type (real vs optimal) is treated as a dummy variable (reference level: optimal). Metacognitive sensitivity Group (MSG) is treated as a dummy variable (reference level: High MSG).

Dv: Influence	B	Std. Err.	t	p	95% Conf. Inter.	
Intercept	0.44299	0.00699	63.377	<.001	0.42929	0.45669
OVG*influence type	-0.03000	0.00479	-6.258	<.001	-0.03939	-0.02060
Δ mean accuracy* OVG*influence type	-0.15726	0.03695	-4.256	<.001	-0.22968	-0.08484
Δ mean confidence* OVG*influence type	-0.08715	0.04419	-1.972	0.049	-0.17376	-5.37e−4
Δ metacognitive sensitivity* OVG*influence type	-0.02613	0.02137	-1.223	0.221	-0.06802	0.01575
Δ confidence normalized OVG*influence type	-0.03495	0.01762	-1.984	0.047	-0.06948	-4.21e−4
Δ metacognitive sensitivity* Δ confidence normalized* OVG*influence type	-0.13796	0.07883	-1.750	0.080	-0.29246	0.01655
N. obs	44936					
N. groups	89					

Supplementary Table S28. Fixed effects of Model 3.4. We report only the interactions with the factors OVG*influence type. Influence type (real vs optimal) is treated as a dummy variable (reference level: optimal). Overconfidence Group (OVG) is treated as a dummy variable (reference level: Low OVG).

Dv: Influence	B	Std. Err.	t	p	95% Conf. Inter.	
Intercept	0.44296	0.00817	54.2387	<.001	0.4270	0.4590
Influence type	-0.16322	0.00254	-64.2535	<.001	-0.1682	-0.1582
OVG	7.80e-4	0.01633	0.0477	0.962	-0.0312	0.0328
Norm. confidence subject	-0.61613	0.00674	-91.4070	<.001	-0.6293	-0.6029
Norm. confidence agent	0.53895	0.00678	79.5466	<.001	0.5257	0.5522
Influence type*OVG	-0.03904	0.00508	-7.6841	<.001	-0.0490	-0.0291
Influence type*Norm. confidence subject	0.07078	0.01339	5.2855	<.001	0.0445	0.0970
Norm. confidence agent	-0.20857	0.01354	-15.4011	<.001	-0.2351	-0.1820
OVG*Norm. confidence agent	-0.00882	0.01355	-0.6512	0.515	-0.0354	0.0177
OVG*Norm. confidence subject	0.04066	0.01348	3.0160	0.003	0.0142	0.0671
Influence type*OVG*Norm. confidence agent	-0.13029	0.02709	-4.8104	<.001	-0.1834	-0.0772
Influence type*OVG*Norm. confidence subject	-0.03603	0.02678	-1.3455	0.178	-0.0885	0.0165
N. obs	44936					
N. groups	89					

Supplementary Table S29. Fixed effects of Model 3.5. Influence type (real vs optimal) is treated as a dummy variable (reference level: optimal). Overconfidence Group (OVG) is treated as a dummy variable (reference level: Low OVG).

Secondary/exploratory endpoints

Alternative measures of metacognitive ability

To assess the robustness of our metacognitive sensitivity measure, we conducted additional analyses using meta-I (Dayan, 2023) in place of point biserial correlation. Meta-*I*, as the point biserial correlation, aims to quantify metacognitive sensitivity but relies on different methodological assumptions (e.g., the relation is not assumed to be linear) and a different theoretical background (information theory). We used meta-I_2^r (Dayan, 2023) as a measure of metacognitive efficiency, a measure of metacognitive ability independent of first-order performance.

To calculate meta-*I*, we discretized our confidence measure (0-100 scale) into 20 confidence intervals. Given that our task relies on a continuous response space, given binary accuracy (a) and discretized confidence (c), we calculated mutual information as I=H(a)-H(a|c). Absolute efficiency (meta-I_2^r) was computed as I/H(a) to control for unconditional accuracy entropy.

Participants' meta-*I* and meta-I_2^r significantly correlate with their individual metacognitive sensitivity (Meta-I: rho=0.73, p < 0.001, meta-I^r_2: rho=0.70, p < 0.001). Moreover, our agent manipulation based on metacognitive sensitivity (i.e., agents MSe+ and MSe-) was in line with variations in meta-*I* and meta-I_2^r. Regressions with meta-*I* and meta-I_2^r as dependent variables and agent (MSe+, MSe-, and AE as a reference level) show that MSe+ has *higher* metacognitive sensitivity and efficiency than the AE agent (Meta-*I*: B=0.31, p < 0.001; meta-I_2^r: B=0.33, p < 0.001), while MSe- has *lower* metacognitive sensitivity and efficiency than the AE agent (meta-*I*: B=-0.05, p < 0.001; meta-I_2^r: B=-0.05, p < 0.001).

Temporal and learning effects

In Experiment 1, participants were expected to acquire information about each adviser's skills through trial-by-trial feedback, whereas in Experiment 2, they additionally received explicit information about these skills before starting each experimental block with a given agent. In this section, we examine temporal and learning effects to assess whether (1) in Experiment 1, participants continue to adapt their behavior throughout the entire block (54 trials) or instead stabilize before or during the interaction, and whether (2) the explicit information provided in Experiment 2 alters participants' behavior relative to Experiment 1.
Because participants do not have direct trial-by-trial access to an explicit representation of advisers' attributes, we use trial-by-trial optimality as an index capturing the effects of learning and prior knowledge about adviser competence. We first divided each experimental block (54 trials) into three temporally ordered sub-blocks of 18 trials each and fit a mixed-effects model with participant as a random intercept, using trial-by-trial optimality as the dependent variable and sub-block, experiment, and their interaction as predictors.
The results (Figure S5) revealed a significant effect of sub-block in Experiment 1 ($\chi^2 = 8.73$, $p < 0.001$), driven by a significant increase in optimality between the first and last sub-blocks ($B = 0.02$, $p < 0.001$), with no difference between the middle and last sub-blocks ($B = 0.01$, $p = 0.601$), indicating a stabilization of behavior. In contrast, no effect of sub-block was observed in Experiment 2 ($\chi^2 = 1.12$, $p = 0.327$).

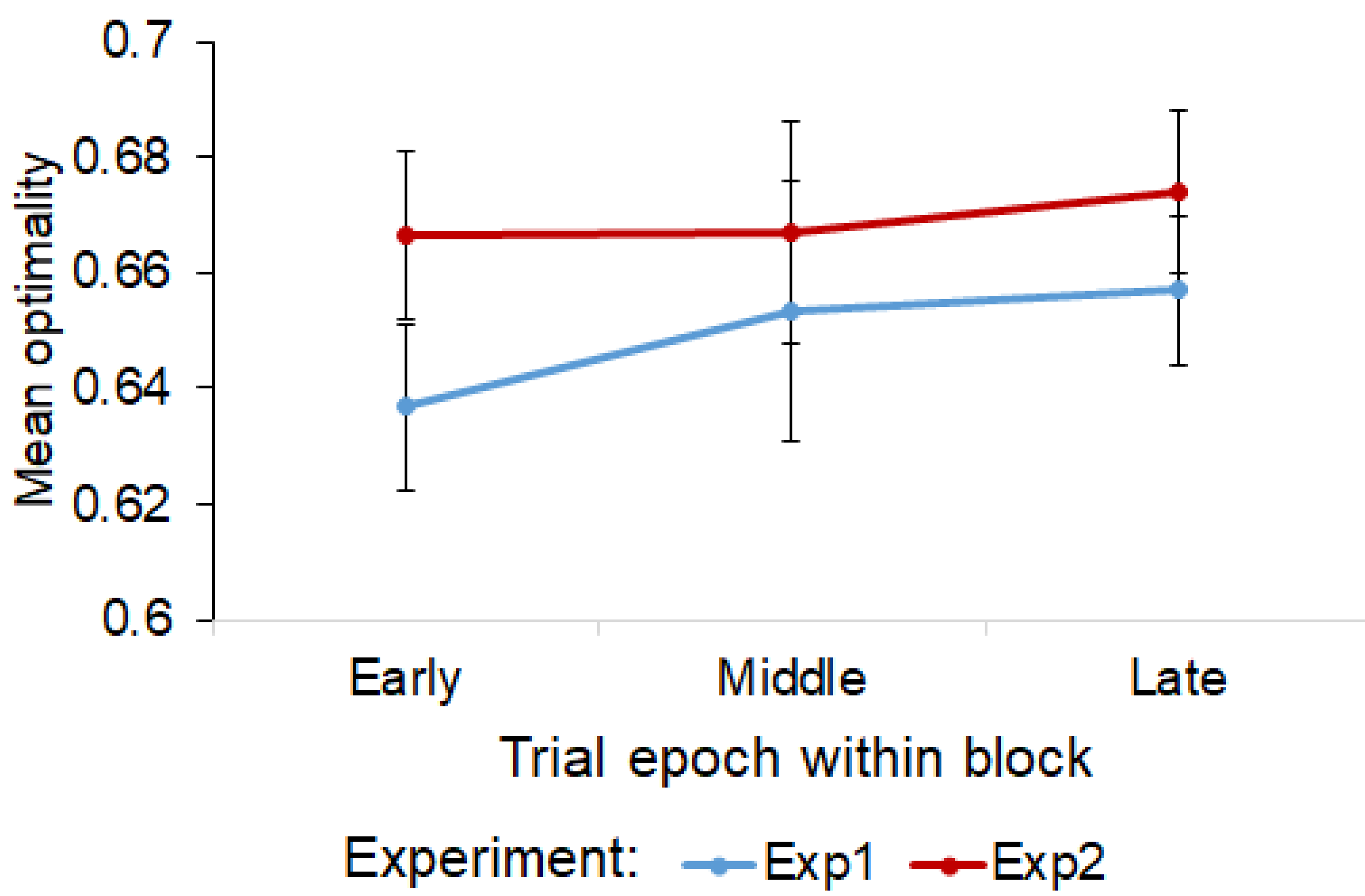

Figure S5. Mean temporal evolution of trial-by-trial optimality across 3 trial epochs (18 trials each) within experimental blocks (Early, Middle, Late) by experiment.

Consistent with these findings, an analysis of the fine-grained temporal evolution of average optimality across trials showed that in Experiment 1, optimality reaches the level observed in Experiment 2 around the beginning of the second third of the block (Figure S5). This convergence supports our analytical approach in the main analyses of Experiment 1, where the first third of trials within each block was excluded to account for learning effects.

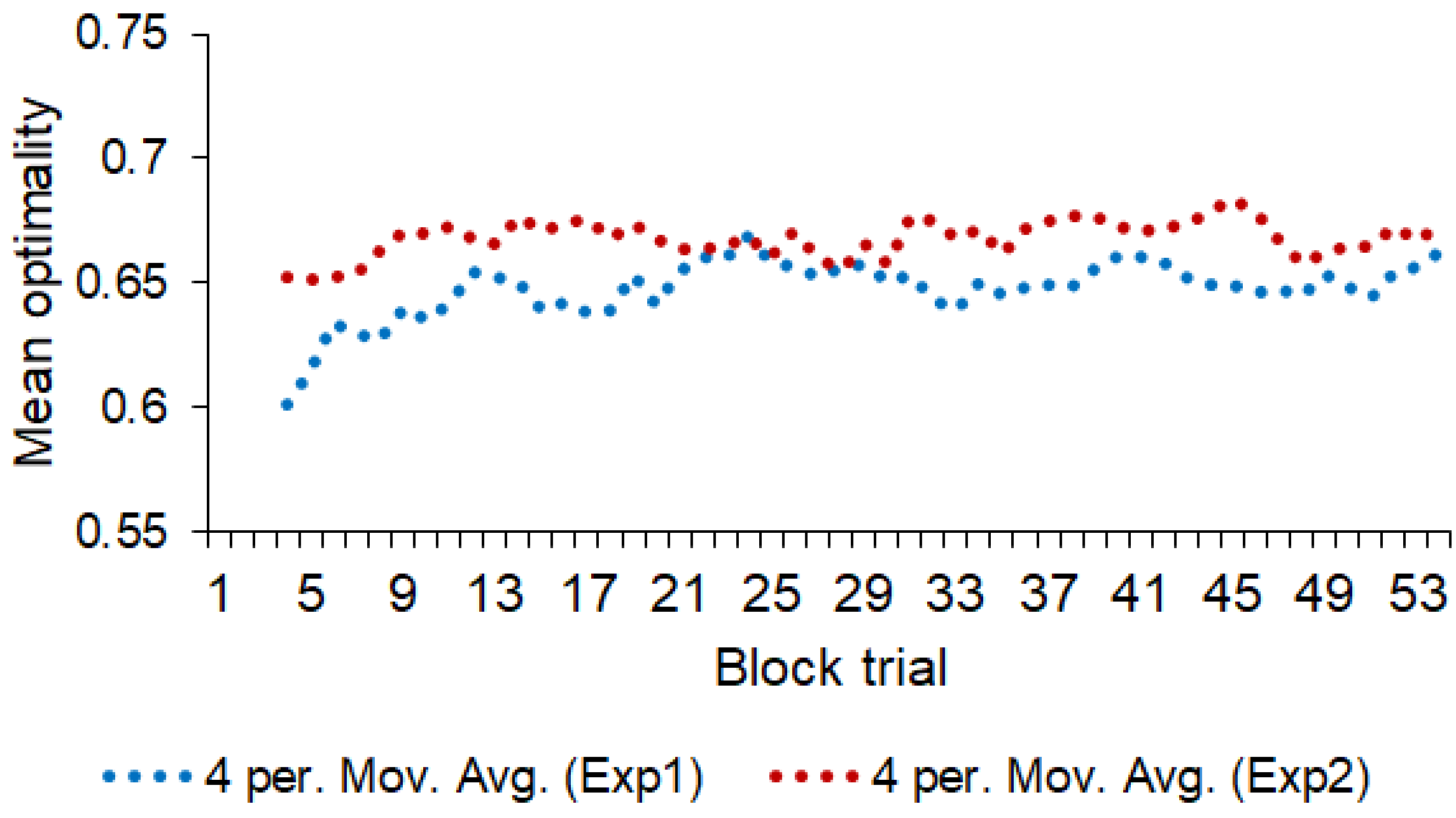

Figure S6. Mean temporal evolution of optimality across trials within experimental blocks using a 4-trials moving average.

Effect of decision maker-adviser consensus on advice use

We observed an overall negative relationship between distance and influence across adviser types, such that lower distance was associated with higher influence ($\beta = -0.01$, $p < 0.001$). This was especially true in trials where the participant was more confident than the agent (interaction: $\beta = 0.03$, $p < 0.001$): the combination of the participant's high confidence and a high distance led to disuse of the advice. However, when including the interaction with adviser type (Supplementary Table S30), the relationship between distance and influence was significantly modulated (omnibus interaction effect: $F = 15.57$, $p < 0.001$). Specifically, negative distance-influence relationships were observed for agents Acc− ($\beta = -0.04$, $p < 0.001$), Conf− ($\beta = -0.02$, $p < 0.001$), and MSe+ ($\beta = -0.02$, $p < 0.001$).

In contrast, the only significant effect in the opposite direction was observed for the high-accuracy adviser (Acc+): when the adviser was more accurate, lower consensus (i.e., greater distance) was associated with increased reliance on advice ($\beta = 0.02$, $p < 0.001$). Together, these results indicate that response distance influences advice weighting not because of mechanical constraints on adjustment opportunities, but because distance itself acts as an informational signal whose interpretation depends on the relative accuracy of the participant and the adviser.

Adviser	Effect	Estimate	SE	z	p	95% Confidence Intervals	
**Acc+**	distance	0.025	0.005	4.565	<.001	0.0142	0.0356
**Acc-**	distance	-0.038	0.005	-7.726	<.001	-0.0473	-0.0282
**Conf+**	distance	0.001	0.006	0.190	0.850	-0.0099	0.0120
**Conf-**	distance	-0.018	0.005	-3.362	<.001	-0.0292	-0.0077
**MSe+**	distance	-0.018	0.005	-3.341	<.001	-0.0282	-0.0073
**MSe-**	distance	0.008	0.006	1.455	0.146	-0.0028	0.0188
**AE**	distance	-0.007	0.005	-1.325	0.185	-0.0176	0.0034

Supplementary Table S30. Simple effects of distance on influence moderated by adviser type. We consider data from both experiments (for Experiment 1, we discard the first third of the trials, in line with the main analyses).

Moreover, we found a significant negative effect of distance on optimality in advice use ($\beta = -0.01$, $p = 0.023$), which was moderated by the difference between the agent's and participant's confidence (interaction: $\beta = 0.01$, $p < 0.001$). When the participant was more confident, stronger consensus increased optimality; conversely, when the agent was more confident, higher consensus decreased optimality.

We also found a significant interaction between distance and adviser type ($F = 12.50$, $p < 0.001$). We found a negative relationship with most of the agents (Acc+: $\beta = -0.01$, $p = 0.026$; Conf+: $\beta = -0.01$, $p = 0.018$; Conf-: $\beta = -0.01$, $p = 0.029$; MSe-: $\beta = -0.02$, $p < 0.001$). The only agent with a *positive* relationship was Acc- ($\beta = 0.03$, $p < 0.001$). These results suggest that stronger consensus enhances optimality when interacting with an adviser of similar (or higher) accuracy, whereas it is detrimental when interacting with a less accurate adviser.

These results suggest that participants use confidence and participant-agent response distance as informational signals to modulate the use of advice, thereby affecting optimality.

Inter-block performance ratings - Experiment 2

Results of inter-block ratings of Experiment 1 revealed that participants provided reasonably good estimates of their own and advisers' performance and mean confidence, but failed to (explicitly) detect differences in advisers' metacognitive sensitivity. Since information disclosure in Experiment 2 was intended to remove ambiguity about participants' and advisers' skills, we expect inter-block ratings in Experiment 2 to better reflect self- and others' estimates of accuracy, mean confidence, and metacognitive sensitivity.

As in Experiment 1, participants noticed that Acc+ and Acc- differed in accuracy ($B=21.23$, $p<0.001$). Moreover, they correctly identified the agent Acc+ as more accurate than them (Adviser - Self: $B=9.40$, $p<0.001$) and the agent Acc- as less accurate than them (Adviser - Self: $B=-11.47$, $p<0.001$).

Similarly, participants realized that Conf+ and Conf- differ in terms of mean confidence ($B=19.23$, $p<0.001$). Moreover, they correctly identified the agent Conf+ as more confident, on average, than themselves (Adviser - Self: $B=8.02$, $p<0.001$) and the agent Conf- as less confident than them (Adviser - Self: $B=-11.88$, $p=0.020$).

Importantly, in Experiment 1, participants' inter-block ratings could *not* distinguish agents in terms of metacognitive sensitivity. Conversely, in Experiment 2, they correctly identified the metacognitive

sensitivity of agent MSe+ to be higher than that of MSe- ($B=0.09$, $p=0.007$) and realized that the confidence of agent MSe+ was higher than their own (Adviser - Self: $B=0.11$, $p=0.001$).
Overall, these results reveal that the additional information provided in Experiment 2 successfully helped participants identify the relevant agents' attributes, even though it had a marginal impact on the optimality of advice use.

Agent generation - parameter accuracy

Agents were generated to match specific target parameters derived from participants' prior behavior. However, given the limited number of trials per agent ($n = 54$) and the need to generate agents online during the experiment, we allowed some tolerance between the target parameters and those implemented in the generated agents. This tolerance was defined separately for each parameter and increased adaptively during the generation process as the algorithm searched for a solution that best matched all target constraints.
In this section, we quantify the final discrepancy between the target parameters and those actually implemented in the agents. Overall, discrepancies were small. The mean absolute deviation (between-subject SD in parentheses) was 0.031 (0.009) for mean accuracy, 0.017 (0.005) for mean confidence, and 0.057 (0.023) for metacognitive sensitivity. Agents with absolute deviations greater than 0.10 were rare (mean accuracy: 3% of agents; mean confidence: 0%; metacognitive sensitivity: 16%), and became negligible when considering a tolerance of 0.20 (mean accuracy: 0%; mean confidence: 0%; metacognitive sensitivity: 3%).
Importantly, our main analyses included the actual relative differences between each generated agent's parameters and the participant's corresponding parameters as predictors, thereby explicitly controlling for any residual deviation between target and implemented agent behavior.

Interaction with an identical agent

Focusing on the interaction with the Alter Ego agent, results reveal an egocentric bias, with a mean influence of 0.37. Advice use was significantly modulated by the relative difference between the agent's and the participant's normalized confidence (Δ normalized confidence; $B = 0.55$, $p < 0.001$), with no interaction with experiment ($B = -0.01$, $p = 0.837$): the greater the agent's confidence relative to the participant's, the higher the influence.
At the same time, we observed a significant negative relationship between Δ normalized confidence and optimality ($B = -0.07$, $p < 0.001$), again with no effect of experiment ($B = 0.00$, $p = 0.956$). This pattern reflects an egocentric weighting of confidence signals, leading participants to respond suboptimally: they underweight advice when an agent with comparable skill provides higher confidence.

Additional analyses on Reaction times

We report results of tests of hypotheses included in the preregistrations concerning participants' reaction times. Mixed-effects models with trial-by-trial final decision RTs and participant as a random intercept showed significant positive relationships with participants' influence ($\beta = 0.07$, $p < 0.001$), participant-agent response distance ($\beta = 0.26$, $p < 0.001$), and Δ normalized confidence (agent-participant, $\beta = 0.10$, $p < 0.001$). A negative relationship between final decision RTs and optimality was found ($\beta = -0.09$, $p < 0.001$).
No significant effects were found for time spent on the feedback screen ($p > 0.05$).

Deviations from preregistered methods and analyses

All the main methods and analyses have been preregistered in two separate OSF preregistrations, available at this link: https://osf.io/5ekdb/.

The experimental tasks (IDT, ADT, inter-block questions) and the overall design of the final manuscript are fully consistent with the preregistered plans.

Analyses of the Raven APM are not included in the final manuscript. To maintain clarity, coherence, and conciseness, we focused our investigation of inter-individual differences on internal predictors derived directly from behavior in the ADT, as specified in the secondary endpoints of the preregistrations. The Raven APM data were collected and will be analyzed alongside other external individual traits in a separate study.

Details of the Bayesian modeling strictly follow the preregistered specifications.

The independent variable "confidence reliability" has been renamed as "metacognitive sensitivity".

The two artificial agents Rel+ and Rel- have been renamed as MSe+ and MSe-.

The planned analyses were reordered to better align with the manuscript's narrative structure and enhance the clarity of our findings. Supplementary Table S31 reports the correspondence between the model numbering in the final manuscript and that in the preregistration.

**Manuscript model**	**Preregistration model (Exp1)**	**Preregistration model (Exp2)**
1.1/2.1	Secondary endpoints	Secondary endpoints
1.1b/2.1b	Control analyses	Control analyses
1.2/2.2	Secondary endpoints	Secondary endpoints
1.2b/2.2b	Control analyses	Control analyses
1.3/2.3	Model 2	Model 2
1.4/2.4	Model 3	Model 3
1.4b/2.4b	Explorative	Explorative
1.4c/2.4c	Explorative	Explorative
1.5-1.10/2-5-2.10	Secondary endpoints	Secondary endpoints
3.1	NA	Model 4
3.2-2.5	Model 1 and Secondary endpoints	Model 1 and Secondary endpoints
Regression models for inter-individual analysis	Secondary endpoints	Secondary endpoints

Supplementary Table S31. Correspondence between the numbering of the models of the final manuscript and those reported in the preregistration. Models labeled as "exploratory" were not explicitly included in the preregistered analyses and were conducted solely at an exploratory level to provide additional insights into the results of the preregistered models.

Supplementary References

Dayan, P. Metacognitive Information Theory. *Open Mind* **7**, 392–411 (2023).
